# Interpreting materia medica. A case study on Ioannes Archiatrus

**DOI:** 10.12688/wellcomeopenres.20061.2

**Published:** 2024-06-19

**Authors:** Barbara Zipser, Andreas Lardos, Rebecca Lazarou, Robert Allkin, Mark Nesbitt, Andrew C. Scott

**Affiliations:** 1 History, Royal Holloway University of London, Egham, England, TW20 0EX, UK; 2 Natural Product Chemistry and Phytopharmacy, ZHAW Zürcher Hochschule für Angewandte Wissenschaften, Einsiedlerstrasse 29, Zurich, 8820 Wädenswil, Switzerland; 3 Enhanced Partnerships, Royal Botanic Gardens Kew, Richmond, England, TW9 3AE, UK; 4 Digital Revolution, Royal Botanic Gardens Kew, Richmond, England, TW9 3AE, UK; 5 Earth Sciences, Royal Holloway University of London, Egham, England, TW20 0EX, UK

**Keywords:** Medical lexicography, medical history, ethnopharmacology, pharmacognosy, Byzantine medicine, Greek medicine, botany, geology

## Abstract

**Background:**

Premodern medical texts are an invaluable source for scholars from humanities and sciences. However, they are usually not accessible as few scientists with an interest in premodern materia medica are also qualiﬁed philologists. Therefore, a balance has to be struck to translate these texts while preserving information on how reliable we believe a given translation to be. In this paper, we conduct a case study on the vernacular version of Ioannes archiatrus.

**Methods:**

The present study forms part of the output of a multidisciplinary Wellcome Trust Collaborative Award combining humanities and sciences. We deployed a multi-layer tagging system to systematise pharmaceutical terminology and to translate these terms while providing conﬁdence factors for individual words. In a second step, we used AntConc, a freeware concordance software, to analyse our primary source and visualise patterns in the text.

**Results:**

Our methodology created a readable text that made it possible for the reader to check conﬁdence factors. It also allows our translation and tagging to be recycled for further research.

**Conclusions:**

Our methods provide a tool that allows to balance the need to translate and the necessary caution about translated plant and mineral names. Our approach is transferable and it can be modiﬁed to suit the needs of other primary sources.

## Introduction

### The philological perspective

Translations of ancient or medieval Greek medical texts are useful in some respects, as they make these text accessible to wider audiences. This is particularly important as these works bear witness of a wide network of intellectual and commercial exchange that covered most parts of Asia and Europe and larger parts of Africa. Primary sources written in Persian, Arabic, Hebrew, Sanskrit, Latin, Armenian, Georgian, Hebrew, Coptic, and Aramaic to name a few would have to be taken into account just to extricate the closest contacts, let alone Himalayan, Caucasian, or Ethiopian languages and other largely neglected areas of research. And there is no person in existence who could complete such a multilingual task without the support of translations.

Moreover, there is also another group of scholars who usually only have limited proficiency in ancient or medieval languages, namely scientists. Yet these texts are of particular value to medical or pharmaceutical research as they reflect centuries of empirical evaluation.

Some types of content in ancient or medieval medical writings are less challenging to translate, for instance physiological works or descriptions of surgery. ^1^ While some of the underlying ideas differ widely from our modern understanding, for instance the concept of black bile, or *melancholia* in Greek, these matters can mostly be explained in an introduction or appendix. ^2^

However, the most common type of content, therapeutic texts, are not easily translated. These works mostly consist of brief descriptions of a given disease, for instance cough, followed by a compilation of pharmaceutical recipes to treat the respective disorder. And for this second part, the pharmaceutical ingredients, we largely lack reliable knowledge of what these words actually mean.

In the following paper, we propose a novel way of handling the task, demonstrated on a particularly challenging source which has insofar not been translated into a modern language. ^3^ It is the vernacular version of John the Physician’s Therapeutics, which we call JC. ^4^ It is written in a language in flux, a dialect not usually used in writing that would later on become demotic Greek, which is today spoken in the Hellenic world. To add to the complexity, the text verges on the genre of *iatrosophion*, which is more prone to be modified in the course of the transmission than a scholarly text, either by accident or on purpose. Moreover, our primary source is a vernacular translation of an earlier text with an added vernacular commentary. This unique combination makes it also of particular interest to philological research.

A detailed discussion of our pharmacognostic work can be found in a separate article, where the relevant secondary literature is cited and discussed. ^5^

### The scientific perspective

What makes the text especially valuable from the perspective of ethnopharmacological research is the property of being a personal recipe collection composed for wider diffusion in medical practice containing empirical knowledge based on the perceived effectiveness of the mentioned therapies. In these more than 1,400 medicinal recipes the author of the text describes the preparation and application of natural drugs for the therapy of many different ailments. This includes the treatment of skin and muscular-skeletal problems, conditions of the eye, ear, nose, mouth, and throat, as well as ailments in different organs or organ systems especially the respiratory, gastrointestinal, and urogenital tract. Some therapies were also directed at the treatment of neurological conditions, such as headache or migraine, or fevers as well as conditions that could be linked with infectious diseases. A substantial proportion of recipes is dedicated to women’s health and maternity. Often the therapy not only consisted of applying some kind of remedy, but also included detailed dietary advice, sometimes also cupping or blood letting, as well as recommendations to avoid certain environmental conditions like hot sun, wind or smoke. Overall, at least 40 different inorganic substances such as minerals, stones or sea, rain and spring water, and over 80 animal substances of mammal, bird, reptile, fish, mollusc, or insect origin were counted. An impressive list of 194 different plants could be established, from which the herbal materia medica consisting of different plant parts, plant substances, or products of plant origin was derived. For the preparation of the plant-based remedies the herbs were used in dried or fresh form, although this information was not always stated in the recipes. In some cases, the herb was first burnt to ashes and then prepared into a remedy. The most frequently mentioned types of preparation were decoctions, in which the herb was boiled in water, vinegar, or wine, press juices of the fresh herb, or simple mixtures of the powdered dry herb again in water, vinegar, or wine. Sometimes rose water or honey were also used in place of the mentioned solvents. All these preparations could be used either for oral applications in form of hot and cold drinks or as mouth rinse and gargle. Or they were used for topical applications such as poultices, washing, and ointments. Another important type of preparation used externally for affections on the skin was a kind of paste prepared from the fresh or dried herb in most cases with the help of some carrier such as olive oil, honey, or the herbal resin *labdanum*, which itself already has medicinal properties. Enemas or pessaries as well as fumigations for inhalation or topical application were more seldom prepared. Especially elaborate preparations consisted in preparing an electuary which was eaten as a snack and which was prepared by mixing various herbs and spices with honey, dates and pounded almonds or sesame. The majority of these remedies were compound preparations consisting of two or more medicinal herbs. In most cases, these mixtures also contained a substance of animal origin, in particular bee honey or some kind of animal fat. In some cases, minerals were also added to the mixture. The other important share of the herbal remedies were so-called simples, which consisted of only one medicinal herb and which was usually extracted or mixed with one of the above mentioned solvents or carriers. Remedies could of course also be prepared by using only minerals or animal substances, but these preparations are less frequently mentioned. As in most historical or present-day traditional medicinal systems, plants are the most important ingredients.

### The conundrum

While it is clear that JC is of great importance for research in both sciences and humanities, two competing demands have to be balanced. The text needs to be translated to make it accessible, in particular for the scientists on our team, but at the same time, it has to be clear that we are mostly only offering an interpretation of a specific plant or mineral name. We do not use modern scientific plant names for this very reason. We recommend reading the spreadsheet containing the confidence factors while perusing our translation. We strongly discourage any modern-day use of the recipes in our primary source without qualified supervision.

## Methods

In a first step, all pharmaceutical ingredients in the text were separated from surrounding text, assigned a four digit unique identifier, divided into groups of substances and assigned a tag – plants (JCP), animal products (JCA), minerals (JCM), compounds/multiples (JCX), and unclear substances (JCU). We aimed to strip the text back to the basics. The initial, methodological discussions were undertaken by all authors of this study. The philologist on the team, BZ, has received old-fashioned training in Latin and Greek and then expanded on her knowledge by reading an extensive amount of medical primary sources in their original Greek. She translated the text, as far as possible, using her knowledge acquired by the above experience. ^6^

AL, who has Cypriot roots, drew on his modern-day knowledge of botanical nomenclature and his ethnopharmacological field experiences with oral transmission of herbal knowledge to add valuable insights. A second generation Cypriot expatriate with a background in plant science, RL, then checked the translation from her perspective. AS contributed insights into the minerals mentioned in the text, and BA and MN contributed methodological considerations.

The classification process was simple yet effective. Any items that were clearly plants as they were described to have leaves, roots, flowers, or seeds in our primary source were assigned a JCP tag followed by their four digit identifier. Animals and animal products were usually easy to classify as such, even if the precise identification of a given animal was sometimes impossible. For instance, we have abundant evidence from a broad range of genres and across a wide chronological and geographic spectrum that the Greek word πρόβατον and variants thereof describe either goats or sheep. While we are certain that this word describes an animal, we do not know which one precisely. Sometimes, the animals are described in our primary source, for instance τειχοδαίμων, JCA_2703, which is said to be a bird. Here again, while we know it is an animal, we are unable to identify it precisely. The animal names in our primary source mostly fall into these two categories. The remaining, very few words, largely aquatic animals, could be classified as animals through the context in which they occur or through other written sources. These words were then given their JCA tag followed by their unique four digit identifier.

These two steps already removed a significant number of items from our list and left us with three categories, minerals, compounds/multiple, and unknown. Minerals were mainly classified as such through descriptions in other primary sources such as Dioscorides’ *De Materia Medica*, ^7^ and labelled JCM, again followed by their respective four digit identifier. The compounds/multiple category is perhaps the most difficult to capture. Here, we merged items that consisted of multiple components, which were potentially cross category, for instance theriac, with items that could be used or mentioned in multiple ways in the text. For instance, women’s milk, the Greek expression for human breast milk, is mentioned as a pharmaceutical ingredient and also the subject of medicinal treatment. The word woman was therefore given a JCX tag. At this stage, we also established the category other, JCO, that contains items that do not fit into any other category, such as fungi or hot baths for example, or words that do not describe materia medica. This left us with a manageable number of words that needed to be classified. Here, BZ ran these words through a Thesaurus Linguae Graecae (TLG) ^8^ full corpus search to determine whether other medical or non-medical authors perceived these words to describe plants, animals, or minerals. In most cases, this yielded a clear result, and the words were moved into the respective categories. Finally, AL sorted through the remaining items and checked the interim lists so that the vast majority of materia medica could at least be sorted into these broad categories. The few remaining items were classed as unknown.

Throughout, common sense was applied in the classification process. Bread was, for instance, classed as a plant rather than a compound, even though the dough was presumably mixed with water, which has a mineral classification, and a leavening agent, which would have been JCO. Following the same pragmatic line of thought, honey was classed as an animal product. The translation could now be updated, and unique four digit identifiers were expanded by their tag. Common and unambiguous descriptors such as leaf or root were translated without adding a tag.

### The certainty

While this approach created a text that could easily be read by a machine, it was not suitable for human consumption, as it largely consisted of lists of tags. To remedy this, we first introduced hierarchical lemma tags merging inflected forms of one and the same word and spelling variants thereof into one tag. To use an English example, ‘apple’ in the singular, ‘apples’ in the plural, and a misspelled form ‘aplle’ would all be represented by one and the same tag. Here, plant lemmata were called JCLP and mineral lemmata JCLM, each followed by a current numbering. The other categories were not lemmatised.

In another step, each lemma tag was assigned a certainty factor ranging from 4 to 1. The highest certainty factor was used for items that were abundantly described, and often also in multiple source types, for instance wine, olives, water etc. These certainty factors were first created by BZ, the Classicist, using her personal knowledge that she acquired through working experience, dictionaries, and other primary sources, mostly on the TLG, and then checked by AL from his ethnopharmacological point of view to create a balanced result. We then translated all words that could confidently be translated.

In a final step, BZ fed translations of all plant and minerals names back into the text while keeping the lemma tag *in situ*, with the exception of items of which we had no reliable identification. We now have a readable text with tags that make it possible for a human to read the text and check the appendices for details on our confidence factor, the frequency of mentions, and sub-tags.

The full list of tags can be found at 8.

### Using the tagging system creatively

The resulting .txt file was then analysed in AntConc 4.0.5, ^9^ a freely accessible linguistic analysis software. In a second step, a copy of the file was disassembled into chapters, which resulted in a folder of 253 files named according to their current chapter numbering. Each of these chapters starts with a heading, for instance ‘on cough’, which is then followed by pharmaceutical prescriptions for the treatment of the respective disorder.

To facilitate the analysis, these chapters were then classified and tagged according to established standards for the ethnopharmacological analysis of our data. ^10^ This system was then adjusted for the philological work by adding two classifications; toxicology and trauma. Here, trauma refers to any externally caused injuries, for instance a fingernail that has been smashed by a stone, bedsores, or a small insect in the ear (chapters 25, 66, and 71). This category also covers chapters 24 and 217, which describe worm infestations of the ear and of wounds respectively. These could either be understood literally, as maggots or an insect, or as an early attempt to describe an infection. Toxicology was separated out for the philological analysis as it can form a distinct genre in ancient and medieval medicine.

AN – Andrology (10 chapters)

BS – Blood, spleen (16 chapters)

CV – Cardiovascular (0 chapters)

DE – Dermatology (25 chapters)

FV – Fevers (7 chapters)

GI – Gastrointestinal tract (36 chapters)

GY – Gynaecology (10 chapters)

ID – Infectious diseases (27 chapters)

LG – Liver and gall-bladder (11 chapters)

MA – Maternity (4 chapters)

MC – Mental conditions (2 chapters)

MN – Metabolic and nutritional disorders (8 chapters)

MS – Musculo-skeletal (7 chapters)

NC – Neurological conditions (20 chapters)

OP – Ophthalmology (28 chapters)

OC – Oral cavity (20 chapters)

OT – Otology (12 chapters)

RE – Respiratory tract (9 chapters)

RL – Rhino-Laryngology (13 chapters)

TR – Trauma (15 chapters)

TX – Toxicology (10 chapters)

UR – Urology (14 chapters)

XY – Residual category (19 chapters)

A file could also have multiple classifications, for instance chapter 96, which describes a headache caused by excessive wine intake, falls into both neurology and toxicology.

These simple measures make it possible to display two matters – where in JC specific materia medica is mentioned, and for which purposes it is used. It would have been possible to retrieve this information manually, but at significantly greater expense of time. Moreover, the software provides a clear picture that has been stripped of any unnecessary detail so that one does not lose view of the forest for the trees.

## Results

### Analysing apparent chaos

The new editorial conventions also had the rather unexpected effect that they brought out a highly developed medical and pharmaceutical practice that had insofar been hidden underneath a dialectal surface. However, the structure of JC may on occasion appear obscure. Roughly speaking, it falls into two head to foot sections, with the beginning of the text borrowed from an anonymous Byzantine manual. Larger parts of the remaining content draw on Paul of Aegina. ^11^

Our tagging system allows us to display clusters and patterns that are otherwise difficult to spot. In the following we list four distinctive findings to illustrate how helpful the use of AntConc can be compared with traditional manual analysis:

***Agriostaphida, staphis agria* - ἀγιοσταφίδα, σταφὶς ἀγρία (JCLP_003).** This plant is described in Dioscorides IV 152 (= II 296 f. Wellmann), who mentions a number of potential practical applications, lice infestation, itching, mange, oral infections, and tooth ache. Apart from mange, which could also refer to illnesses that have a similar external presentation but a different cause than our modern understanding, we can be confident that the Greek words describing these illnesses have been correctly translated.

Dioscorides also states that this plant is useful to expel phlegm. ^12^ Here, the term phlegm does not necessarily mean the same as our contemporary understanding. Phlegm in the ancient sense was one of the four humours, and it had the qualities cold and wet. To counteract it, a hot and dry medication would be indicated, or alternatively an agent that expels phlegm. Phlegm could be located everywhere in the body, and cause a person to be phlegmatic. It could equally be mucus in the modern sense.

In JC, *agriostafida/stafisagria* is indicated for the same diseases as in Dioscorides, with the curious addition that it is said to be useful to expel phlegm from the head, rather than phlegm as such, as stated in Dioscorides. With the exception of itch and *psora*, skin conditions without a defined place of manifestation, all mentions of *agriostafida/stafisagria* in JC are therefore located in chapters on the head.

This could just be a coincidence, since the vast majority of chapters in JC concern illnesses of the head:

Head: 93 chapters

Chest: 9 chapters

Stomach: 71 chapters

Urogenital: 38 chapters

Skeletal/muscular: 7 chapters

General/no set place of manifestation: 79 chapters

Uncategorised: 19 chapters

However, there could also be a deeper reason for this distribution. Therapeutic texts generally follow a head to foot order, *a capite ad calcem*; that is diseases were listed according to their main place of manifestation. A therapeutic text would generally start with hair loss or headache, and then move downwards until it reached the feet. JC has a complex history, with larger blocks of text drawing on earlier sources. A grouping like this could indicate an intermediary source which favoured this specific ingredient.

**Figure 1.**
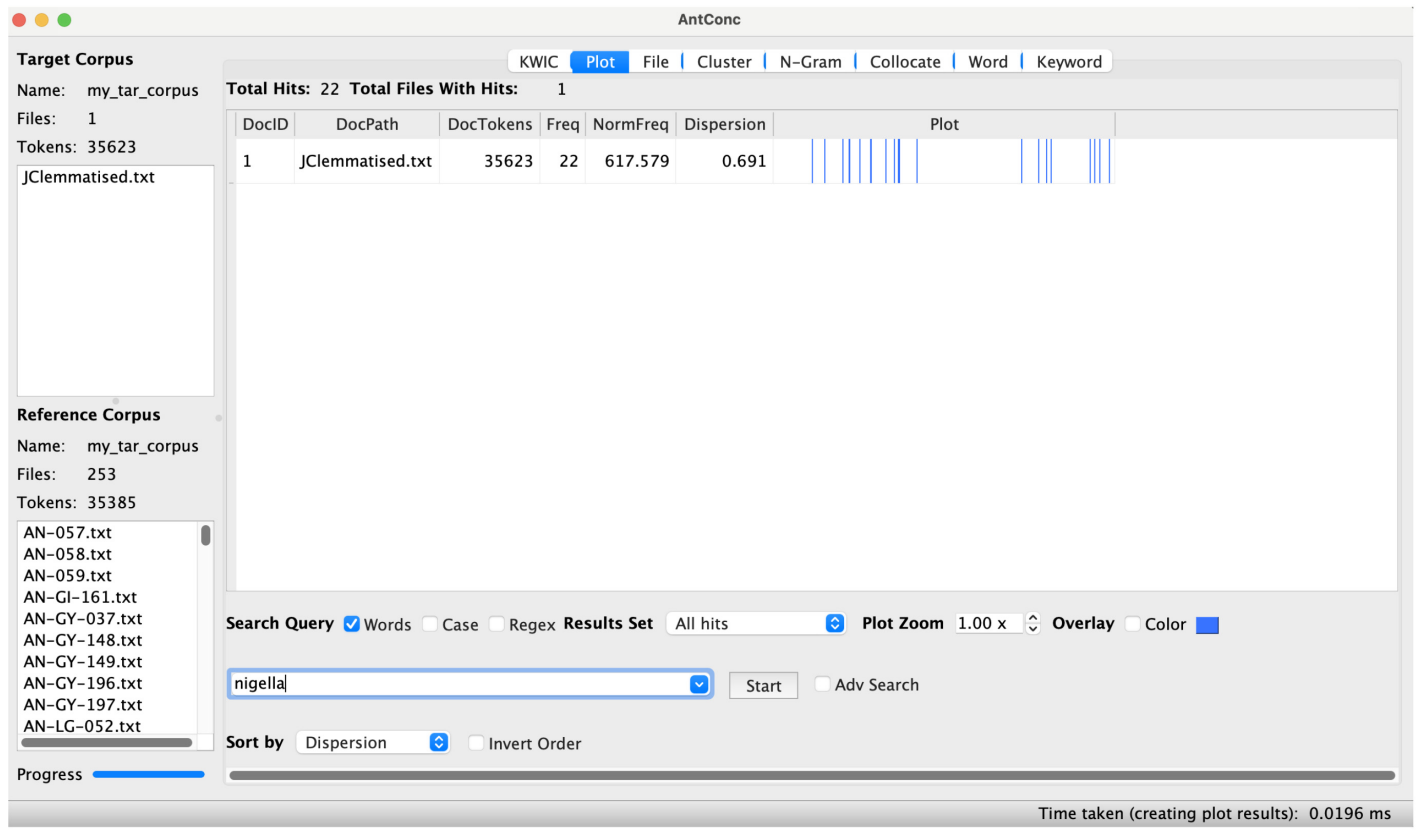
The mentions of *nigella* in JC, visualised in AntConc.

***Melanthin, melanthion* μελάνθιν, μελάνθιον (JCLP_126).** If one searches JC for mentions of this common pharmaceutical and culinary ingredient, an oddity comes to light – the plant name is not mentioned in a substantial block of chapters in the middle of JC ( Figure 1).

These chapters fall into a section of JC which tends to align with Theophanes Chrysobalantes and Paul of Aegina. Here, the headings are particularly stable, but the main text body of the chapter can vary significantly. ^13^ As above, this finding, which is particularly striking once visualised in AntConc, could help shed light on the history of the text.

***Ammoniakon* ἀμμωνιακόν (JCLP_015).** This plant product is described in Dioscordes III 84 (= II 100 Wellmann). Amongst several other uses, this work recommends *ammoniakon* as a treatment for an enlarged spleen, and as a treatment for rough eyelids and *leucoma*. This term derives from the word ‘white’, and while Dioscorides does not provide any further explanation, other medical authors describe it as a white patch on the cornea. ^14^ For these two ophthalmic applications, ^15^ Dioscorides specifies that *ammoniakon* has a localised method of action, directly on the surface.

The full list of possible applications according to Dioscorides is: warming, softening and dissolving indurations and growths, purging, inducing an abortion, reduction of the spleen, relieving of joint and hip pain, asthma, epilepsy and pulmonary edema and expelling blood stained urine. Apart from asthma, which could also refer to other breathing difficulties in the modern sense, we can be confident that these illnesses have been translated correctly.

**Figure 2.**
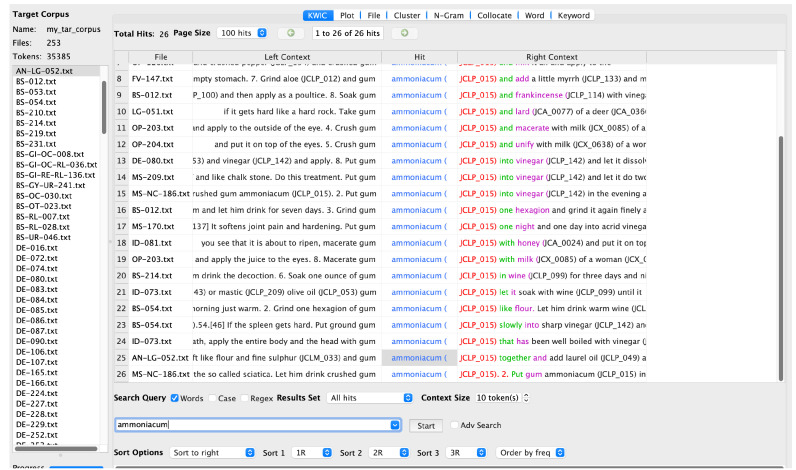
AntConc search results for *ammoniakon* with tagging.

In JC, similar uses are described. *Ammoniakon* is recommended as a treatment for an enlarged or hardened spleen and for a cloud in the eye, which could be identical or similar to the *leucoma* mentioned by Dioscorides. ( Figure 2). ^16^

The full list of practical applications is:

12. When the spleen is hardening.

51. For the liver if it gets hard like a hard rock.

52. On dropsy that is if the whole body swells and the belly and the feet and the testicles.

54. If the spleen gets hard.

73. On *kelephiasis*.

80. On lichen.

81. For *aporyphas*.

82. If the scar of a wound gets black.

120. *Amblyopia* is if he sometimes sees a little sometimes very very little and sometimes not at all.

135. For those who happen to have difficulty breathing and cannot take a breath and are mostly doing poorly when it is cold.

147. For the disease that is called fever disease if there is a pestilential disease that the locals call fever disease.

170. It softens joint pain and hardening.

175. Therapy for cough.

186. If a person is suffering from the so-called sciatica.

203. For a cloud of the eyes.

204. For pain of the eyes.

209. If the joints of the feet or hands get stiff and like chalk stone.

214. Therapy for those who are suffering from the spleen and are swollen.

To a modern reader, this list might seem rather idiosyncratic. From an ancient and medieval point of view, however, it is actually well defined. *Ammoniakon* primarily softens (12, 51, 54, 170, 186, 209, and 214) and removes superficial pathological changes (73, 80, 81, and 82). Chapter 203 might also fall into the latter category. Another clearly defined use is the treatment of respiratory problems (135 and 175). It is associated with the liver (51and 52), presumably due to its perceived action on splenic disorders (12, 54, and 214). This pattern does not fully agree with the practical application mentioned by Dioscorides. Here, in particular pestilential disease and *amblyopia* do not seem to fit. For the former, an association with skin manifestations may have been the reason for inclusion. As for *amblyopia*, no real connection to Dioscorides’ description can be found. In our modern understanding, amblyopia occurs when the brain decides to use primarily vision from one eye. Mostly, this is brought on by pathological changes. It is often reversible with treatment, but not intermittent as described in JC. ^17^ The term itself translates to ‘blunted vision’. Throughout ancient and medieval literature, the term *amblyopia* is used frequently to describe an unspecific visual impairment. ^18^ The description provided by JC and the corresponding chapter in JP, its direct source, is to our knowledge unique. As far as the treatment of *amblyopia* is concerned, both JP and JC recommend blood letting near the inner corner of the eye along with medicinal treatment. ^19^ Here, JP offers fewer treatment options, with no mention of *ammoniakon*. Localised blood letting is also described in the corresponding passage of Paul of Aegina, ^20^ as is *ammoniakon*. Curiously, Paul describes evacuation of blood through leeches or an incision as well as sneezing and vomiting, which puts pressure on the blood vessels in the eye. Blood letting close to an actual or perceived location of a disease is very common in ancient and medieval health care. The circulation of the blood was yet to be discovered, and therapy was based on the four humour theory. An abundance of one humour could cause pathological changes, which left two choices – changing it through medication or another external intervention or evacuating it through an incision. But was this always a misguided belief? It is certainly striking to see that one and the same treatment is indicated for splenic disorders as well as visual disturbances that are also cured by localised blood letting. This raises the question whether there is a connection between these two, for instance a haemoglobin disorder affecting both the spleen and the eyes, and in particular since some Mediterranean islands as well as southern Turkey are prone to these disorders. ^21^

**‘JCLP_167 that is JCLP_074’** This is a quotation from chapter 12.7, which also occurs more or less in this form in 24.6, 42.9, 64.4, and 152.3, and it presents a non sequitur – our tagging system or translation seems to have failed. Our plant lemma 167 ποταμογείτων *potamogeiton*, which translates to ‘neighbour of the river’, is equated with plant lemma 074 καλαμίνθη *kalaminthe*. There are common beliefs about these two plant names, but these do not refer to the same species.

The phrase ‘plant X that is plant Y’ is often found in late or post Byzantine vernacular pharmacy texts. The intention here is to provide the reader with valuable additional information in order to make sure that the correct medicinal plant is used in the recipe. Either the vernacular plant name is equated with the learned name of the respective plant or two vernacular synonyms are equated. That the association in our example also makes sense from a botanical perspective is supported by information in Gennadios: ^22^ For the learned name άγριος ηδύοσμος *agrios hedyosmos* the vernacular names αγριόδυοσμος *agriodyosmos* or καλαμίθρα *kalamithra* are provided, which in Cyprus, as stated, corresponded to ποταμογείτανος *potamogeitanos*. Assuming that καλαμίθρα *kalamithra* in this case is a synonym of καλαμίνθη *kalaminthe*, we here can find the same association. Gennadios identifies all these names with the botanical taxa *Mentha longifolia* and *M. sylvestris*, the latter, according to Kew’s Medicinal Plant Names Services, ^23^ being a synonym of *M. longifolia* (L.) L. ssp. *longifolia* and thus a subspecies of the former. This identification is also supported by ethnobotanical field studies on Cyprus which recorded the names *potamoyitanos* and *agriodhyosmos* for the locally endemic subspecies *Mentha longifolia* (L.) L. ssp. *cyprica* (H. Braun) R. Harley, ^24^ a plant which typically grows along water-courses. On the other hand, the same names, *potamoyitanos* and *agriodhyosmos*, as well as the name *kalamithra* were also recorded for *Mentha aquatica* L. on Cyprus, a closely related species of the same genus and with the same preferences regarding its habitat. Whatever the botanical species associated, the plant names *potamogeiton* and *kalamintha* indeed may refer to one and the same plant, as purported by our text.

### Some philological notes

JC is a highly unusual text. One the one hand, it follows literary convention. After all, it is a therapeutic handbook with a commentary. On the other hand, the style of the text is neither literary nor polished. Rather, it is reminiscent of annotations on fly leaves or blank pages, which are commonly found on medieval manuscripts. This is an advantage as the author describes a lot of detail that a medieval academic would not, and at a disadvantage as it poses problems for our translation. Since we cannot improve the text beyond its reconstructed original, this only leaves us one option, to translate the text as it is – at the danger of sounding like bad English.

The preface of JC is not translated here, as this text is very corrupt.

Our work does not aim to provide a dictionary that can universally be applied to all ancient and medieval medical texts. While we acknowledge that Dioscorides’ work was seminal in this field, we are well aware of the fact that a given plant name may have had referred to an entirely different species in another geographic location or at another point in time.

## Conclusion

Our methodology performs two major tasks, it makes our primary source readable for a human, for instance for scientists with an interest in medicinal herbs or for philologists and medical historians from the field of Islamic studies who are not proficient in medieval vernacular Greek dialects. Moreover, it facilitates the use of specialist software, which reveals connections that are otherwise obscure. The use of identifiers and confidence factors enables the reader to form conclusions on how reliable the translations of materia medica are. The rise of artificial intelligence could pave the way for complex analysis that is at present too labour intensive for a conventional research project. Here, new connections could come to light. For instance, we do not fully understand the role of blood letting, and some dietary instructions could be motivated by minerals or vitamins contained in certain food or its influence on the microbiome.

## Translation of JC

1.[1] For acute headache. Grind dry ivy (JCLP_087) or also fresh one and soak in olive oil (JCLP_053). Sift it with a cloth and rub it on the forehead and the temples. That is take ivy (JCLP_087) and dry it in the sun. Then grind it and make it like flour. And again put fresh ivy (JCLP_087) into olive oil (JCLP_053) one day and one night. And let it soak, just the leaves. Then throw out the leaves and take the flour of the other, dry ivy (JCLP_087) and mix it with the olive oil (JCLP_053) of the fresh ivy (JCLP_087). And rub the olive oil (JCLP_053) on the forehead and the temples that is the *melingas*. 2. Burn a bone of a deer (JCA_0366), soften it with rose (JCLP_181) olive oil (JCLP_053) and apply. Or once you have burnt the bone of a deer (JCA_0366), grind it and make it like flour. And then mix it with rose oil (JCLP_181) and apply to the forehead and the temples.

2.[2] For headache and migraine. Knead cress (JCLP_077) with vinegar (JCLP_142) and with rose (JCLP_181) olive oil (JCLP_053) and make a thick wax plaster and apply. That is take cress (JCLP_077) and grind it and make it like flour. And mix it with vinegar (JCLP_142) and with rose oil (JCLP_181) and make it like a dough that is not too solid. Then apply to the head and one hemisphere of the head. 2. Grind JCLP_198 and frankincense (JCLP_114) and take it and do the same. That is first grind the frankincense (JCLP_114) and make it very smooth, then JCLP_198 and mix it with vinegar (JCLP_142) and make it like thin glue and apply to the head and the forehead and the temples.

3.[3] For heat of the head. Take rose oil (JCLP_181) and vinegar (JCLP_142) and warm it up. Then apply to the whole of the head. 2. Heat is called when someone thinks that the head is on fire and burning. But first let his blood from the vein on the head. Then apply also warm vinegar (JCLP_142) and rose oil (JCLP_181). And he shall not drink wine (JCLP_099) and the sun shall not hit him nor wind nor smoke. He shall not eat sharp things nor warm things and let him also be treated with a clyster.

4.[5] For the eyes. Apply ground JCX_1217 and he will be healed. Or burn the hooves of a male donkey (JCA_2298), mix it with the milk (JCX_0085) of a woman (JCX_0118) of the mother of a male child and apply. 2. Boil egg (JCA_0630), grind the yolk (JCX_0114) without the white (JCX_0101) and another yolk (JCX_0114) which they take to eat. Grind it all together, then grind JCLP_078 and mix the juice with yolk (JCX_0114) of an egg (JCA_2898) and with the other JCA_2086. And add a little bit of syrup and rose water (JCLP_181) and mix it all and soak cotton (JCLP_029) in the juice. And put it onto his eyes. And let his blood. And he shall not drink wine (JCLP_099). And let him abstain also from the other things that I mentioned when there is a heat of the head. Make the same treatment also for a wound where there is an inflammation like a hearth and the place is on fire.

5.[4] When the eyes are itching. Take the bark of a sour pomegranate (JCLP_182) and roast it in the sun and grind. And make it like four. And take a little wine (JCLP_099) and stir it. Then apply to the eyes and on top put a cloth (JCLP_151) soaked with wine (JCLP_099) and bind it until the morning. And let his blood and wash him and let him abstain from any sharp things, and from wine (JCLP_099) and the sun and smoke and the wind.

6.[5] For flux from the eyes. Take frankincense (JCLP_114), mastic (JCLP_125), myrrh (JCLP_133) and grind it one by one. And five beans (JCLP_218) and also make these into flour. Then take the white (JCX_0101) of an egg (JCA_0105) and add what you have ground and mix it and take it and apply to the forehead. And on top put hemp (JCLP_205).

7.[6] When blood is flowing from the nose. Burn the shell (JCX_1360) of an egg (JCA_0105) and grind it well and put it into a reed. And put one part of the reed into his nose and blow into the other part and it will get into the nose. 2. Grind mustard (JCLP_192) and a few nuts (JCLP_079) one after the other and then mix them. And the white (JCX_0101) of an egg (JCA_0105) and put it onto the forehead. And the bleeding with stop. Fish (JCA_2886) and aloe (JCLP_012) grind likewise and smooth, apply and it will stop.

8.[7] When he spits blood. Grind mint (JCLP_063) and mix its juice with a little vinegar (JCLP_142). And let him drink.

9. [8] For pain of the tongue. Chew the leaves of an olive tree (JCLP_053). And keep it on your tongue for a long time.

10.[9] When the bowels are hardening. Put bread (JCLP_024) crumbs into a pan. And let it boil with water (JCLM_022). And then add honey (JCA_0024) and make it smooth. And spread it out into cloth (JCLP_151) and apply. Grind linseed (JCLP_116) well and barley flour (JCLP_100). And boil it with water (JCLM_022). Then add syrup (JCO_0157) and make it smooth. And spread it out into cloth (JCLP_151) and apply. Do not do this also when a hardening of tumours is found.

11. [10] For liver pain. Grind three celery (JCLP_186) roots and garlic (JCLP_197) together. And let it boil with good wine (JCLP_099) and let him drink in the morning. And do the same for those suffering from dysuria. If he has fever, boil it with water (JCLM_022).

12. [11] When the spleen is hardening. Take leaves of cabbage (JCLP_098) and remove their veins and grind the leaves on their own and then add ground yellow orpiment (JCLM_006) and ground frankincense (JCLP_114). First, let the leaves boil with water (JCLM_022) in a pan. Add what you have ground and mix and make it smooth and put it for three days and then remove the poultice and add a little vinegar (JCLP_142) and let him swallow. 2. Strip willow (JCLP_072) bark close to the root. And boil it with good wine (JCLP_099) and let it boil once. And then again add wine (JCLP_099) and let it boil again and add it up to three times and give him and let him drink for seven days. 3. Grind gum ammoniacum (JCLP_015) one *hexagion* and grind it again finely and add wine (JCLP_099) into the gum ammoniacum (JCLP_015) and let it soak. And let him drink with wine (JCLP_099). 4. Grind gum ammoniacum (JCLP_015) and mix it with olive oil (JCLP_053) and crush the bark of capers (JCLP_076) and mix in gum ammoniacum (JCLP_015) and make kernels like those of a chickpea (JCLP_055). And let him swallow those at night that is at dinner time. And he shall not have dinner. Five kernels at dinner time and when he has swallowed those let him lie down on the part of the body where the spleen is. 5. Boil barley flour (JCLP_100) with vinegar (JCLP_142). And make it like glue and apply it. 6. Grind the root of capers (JCLP_076) and cold lime (JCLM_007) and make it smooth with vinegar (JCLP_142). And sprinkle it onto the spleen for seven hours. 7. Add sharp vinegar (JCLP_142) and the leaves of JCLP_167 that is calamint (JCLP_074) and water (JCLM_022) and let it boil. And then add barley flour (JCLP_100), then apply as a poultice. Also the juice of these plants and let boil barley flour (JCLP_100) and then apply as a poultice. 8. Soak gum ammoniacum (JCLP_015) and frankincense (JCLP_114) with vinegar (JCLP_142). Then grind and make it like glue. Cover over the area of the spleen and put hemp (JCLP_205) on top.

13. [12] Hot poultice if the kidneys hurt. Infuse bran (JCLP_160) and pennyroyal (JCLP_040) in hot water and strain through a cloth. Soak a sponge (JCA_0923) and lay on the body part, just hot. But beforehand administer a clyster.

14.[13] For anasarca. Grind dry pine resin (JCLP_177) and make it like flour. And spread on the lesion.

15.[14] For clogged up ears. Put syrup into the shell (JCX_0167) of an egg (JCA_0105) and put the shell (JCX_0167) on the coals and let it become warm. Then put it into the ear. 2. Grind cinnamon (JCLP_086) and make it like flour. Then take hemp (JCLP_205) and spread it out. Then pour yolk (JCX_0114) of an egg (JCA_0105) onto hemp (JCLP_205) and on top of the egg (JCA_0105) the ground cinnamon (JCLP_086) and lay it onto the ear like this.

16.[15] On pityriasis of the head. It is advised to purge the entire body either through blood letting or through ingestion of medication. Then grind the leaves of beetroot (JCLP_189) and anoint its juice on the head. Or grind frankincense (JCLP_114) and mix it with wine (JCLP_144) and with olive oil (JCLP_053) and anoint the head. 2. Grind litharge (JCLM_018) and pepper (JCLP_154) and frankincense (JCLP_114) one after the other. Then add olive oil (JCLP_053) and mix it and anoint the head. 3. Put water (JCLM_036) into a pot and wild cucumber (JCLP_001) in small pieces and grind lupine (JCLP_118) and mix and let boil. And wash the head with their juice.

17. For headache. If the forehead is hot, grind purslane (JCLP_041) and mix with barley flour (JCLP_100) and with honey (JCA_0024) and anoint the forehead and on top put hemp (JCLP_205) and the leaves of beetroot (JCLP_189) with barley flour (JCLP_100) and honey (JCA_0024) and yolk (JCX_0114) of an egg (JCA_0105) or the leaves of bramble (JCLP_032) or of JCLP_174 and mix these and also with barley flour (JCLP_100) and honey (JCA_0024) and JCLP_078 and put these on the forehead, just grind the leaves before and the JCLP_078 and then mix in the barley flour (JCLP_100) and the honey (JCA_0024) and the yolk (JCX_0114) of an egg (JCA_0105).

18.[16] For pain in the eyes. Blood letting is advised for these and after that put white (JCX_0101) of an egg (JCA_0105) into the eye or milk (JCX_0085) of a woman (JCX_0118). Also put chamomile (JCLP_227) and fenugreek (JCLP_212) and roses (JCLP_181) and water (JCLM_022) into a pot and let it boil and water (JCLM_022), this one warm. Apply a hot poultice of the eyes with cloth (JCLP_151).

19. For flux from the eyes. Take the flour of cereal (JCLP_194) and add ground frankincense (JCLP_114) and JCLP_240 and white (JCX_0101) of an egg (JCA_0105) and make these soft and then spread it out into a cloth (JCLP_151) and put it on the forehead. 2. Take wheat flour (JCLP_187) and add crushed frankincense (JCLP_114) and myrrh (JCLP_133) and white (JCX_0101) of an egg (JCA_0105) and mix these and apply these evenly on the forehead with cloth (JCLP_151). 3. Remove the flesh of a snail (JCA_0678) of the sea or land. And mix ground frankincense (JCLP_114) and grind it and spread it out like this on a cloth (JCLP_151) and put it on the forehead. 4. Make crushed frankincense (JCLP_114) and JCLP_240 soft with vinegar (JCLP_142) and apply to the forehead.

20. For pain of the eyes. Boil fleawort (JCLP_235) that is called fleawort (JCLP_188) and put JCX_0404 into its broth and rose water (JCLP_181) and apply to the forehead. 2. Take JCLP_204 which some call JCLP_038, when its leaves are small and a bit black. Grind its kernels or its leaves as you prefer and put ground salt (JCLM_002) into their juice and anoint the forehead. 3. Put astragalus (JCLP_210), in the evening, into water (JCLM_022) so that it is all soaked. And in the morning sift it through a cloth, then add fleawort (JCLP_188) and rose water (JCLP_181) and anoint the forehead.

21.[17] For bloodshot eyes. Bloodshot eye is if there is something like a red lentil (JCLP_219) on the pupil or the white of the eye. For these it is advised to drip the blood of a pigeon (JCA_0882) into the eye or of a wood pigeon (JCA_2792) or ground Cappadocian salt (JCLM_002) or milk (JCX_0085) of a woman (JCX_0118), while she expels it warm. And boil hyssop (JCLP_216) and apply a hot poultice of the juice on the eyes if both eyes have such a red matter. If just one has it, apply a hot poultice on this one eye.

22.[18] When pus is flowing from the ears. Take warm wine (JCLP_144) and honey (JCA_0024) and mix them. Then take a clyster but not the one that they are using to treat the anus but the one that the doctors have which is called ear clyster. And do not put the reed inside but hold it outside opposite of the opening and compress the little pouch so that the juice comes out, and do not be afraid. For the JCX_2287 will come out again and you will only wash the pus out of the ear. 2. Add rust (JCLM_028) of iron (JCLM_027) and vinegar (JCLP_142) and let it react for a week and then drip it into the ear inside. 3. Grind aloe (JCLP_012) and mix it with wine (JCLP_099) and drip it into the ear. 4. Grind the leaves of an olive tree (JCLP_053) and mix the juice with honey (JCA_0024), and drip a little into the ear. 5. Make cedar oil (JCLP_083) soft with honey (JCA_0024) and drip it into the ear. 6. Crush aloe (JCLP_012) on its own and pour it into the ear, just a little without fluid. 7. The decoction of antimony (JCLM_029) that is its juice once it has boiled with honey (JCA_0024) and with wine (JCLP_099) without water (JCLM_022). Let it get warm and drip it into the ear. 8. Mix wine (JCLP_144) and honey (JCA_0024) both warm and drip into the ear. 9. Boil lentils (JCLP_219) with water (JCLM_022) and drip the juice into the ear. 10. Boil roses (JCLP_181) with water (JCLM_022) and drip the juice into the ear.

23.[19] For the ears, when they are bleeding. Drip the juice of leek (JCLP_169) into the ear. Crush JCX_0607 wine (JCLP_144) and mix it with warm wine (JCLP_099) and drip it into the ear.

24.[20] For worms that are in the ears. Grind the leaves of capers (JCLP_076) and drip the juice into the ear. 2. Drip urine (JCX_0876) of a person into the ear, warm as soon as he urinates. 3. Crush white JCU_0278 and grind it and mix it with wine (JCLP_099) and drip it into the ear. 4. Mix cedar oil (JCLP_083) with vinegar (JCLP_142) and drip it into the ear. 5. Drip the juice of leek (JCLP_169) into the ear. 6. Grind fresh JCLP_167 that is calamint (JCLP_074), and drip its juice into the ear. 7. Grind the leaves of a nectarine (JCLP_179) and drip the juice into the ear.

25.[21] When something gets into the ear, or a flea. When a flea gets in, put olive oil (JCLP_053) into the ear, just warm. 2. When something else gets in, put the tips of the hair of his head into the ear, just rolled up hair, as much as fits into the ear.

26.[23] When there is a sound in the ear that is *ankhonen*. Boil cedar (JCLP_083) with vinegar (JCLP_142) and drip it warm into the ear. 2. Grind the tender shoots of bramble (JCLP_032) and mix the juice with honey (JCA_0024) and stir it and drip it into the ear, just warm. 3. Mix butter (JCA_0330) with honey (JCA_0024), and warm it up and drip it into the ear. 4. Drip dissolved unsalted lard (JCA_0077) of a goose (JCA_0246) into the ear. 5. Boil wormwood (JCLP_028) and drip the warm decoction into the ear. 6. Take cumin (JCLP_105) and rue (JCLP_157) and vinegar (JCLP_142) and rose oil (JCLP_181) and let it boil and drip it like this warm. 7. If someone cannot hear much nor is completely deaf, it is advised to drink JCX_0272, then also the JCX_0498. 8. Drip the urine (JCA_2332) of a goat (JCA_0478) or the bile. 9. Grind the leaves of rue (JCLP_157) and mix the juice with honey (JCA_0024) and drip it into the ears. 10. Drip warm vinegar (JCLP_142) and JCX_2524 into the ear. 11. Warm up vinegar (JCLP_142) and honey (JCA_0024) and drip it into the ear. 12. Mix ground castor (JCA_0145) with dill oil (JCLP_073) and drip it in warm. 13. Grind seven or five sowthistles (JCLP_062) and boil the juice with olive oil (JCLP_053). And drip it warm into the ear as much fits in. 14. Mix the juice of a radish (JCLP_176) with rose oil (JCLP_181) and drip it warm into the ears. 15. Unify crushed castor (JCA_0145), crushed white JCU_0278 and vinegar (JCLP_142) and rose oil (JCLP_181) and mix it all warm and drip it into the ear. 16. Warm up both the bile of a goose (JCA_0543) and table oil (JCLP_232) in equal amounts and drip it into the ear. 17. Grind the shoots of leek (JCLP_169) and drip the warm juice into the ear. 18. Let onion (JCLP_102) and hyssop (JCLP_216) boil with urine (JCX_2332) of a person. And drip it warm into the ear. 19. Drip the bile of a hare (JCA_0453) and milk (JCX_0085) of a woman (JCX_0118) into the ears, just warm. 20. Mix the bile of a bull (JCA_0466) with the juice of leek (JCLP_169) and drip it into the ear. 21. Grind the seed of laurel (JCLP_049) that is its kernels and mix them with old wine (JCLP_144) and warm it up and drip it into the ear. 22. Warm up the bile of a goat (JCA_0965) and the juice of onions (JCLP_102) and drip it into the ear. 23. Let antimony (JCLM_029) and wine (JCLP_099) and honey (JCA_0024) boil and once they are warm, drip them into the ear.

27.[24] For the parotids, that is the swelling below the ear that looks like abscesses. Boil barley flour (JCLP_100) and the flour of linseed (JCLP_116) and fenugreek (JCLP_212) with honey (JCA_0172) and wine (JCLP_099) and put it on top. 2. Grind the leaves of monk’s rhubarb (JCLP_111) with unsalted lard (JCA_0753). Unify ground salt (JCLM_002) and leek (JCLP_169) and put it all on top of the area. 3. Boil sea water (JCLM_036) and put it on warm with wine (JCLP_144). And warm up salt (JCLM_002) and put it on warm. 4. Apply a poultice of flour of lupine (JCLP_118) and syrup (JCO_0157) and boiled and crushed JCLP_036.

28.[25] For a lot of blood when it flows out of the nose. Dry the leaves of calamint (JCLP_074) and grind them and blow them into the nose through a reed. 2. Soak a new sponge (JCA_0200) in cedar oil (JCLP_083). And then burn the sponge (JCA_0923) well so that it roasts. Then make it like flour. Blow it through a reed (JCLP_075) into the nose. 3. If the right nostril bleeds a lot excessively, bind the right hand and feet day and night and the right side, that is the liver. Apply cupping that is cup the area but without injuring it. If blood is flowing from the left side, again do binding and cupping on the left side.

29.[26] To make the teeth white. Burn horn (JCA_0502) of a deer (JCA_0366) until it gets white. Then grind it and make it like flour and apply to the teeth. 2. Apply ground salt (JCLM_002) and mastic (JCLP_125) and rub on the teeth. 3. Roast barley (JCLP_100) and grind salt (JCLM_002) and rub it onto the teeth. 4. Unify the seed of fennel (JCLP_123) and the flowers of roses (JCLP_213) and crush them and apply it to the teeth. 5. Grind salt (JCLM_002) and pepper (JCLP_154) and henna (JCLP_107) and crush it and apply it to the teeth. 6. Unify pumice (JCLM_016) with dry roses (JCLP_213) and crush, and apply it to the teeth.

30.[27] For blood when it flows into the gums. Boil the leaves of an olive tree (JCLP_053) with water (JCLM_022) and keep this decoction in the mouth for a long time. 2. Take the shoots of myrtle (JCLP_134) and the flower of roses (JCLP_213) and vinegar (JCLP_142) and wine (JCLP_099) and let it boil and keep the decoction in the mouth. 3. Let the tips of JCLP_113 and the root of a quince tree (JCLP_103) and vinegar (JCLP_142) and wine (JCLP_099) boil and keep the decoction in the part of the gums where it hurts, just for a long time. 4. Let the tips of mastic (JCLP_209) and the root of a quince tree (JCLP_103) and vinegar (JCLP_142) and wine (JCLP_099) boil and keep the decoction in the mouth.

31.[28] For rotten gums. Make ink-gall (JCLP_085) like flour and dry myrrh (JCLP_133) like flour and sprinkle it onto the gums. 2. Grind dry JCX_0114 and make salt (JCLM_002) like flour with honey (JCA_0024) and mix and apply. 3. Grind dry JCX_0114 and salt (JCLM_002) and make it like flour and mix it with honey (JCA_0024) and apply to the gums.

32.[29] For split lips. The stone of a date (JCLP_222) has something like a membrane. Take this and glue it to the area where the lips are split. 2. Apply lard (JCA_0077) of a goat (JCA_0966) to the lips. 3. Apply lard (JCA_0077) of a goose (JCA_0246) to the lips. 4. Apply the marrow of cattle (JCA_0769) to the lips. 5. Apply the marrow of a deer (JCA_1760) to the lips. The shell (JCX_0167) of an egg (JCA_0105) has a white membrane. Take this and glue it to the lips. 6. Apply the lard of male calf (JCA_1215) to the lips. 7. Boil the fresh leaves of roses (JCLP_213) in wine (JCLP_099) and glue one leaf after the other to the lips. 8. Make ink-gall (JCLP_085) and mastic (JCLP_125) like flour and sprinkle it onto the lips. 9. Mix crushed mastic (JCLP_125) with wine (JCLP_099) and apply to the lips. 10. Burn the shell of sea molluscs (JCA_1245) and make it like flour and sprinkle it onto the lips.

33.[30] For the tongue if it is swollen. Let him chew purslane (JCLP_041). 2. Let him chew the leaves of JCLP_150. 3. Let him chew the tender leaves of an olive tree (JCLP_053). 4. Grind the tender leaves of an olive tree (JCLP_053) and mix them with olive oil (JCLP_053) and apply to the tongue. 5. Mix syrup (JCO_0157) with rose water (JCLP_181) and keep it in the mouth for a long time. 6. Mix fleawort (JCLP_235) with rose water (JCLP_181) and keep the juice in the mouth. 7. Keep JCLM_010 in the mouth.

34.[31] When the mouth smells. Burn dry roses (JCLP_181) and make them like flour and rub it on the teeth. 2. Boil stachys (JCLP_201) with wine (JCLP_099) and keep it in the mouth. 3. Chew stachys (JCLP_201) in the morning. 4. Rub the flower of mastic (JCLP_209) onto the gums. 5. Make pumice (JCLM_016) like flour, mix it with honey (JCA_0024) and apply to the teeth and the gums.

35.[32] On tonsillitis. Let his blood immediately. But don’t remove a lot of blood in one go and he loses consciousness and he dies. If not a bit, again also .... how many days you have let his blood, either if he has fever or if he does not have fever. And he shall not drink wine (JCLP_144) and also rinse his feet. And let him be hot and wind shall not hit him. If it is not useful, let his blood from underneath the tongue or on top of the tongue. Chop it with a razor. And administer a clyster every day for his belly and put boiled hair (JCA_0256) onto his neck with warm olive oil (JCLP_053). 2. Ground barley flour (JCLP_100) and linseed (JCLP_116) and ground dates (JCLP_222) and make a poultice from bread crumbs. And put it onto the neck. 3. Let dates (JCLP_222) boil with water (JCLM_022) and lentils (JCLP_219) and keep the broth in the mouth and gargle it. 4. Keep sweet milk (JCX_0085) in the mouth, just warm. 5. Let JCLP_096 and the flowers of roses (JCLP_213), honey (JCA_0024) and wine (JCLP_099) boil. 6. Keep the juice of bran (JCLP_160) in the mouth. 7. Dry the dung (JCA_0254) of a white dog (JCA_0920) and crush it and mix it with honey (JCA_0024) and a little vinegar (JCLP_142). And apply to the neck. 8. Burn the dung of wild swallows (JCA_2852). And mix their ashes (JCX_0126) with honey (JCA_0024). And apply it to the root of the tongue. 9. Grind the green leaves of rue (JCLP_157) and mix their juice with milk (JCX_0085) and apply it to the root of the tongue. 10. Boil nigella (JCLP_126) with honey (JCA_0024) and wine (JCLP_099) and apply it to the root of the tongue.

36.[33] If he spits blood. Let his blood both from the feet and the hands and apply olive oil (JCLP_053). Let his diet be austere. 2. Soak unwashed hair (JCA_0256) in warm vinegar (JCLP_142) and rose oil (JCLP_181) and apply a warm poultice to the chest. 2. Sour pomegranate (JCLP_182), apples (JCLP_131), JCLP_010 and sour pears (JCLP_021). 4. Grind the leaves of bramble (JCLP_032) and mix the juice with crushed lump of earth (JCLM_009) and let him drink it. 5. Give him also theriac (JCX_0301). It needs to be recently burnt, just with with a little wine (JCLP_099). 6. And apply a warm poultice on the chest with sponge (JCA_0200), for this purpose boil vinegar (JCLP_142) and wine (JCLP_099). 7. Soak unwashed hair (JCA_0256) in warm vinegar (JCLP_142) and rose oil (JCLP_181) and apply a warm poultice to the chest. 8. Also put the plaster which is called Of Willow (JCLP_072) onto the chest.

37.[34] For inflammation of the breasts. Take vinegar (JCLP_142) and wine (JCLP_099) and let it boil. Then take new sponge (JCA_0200) and soak it there well. Then wring it out well and put it on top of the breasts. And bind it and then again the same the next day. 2. Boil the crumbs of bread (JCLP_024) with vinegar (JCLP_142) and wine (JCLP_099). Then add ground dates (JCLP_222) to the crumbs and macerate well. Then spread them into a cloth (JCLP_151) and put them on top of the breasts. 3. Apply a poultice of crumbs on top with fleawort (JCLP_235). 4. Let crumbs boil with vinegar (JCLP_142) and wine (JCLP_099), then add ground purslane (JCLP_041) and make it smooth thoroughly and put it on top. 5. Boil the flour of barley flour (JCLP_100) or fenugreek (JCLP_212) with water (JCLM_022). And then add honey (JCA_0024) and macerate and put it on top.

38.[35] If he vomits when he eats. Ground aloe (JCLP_012), mastic (JCLP_125), storax (JCLP_206), gum ladanum (JCLP_110), frankincense (JCLP_114), the seed of wormwood (JCLP_028), syrup (JCO_0157), a little very little clean flour of cereal (JCLP_193) and a little vine flower (JCLP_143) and make a plaster and put it onto his chest. Mix the juice of mint (JCLP_063) and the juice of pomegranate (JCLP_182) and honey (JCA_0024) and let it boil and let it boil so that it gets very thick. And let him eat slowly when he is about to go to sleep and he shall not have dinner. Treat him also with a clyster.

39.[36] For dysentery. Let him drink ground Lemnian sealed medication (JCLM_032) with warm water (JCLM_022). 2. You would want to administer warm water (JCLM_022). Add into it crushed Lemnian sealed medication (JCLM_032) and pierce him with a clyster just without salt (JCLM_002) and olive oil (JCLP_053) and honey (JCA_0172). 3. Let bramble (JCLP_032) and equisetum (JCLP_069) that is called equisetum (JCLP_163), boil with wine (JCLP_099) and let him drink. 4. Dry unripe fruit of a mulberry tree (JCLP_208) and make it like flour and let him drink with warm water (JCLM_022). 5. Treat with a clyster, that is take rice (JCLP_147) and let it boil with water (JCLM_022) then take dissolved warm lard of a goat (JCA_1402) and put it into the juice of rice (JCLP_147) and treat him just without salt (JCLM_002) and honey (JCA_0172) and olive oil (JCLP_053). 6. Dry the unripe fruit of bramble (JCLP_032) and make it like flour and drink it with warm water (JCLM_022). 7. Put warm crushed lead white (JCLM_042) into water (JCLM_022) and JCX_0404 and stir it and administer as a clyster without olive oil (JCLP_053), honey (JCA_0172) and salt (JCLM_002). 8. Let him also drink theriac (JCX_0301) and let it be administered as a clyster with theriac (JCX_0301), that is warm water (JCLM_022) with theriac (JCX_0301). Let him stir it with water (JCLM_022) and let it be administered without honey (JCA_0172), salt (JCLM_002) and olive oil (JCLP_053). 9. Take the peel of myrtle (JCLP_134), of pomegranate (JCLP_182) the leaves of bramble (JCLP_032), wormwood (JCLP_028) and wine (JCLP_099) and let it boil. Then soak a new sponge (JCA_0200) in wine (JCLP_099), this being warm. And apply as a warm poultice to the anus. 10. Make a plaster and add two *hexagia* aloe (JCLP_012) two *hexagia* frankincense (JCLP_114) and syrup (JCO_0157).

40. [36] For cramps. Treat these with an enema that is put warm wine (JCLP_099) and honey (JCA_0024) and rue oil (JCLP_157) and ground lump of earth (JCLM_009) without salt (JCLM_002) and mix it all and administer. 2. The leaves of bramble (JCLP_032), the leaves of myrtle (JCLP_134), the leaves of mastic (JCLP_209), the leaves of laurel (JCLP_049), the peel of pomegranate (JCLP_182), crushed ink-gall (JCLP_085). Boil it all with water (JCLM_022). Then take a new sponge (JCA_0200) and soak it there in the decoction, and apply as a warm poultice to his anus and let it be hot. He shall not drink anything cold, the wind shall not hit him. He shall not be subject to blood letting.

41.[37] For a disposition to colic. Treat these with a clyster containing rue oil (JCLP_157) and crushed castor (JCA_0145) without salt (JCLM_002), honey (JCA_0172) and olive oil (JCLP_053). 2. Add crushed cumin (JCLP_105), the leaves of rue (JCLP_157), the lard of a goose (JCA_0543) or of a bird (JCA_0346) into table oil (JCLP_232) and let it boil and administer this oil (JCLP_053) with a clyster. Put a lot into a vessel with water (JCLM_022), not very warm, and table oil (JCLP_232) and rub it onto the patient. He shall abstain from warmth and shall rest. Put wormwood (JCLP_028) into water (JCLM_022) and let it boil and let him drink the warm decoction daily in the morning. 4. Let him drink ground castor (JCA_0145) with warm water (JCLM_022) in the morning. Take a little crushed pepper (JCLP_154) and a little vinegar (JCLP_142) and a little honey (JCA_0024) and let him drink it. 5. Let him drink also JCX_0423, about half a *hexagion*, with warm wine (JCLP_099). 6. Administer a lot of dissolved unsalted lard of a goose (JCA_0543) or of a bird (JCA_0346) with a clyster without salt (JCLM_002), honey (JCA_0172) and olive oil (JCLP_053). 7. Take the juice of JCLP_045 which is called JCLP_234 which those suffering from fever drink and warm it up and add rue oil (JCLP_157) and administer with a clyster. 8. Administer a lot of table oil (JCLP_232), just warm, with a clyster without honey (JCA_0024), salt (JCLM_002) and olive oil (JCLP_053). 9. Add fenugreek (JCLP_212), either its kernels or its leaves, or as it is. Put this into a lot of table oil (JCLP_232) and let it boil and once it has warmed up, treat him with a clyster without salt (JCLM_002), honey (JCA_0024), olive oil (JCLP_053) and water (JCLM_022). 10. Add the juice of JCLP_045 and let it warm up. And then add rose oil (JCLP_181) and treat him with a clyster without salt (JCLM_002), honey (JCA_0024), olive oil (JCLP_053) and water (JCLM_022).

42.[38] For worms that is *skolekia* in the belly. Let him drink the juice of chamomiles (JCLP_227). 2. Grate the horn of a deer (JCA_0366). And let him drink these pieces with warm water (JCLM_022). 3. Let him drink rose oil (JCLP_181) with syrup (JCO_0157). 4. Boiled wormwood (JCLP_028) with warm water (JCLM_022) and let him drink. 5. Boil mint (JCLP_063) with water (JCLM_022) and let him drink it warm. 6. Crush aloe (JCLP_012) and let him drink it with warm water (JCLM_022). 7. Grind pine nuts (JCLP_097) and make them like flour and let him drink it with rose oil (JCLP_181). 8. Boil the so-called scilla (JCLP_195) with water (JCLM_022) and mix the broth with a little vinegar (JCLP_142) and let him drink. 9. Let him drink the juice of JCLP_167 that is calamint (JCLP_074). 10. Let him drink the bile of a bull (JCA_1325) with warm water (JCLM_022). 11. Let him drink ground nigella (JCLP_126) with warm water (JCLM_022). 12. Anoint the spine and the belly with the marrow of a deer (JCA_0366). 13. Boil the leaves of celery (JCLP_186) and let him drink the decoction warm.

43.[39] If the kidneys have stones. Boil JCLP_002 and maidenhair (JCLP_004) with water (JCLM_022) let him drink warm on empty stomach. 2. Boil black chickpea (JCLP_055) with water (JCLM_022) and let him drink in the morning on empty stomach. 3. Wash him frequently and when he goes into the bath let him drink the juice of JCLP_002 and maidenhair (JCLP_004) or the water (JCLM_022) of black of a chickpea (JCLP_055). 4. Grind the seed of JCLP_030 and let him drink with warm wine (JCLP_144). 5. Grind the seed of purslane (JCLP_019) that is purslane (JCLP_041) and let him drink with warm wine (JCLP_144). 6. Boil pennyroyal (JCLP_040) with wine (JCLP_144) and water (JCLM_022) and let him drink it on empty stomach. 7. Grind the seed of wild mallow (JCLP_132) and let him drink it with wine (JCLP_144). 8. Let his blood and let him be hot and he shall not drink anything cold and wind shall not hit him.

44. On dysuria. Take the so-called JCA_2703. It is a tiny very fair small bird and it walks around the twigs and the fences and the holes. It is about as small as a nut (JCLP_079). Put it into a pan. Pluck it first and disembowel it and also add crabs (JCA_0667) from the river and wine (JCLP_099) and let it boil. And let him drink warm in the morning on empty stomach. 2. Let him drink the juice of JCLP_002 and maidenhair (JCLP_004).

45.[40] For someone who urinates pus. Burn JCLP_137 and let him drink its ashes (JCX_0126) with warm syrup (JCO_0157). Boil honey (JCA_0024) and later when it is neither very dry nor very moist take normal ground linseed (JCLP_116), like flour, and crushed seed of wild cucumber (JCLP_001) and JCX_0404 and crushed pine nuts (JCLP_097), stachys (JCLP_201), celery seed (JCLP_186) like flour and put it into honey (JCA_0024). And mix these and let him eat slowly a little like the size of a nut (JCLP_079). 3. Mix JCLP_124 and the seed of liquorice (JCLP_043) with the juice of mastic (JCLP_209) and let him drink. 4. He shall eat bitter almonds (JCLP_017) at night and he shall not eat dinner. 5. Boil honey (JCA_0024) and add into it bitter almonds (JCLP_017) and ground JCX_0114 and let him eat slowly.

46.[41] If they urinate blood. Let their blood from the main vein on the elbow. 2. Let him drink the seed of a radish (JCLP_176) with vinegar (JCLP_142) and a little honey (JCA_0024). Let him drink the rennet of a hare (JCA_0453) with vinegar (JCLP_142) and a little honey (JCA_0024).

47.[42] On strangury. Strangury is if he urinates and drips a tiny bit with pain and urgency and force. Let his blood from the main vein. 2. Take the skin of a hedgehog/sea urchin (JCA_1087) that is the so-called hedgehog/porcupine (JCA_2579), where it has the skin in entirety, like big skewers. Take his skin and put it into live coals (JCLM_015) and fumigate him on the crotch, when he cannot urinate. 3. Wash him frequently. His food shall be juicy such as bullhead fish (JCA_2065), JCA_2134, JCA_2627, octopus (JCA_1939), river crabs (JCA_0667) and trout (JCA_2383). 4. Boil old summer savory (JCLP_065) with old wine (JCLP_099). Let him drink four spoon full of this. 5. Grind radishes (JCLP_176) except the leaves. Then put their juice into a pan and wine (JCLP_099) and let it boil. Then sift it vehemently and let him drink it for three days. 6. Grind dry gourd (JCLP_092) like flour the amount of one spoon. And let him drink it with cold water (JCLM_022). 7. Let rosemary (JCLP_050) boil with wine (JCLP_099) and let him drink it every day warm for ten days. 8. Drink the juice of JCLP_002 rapidly.

48. If someone urinates in the clothes at night unintentionally. Burn the throat of a crow (JCA_2386). Then crush it and make it fine like flour and let him drink in the morning on empty stomach with cold water (JCLM_036). 2. Scrape the testicles of a hare (JCA_0453) with a knife and do it very finely and let him drink it with warm wine (JCLP_144). 3. Burn the bladder of a pig (JCA_0952) that contains the urine (JCA_2332) then crush it like flour and let him drink with warm wine (JCLP_099) in the morning on empty stomach.

49.[43] For the inflammation of the liver the so-called *sykotin*. This is how you will recognise that the liver is inflamed, that there is pain on the right hand side, the pain reaches to the shoulder and the end of the flank that is the adjacent flank. There is an acute fever and he has a cough and he does not spit anything, and has thirst and anorexia and he vomits bile. Let his blood straight away from his right elbow from the main vein. Put a poultice on top of the liver. That is take barley flour (JCLP_100) and the flour of fenugreek (JCLP_212) and water (JCLM_022) and boil it and also add ground linseed (JCLP_116) like flour and ground dates (JCLP_222) and let it boil. Then spread it into a cloth (JCLP_151) and put it onto the liver. 2. Boil wormwood (JCLP_028) and roses (JCLP_181) with water (JCLM_022) and let him drink in the morning on empty stomach. 3. Boil the flower of mastic (JCLP_209) and parsley (JCLP_155) with water (JCLM_022) and let him drink it in the morning on empty stomach. 4. Grind polypodium (JCLP_165) and boil it with water (JCLM_022) and let him drink it on empty stomach. 5. Pierce him with a clyster, like warm water (JCLM_022), olive oil (JCLP_053), honey (JCA_0024) and salt (JCLM_002).

50.[44] If there is an apostem on the liver. Grind polypodium (JCLP_165) and boil it with water (JCLM_022) and let him drink it on empty stomach. 2. Take fumitory (JCLP_239) and let it boil with water (JCLM_022) and let him drink it on empty stomach.

51. [44] For the liver if it gets hard like a hard rock. Take gum ammoniacum (JCLP_015) and lard (JCA_0077) of a deer (JCA_0366) and of a bull (JCA_0466) and make a plaster and put it onto the liver. 2. Let him drink the juice of JCLP_002 and maidenhair (JCLP_004).

52. [45] On dropsy that is if the whole body swells and the belly and the feet and the testicles. For these it is appropriate that you let them drink the seed of stinging nettle (JCLP_211) ground with warm water (JCLM_022). 2. Let him drink the juice of cabbage (JCLP_098). 3. Take celery seed (JCLP_186), equisetum (JCLP_069) that is called equisetum (JCLP_163) and hazelwort (JCLP_025) and let it boil with water (JCLM_022) and let him drink every day in the morning on empty stomach just warm. 4. Let him drink the so-called JCX_0423 for two days about half a *hexagion*, just with warm wine (JCLP_099). 5. Let him also drink theriac (JCX_0301) with warm wine (JCLP_144). 6. Mix the ground dung of pigeons (JCA_2379) that has become soft like flour and fine sulphur (JCLM_033) and gum ammoniacum (JCLP_015) together and add laurel oil (JCLP_049) and mix it and apply it to the belly just warm and his spleen and the liver and the stomach from above downwards. 7. Put the plaster that is called by the doctors JCX_1903 onto the liver and another one Of Willow (JCLP_072). He shall not drink water (JCLM_022) at all nor shall he eat hearty food. He shall not have dinner and he shall not sit, but let him work as much as possible and he shall not eat in the morning but have lunch just a little bit and let him also drink wine (JCLP_099) either one or two with warm water. His food shall be like this: he shall not eat lentils (JCLP_219) nor beans (JCLP_218) nor vegetables (JCLP_112) also nor salted food, fish (JCA_0526) nor chickpeas (JCLP_055) nor beans (JCLP_220) nor fruit of the entire world nor JCX_1213 nor lard (JCA_2114) nor salted flesh (JCA_1165) nor that of a deer (JCA_1761) nor cheese (JCA_2759) neither salted nor fat, unless rock (JCLM_025) fish (JCA_1114). He should swallow [unclear word] of an egg (JCA_0105) but just the yolk (JCX_0114). Flesh (JCA_0303) of a sheep (JCA_0709) cooked meat. Everything cooked and all fresh fish (JCA_0526). If you don’t find rock (JCLM_025) fish (JCA_0526) let him eat only this. 8. Let him drink every day the juice of wormwood (JCLP_028) in the morning. Boil wormwood (JCLP_028) with water (JCLM_022) and let him drink it in the morning just warm.

53.[47] If the spleen swells. Let his blood from the left hand either on the tip of the left hand or from the little finger. 2. Boil barley flour (JCLP_100) with water (JCLM_022) and spread it out into cloth (JCLP_151) and put it onto the spleen, just add lard (JCA_0077) of a pig (JCA_0266) or of a bird (JCA_0346) and once it has boiled add lard (JCA_0077) of a goose (JCA_0543), just unsalted that does not contain any salt (JCLM_002) at all. 3. Boil the flour of fenugreek (JCLP_212), cress seed (JCLP_077) ground finely like flour and the juice of dried figs (JCLP_071) and spread it out into a cloth (JCLP_151) and put it on the spleen of the patient in the morning. 4. Make a plaster from fine linseed (JCLP_116) and dung (JCA_0254) of a goat (JCA_1403) and put it onto the spleen, instead of water (JCLM_022) add the juice of dried figs (JCLP_071).

54.[46] If the spleen gets hard. Put ground gum ammoniacum (JCLP_015) slowly into sharp vinegar (JCLP_142) and let it soak until it dissolves. Then apply to the spleen for a long time and every day in the morning just warm. 2. Grind one *hexagion* of gum ammoniacum (JCLP_015) like flour. Let him drink warm wine (JCLP_144) in the morning. 3. Grind the bark of capers (JCLP_076) and put it into vinegar (JCLP_142). And stir it and apply to the spleen in the morning, just warm. 4. Take the plant that is called centaury (JCLP_084) by the doctors, but by the locals it is called laurel (JCLP_005). Boil it with water (JCLM_022) and let him drink it in the morning on empty stomach. 5. Take a little vinegar (JCLP_142) and wine (JCLP_099) and the root of wild monk’s rhubarb (JCLP_111) and let it boil. And let him drink it in the morning on empty stomach, just warm. 6. He shall work as much as possible and he shall not eat cold things. Put the plaster called by the doctors yellow (JCO_2026) on top of the spleen and the black (JCX_2200) substance (JCX_2333).

55.[48] For icterus which the locals call *khrysiasmos*. Icterus is when the body and the eyes turn very yellow. To begin with let their blood, and let him also drink JCX_0272. 2. Take the root of monk’s rhubarb (JCLP_111) and water (JCLM_022) and let it boil and then let him drink the water (JCLM_022) water of it. 3. Boil wormwood (JCLP_028) with water (JCLM_022) and let him drink it in the morning on empty stomach. 4. Grind the seed of artiplex (JCLP_233) and mix them with water (JCLM_022) of sweet milk (JCX_0638). And let him drink it warm in the morning. 5. Let him drink JCX_0423 in the morning with wine (JCLP_099). 6. Make cinnamon (JCLP_086) like flour and let him drink it with water (JCLM_022) and a little vinegar (JCLP_142) and a little honey (JCA_0024). If the eyes are yellow, add milk (JCX_0085) of a woman (JCX_0118), like it comes out. 7. Put milk (JCX_0085) of a woman (JCX_0118) into the inside of his nose. 8. Grind nigella (JCLP_126) and mix it with vinegar (JCLP_142) and stir it. Then sift it with cloth (JCLP_151) and drip the juice into his nose. Grind cyclamen (JCLP_104) and put the juice into his nose. 9. Make barley flour (JCLP_100) and the flour of fenugreek (JCLP_212) and linseed (JCLP_116) like flour. And mix it and boil it with wine (JCLP_099) and vinegar (JCLP_142) and spread it out into a cloth (JCLP_151) and put it onto the right side that is the liver and he shall not drink wine (JCLP_099) nor shall he sit in the sun.

56.[49] If the eye of a person swells and gets big. Crush JCX_0607 of wine (JCLP_144) and roses (JCLP_181) and soft ink-gall (JCLP_085) and mix it with wine (JCLP_099) and put it on top. 2. Once you apply these, put a new sponge (JCA_0200) on top. But first macerate it with vinegar (JCLP_142) and wine (JCLP_099) and bind it strongly. Leave it bound for five days.

57.[50] For the inflammation of the testicles, that is if they are swollen. Let their blood at the bottom on the ankles of both feet. 2. Peel out the kernels of raisins (JCLP_200) and grind and also cumin (JCLP_105) and make it like flour. Then put it into the flour of beans (JCLP_218) and boil it with water (JCLM_022) and spread it out into cloth (JCLP_151) and put it on top. 3. Boil the root of asphodelus (JCLP_027) with water (JCLM_022) and then grind it well and mix it with barley flour (JCLP_100) and boil it with water (JCLM_022) and spread it out into cloth (JCLP_151) and put it on the testicles. 4. Make linseed (JCLP_116) fine like flour and boil it with syrup (JCO_0157) or the flour of fenugreek (JCLP_212) with honey (JCA_0024) and wine (JCLP_099) or the root of lily (JCLP_101) with barley flour (JCLP_100) and syrup (JCO_0157) and put it on top. 5. Mix the grated pieces of raw gourd (JCLP_092) with water (JCLM_022) and barley flour (JCLP_100) and boil it and put it on top. 6. Put the plaster that is called by the doctors JCX_1478 on top and another plaster that is called Of Humour.

58. For gonorrhoea that is if his seed flows without intercourse against his will and without coveting a woman. For these the therapy is that he shall work at all time and not have a rest and his food shall be dry that is bread (JCLP_024), water (JCLM_022), lean meat (JCA_0303) (JCA_1375). He shall not cook it vigorously and it shall not have any fat. He shall not eat anything fat nor JCX_1262 nor egg (JCA_0630) white (JCX_0101).

59.[52] If he has an erection against his will. The therapy for these is to let them drink the juice of rue (JCLP_157) and also eat it frequently. 2. Useful for these is also vomiting so that he vomits some humours before a meal and that he works hard and that he rubs his feet and hands a lot.

60.[53] For the inflammation of the small intestine. Take the juice of chicory (JCLP_068) and rose oil (JCLP_181) and lead white (JCLM_042) and crushed frankincense (JCLP_114) and make it like dough and put it into a cloth (JCLP_151). 2. Also add crumbs of bread (JCLP_024) and ground JCX_0114 and syrup (JCO_0157) and make it smooth and put it on top.

61.[54] For external haemorrhoids. If they are swollen, apply rose oil (JCLP_181) to them and once you have applied the rose oil (JCLP_181) sprinkle fine ground lead white (JCLM_042) on top and litharge (JCLM_018) or mastic (JCLP_125). 2. If they are again swollen let his blood. 3. If they are not swollen, take the leaves of cypress (JCLP_106) and burn them and make them like flour. And then wash them with warm wine (JCLP_099) then rub the ground leaves on top. 4. Burn the stones of a date (JCLP_222) and sprinkle their ashes (JCX_0126) but first wash with wine (JCLP_099). 5. Grind the shell of an egg (JCA_0105) and make it fine and plaster over on top. 6. Grind JCLP_170 finely and plaster over on top. 7. Grind nigella (JCLP_126) and make it fine like flour and first wash with wine (JCLP_099) then sprinkle the nigella (JCLP_126). 8. Burn the stones of an olive (JCLP_053) and dry gourd (JCLP_092) and rub their ashes (JCX_0126) but first wash them with warm wine (JCLP_099). 9. If there are external hemorrhoids and hang outside swollen from the blood apply the juice of cyclamen (JCLP_104). 10. If a lot blood flows from these, take the leaves of mastic (JCLP_209), the leaves of myrtle (JCLP_134), the leaves of bramble (JCLP_032), ground pomegranate (JCLP_182) that is ground peel ink-gall (JCLP_085), dry ground roses (JCLP_213) and a little vinegar (JCLP_142) and wine (JCLP_099) and let it boil. Then soak a new sponge (JCA_0200) into the juice and apply to them frequently as a warm poultice.

62.[54] For the anus if it prolapses outside. First wipe it with wine (JCLP_099) then take these finely ground things and rub them on top, that is make lead white (JCLM_042) inkgall (JCLP_085) lump of earth (JCLM_009) all fine and mix them and sprinkle them on top. Take the urine (JCX_1131) of the patient when he has a prolapsed anus hot as he urinates and wash the external hemorrhoids frequently and each day. 2. Burn the shell of of a land/sea snail (JCA_0678) and make it like flour and mix it with ground frankincense (JCLP_114) and sprinkle them on top but first wash them with warm wine (JCLP_144). Mix the flour of a land/sea snail (JCA_0678) with crushed frankincense (JCLP_114). 3. Burn the shell of sea molluscs (JCA_2326) and make it like flour and mix it with rose oil (JCLP_181) and apply to the anus. Grind ink-gall (JCLP_085) finely and the peel of pomegranate (JCLP_182) and frankincense (JCLP_114) and litharge (JCLM_018). Grind it all and make it like flour and mix it and sprinkle it on the anus. Just wash it with warm wine (JCLP_144).

63.[55] If the periods of a woman are being held which are called by the doctors months and by the women womens’ matters. What is to be done to get them moving again. Let her blood from the ankles of both feet and cup her from the parts but where you put the cup do not cut the area as many both knowledgeable people and locals do then let her drink the so-called JCX_0498. 2. Let her also drink crushed castor (JCA_0145) with warm wine (JCLP_099). 3. Boil wormwood (JCLP_028) with warm wine (JCLP_099) and let him drink. Grind rue (JCLP_157) and let her drink the juice. Let her be hot and wash her frequently just at the right time.

64.[56] On sciatica that is the so-called area of the kidneys. At the start of the disease, let his blood from his elbow from the vein that is called the main vein. 2. Grind lupine (JCLP_118) and make the flour fine and boil it with vinegar (JCLP_142). Then add also honey (JCA_0024) and spread it out into cloth (JCLP_151). And put it on top where the pain is. 3. Boil the flour of pine resin (JCLP_177) with vinegar (JCLP_142). Then add also honey (JCA_0024) and put it on top with a cloth (JCLP_151) where the pain is. 4. Grind the so-called JCLP_167 that is calamint (JCLP_074), just the leaves, and put it on top where the pain is. 5. Let him also ingest ground castor (JCA_0145) with a little vinegar (JCLP_142) and honey (JCA_0024). 6. Boil the plant that is called by the doctors centaury (JCLP_084), but by the locals it is called laurel (JCLP_005). Boil it with water (JCLM_022) and take its broth and add honey (JCA_0024) and olive oil (JCLP_053) and salt (JCLM_002) and administer an enema or the broth of lupine (JCLP_118). Once they have boiled well apply and administer. 7. Let him eat radish (JCLP_176) on empty stomach before other food and once he has eaten it after a long time let him vomit so that he expels the humours. 8. Take crushed castor (JCA_0145) and pepper (JCLP_154) and crushed euphorbia (JCLP_058) and pyrethrum (JCLP_173) and wax (JCA_0576) and all heal (JCLP_224) and rue oil (JCLP_157) and mix them and apply it warm to the area where the pain is, just every other day. 9. Spread the so-called plaster Of Laurels (JCLP_049) onto a cloth (JCLP_151) and put it on top where the pain is. 10. Make a plaster that is called JCX_1899 soft with lard (JCA_0753) of a pig (JCA_2868) and put it on top of a cloth (JCLP_151) and put it in the place where the pain is. 11. Grind mustard (JCLP_192) finely and dried figs (JCLP_071) and mix them and put it on top of the location. 12. When he comes out of the bath, apply the locations with spikenard oil (JCLP_136) or rue oil (JCLP_157) and later sprinkle it on top where the pain is. Fine and dry sulphur (JCLM_033) and crushed resin (JCLP_158) just let it be very fine like powder.

65.[57] If the fingers split. Mix liquid pitch (JCLP_244) with rose oil (JCLP_181) and apply to the split parts. 2. Boil scilla (JCLP_195) that is *scyllokormmydon* (JCLP_195) with olive oil (JCLP_053) JCO_0953 and remove the scilla (JCLP_195) and throw it away and put burnt and crushed horn (JCA_0502) of a goat (JCA_0478) into this olive oil (JCLP_053) and lard (JCA_0077) of a goose (JCA_0246) and mix it and apply to the area where they are split. 3. Mix fine lead white (JCLM_042), fine litharge (JCLM_018), fine pine resin (JCLP_093), fine frankincense (JCLP_114) and olive oil (JCLP_053) JCO_0953 and lard (JCA_0077) of a pig (JCA_0266) together and apply to the split area every day fast and slowly, just in the sun and in the house.

66.[58] For a patient, who is bed bound for a long time and his bones and his side develop wounds. Dissolve wax (JCA_0450) and add olive oil (JCLP_053) and crushed litharge (JCLM_018) and unify and apply to the area. 2. Grind all heal (JCLP_224) and make it very fine and unify it with honey (JCA_0024) and apply to the area. 3. Burn hair (JCA_0256) and sprinkle their ashes (JCX_0126) onto the lesions. 4. Boil JCX_0607 of wine (JCLP_099) with water (JCLM_022) in a pot and apply to the area. 5. Boil JCX_0607 with honey (JCA_0172) and apply to the area.

67.[59] For wounds on the feet that happen if someone wears his sandals for a month or two and does not take them off because of requirements of work and toil and the sandals rub on the feet and make wounds. Put JCA_2809 of a goat (JCA_0478) or of a male goat (JCA_1539) onto the wound. 2. Burn JCA_2387 and rub the ashes (JCX_0126) onto the wound. If the wounds swell, burn ink-gall (JCLP_085) and rub on the wound. 3. Crush the so-called acacia (JCLP_007) and mix it with vinegar (JCLP_142) and apply to the wounds.

68.[60] On callusses that appear on the hands or the feet, which the common people call *kotsia*. Grind nigella (JCLP_126) and make it fine and mix it with urine (JCX_0876) of a small child and put it on top of the calluses. 2. Mix dung beetles (JCA_1953) and crushed castor (JCA_0145) and put it on top of the calluses. 3. Dung beetles (JCA_1954) are called if there are small flies (JCA_2235) on the streets in the summer like wasps and they sit on dung and roll the dung with each other and make it round like a nut (JCLP_079) and drag this with their feet. Take such a dung beetle (JCA_1952) as it is and smash it very well with a rock. Then mix it together and put it on top on the callusses.

69.[61] For paronychitis. Paronychitis is an abscess that arises next to the root of the nail. 2. Grind ink-gall (JCLP_085) and make it like flour and mix it with honey (JCA_0024) and apply to the area. 3. Macerate fine lentils (JCLP_219) with water (JCLM_022) and put them on top. 4. Grind dates (JCLP_222) and mix them with wine (JCLP_099) and macerate and make them soft and put them on top. 5. Make green roses (JCLP_213) soft with wine (JCLP_099) and put it on top. 6. If there is blood under the nails, take the fine flour of cereal (JCLP_194) and mix it with resin (JCLP_158) and put it on top.

70.[62] For a pterygium of the nail, either of the hands or feet. Pterygium is if flesh grows on the side of the nail. Sprinkle fine yellow orpiment (JCLM_006) on top. Then bind a new sponge (JCA_0200) on top soaked with wine (JCLP_099). 2. Dry and grind the bark of pomegranate (JCLP_182) and make it like flour and mix and macerate and put it on top.

71. For the nails if they are smashed by a stone. Grind the tender leaves of myrtle (JCLP_134) and the tender leaves of pomegranate (JCLP_182) well and put them on top. 2. Take cereal (JCLP_194) flour and mix it with resin (JCLP_158) and put it on top.

72.[63] For when the nails are white as if they had psoriasis. Take oak (JCLP_052) mistletoe (JCLP_067) and put it on top. 2. Grind the flour of fenugreek (JCLP_212) and dung beetle (JCA_1951) and mix it with flour of fenugreek (JCLP_212) and a little vinegar (JCLP_142) and macerate and put it on top.

73.[63] On kelephiasis. Let their blood and mostly during spring that is in the middle of the month of March or in April or in May and let them also drink JCX_0498. When he eats radishes (JCLP_176) also ask him to vomit their entire food and he expels thick, watery and sticky humours. Let these drink vinegar (JCLP_142) mixed with cedar oil (JCLP_083). 2. Grind the leaves of cabbage (JCLP_098) and remove the juice and let him drink it in the morning and evening. 3. Burn horn (JCA_0502) of a deer (JCA_0649) and make it fine and let him drink with wine (JCLP_144). 4. File horn (JCA_0502) of an elephant (JCA_1766) and let him drink the filings with warm wine (JCLP_099). 5. Grind cumin (JCLP_105) and make it very fine and mix it with honey (JCA_0024) and a little wine (JCLP_099). And let him drink it in the morning, just warm. 6. Let him also drink theriac (JCX_0301) with wine (JCLP_099) about six kernels. 7. Grind green calamint (JCLP_074) and let him drink the juice. 8. When he has a bath and comes out, also apply either myrtle oil (JCLP_134) or vine flower (JCLP_143) or mastic (JCLP_209) olive oil (JCLP_053). Let gum ammoniacum (JCLP_015) soak with wine (JCLP_099) until it has dissolved well and apply. 9. When he has a bath, apply the entire body and the head with gum ammoniacum (JCLP_015) that has been well boiled with vinegar (JCLP_142). 10. Grind green fenugreek (JCLP_212) and apply the juice to the head and the entire body. 11. Sulphur (JCLM_033) and olive oil (JCLP_053) and salt (JCLM_002) and cumin (JCLP_105), and let it boil and apply. 12. Boil monk’s rhubarb (JCLP_111) root with vinegar (JCLP_142) and apply the broth to the head and the whole body. 13. Grind the dry leaves of a fig (JCLP_207) and make them like flour. And add fine sulphur (JCLM_033) and vinegar (JCLP_142) and stir it and apply. 14. Grind fine JCLP_240 and fine ink-gall (JCLP_085) like flour. And bone of a of cuttle fish (JCA_1305) and make it like flour and mix it and make it soft with vinegar (JCLP_142) and apply to the head and the entire body.

74.[64] For itch. For these blood letting is advised and JCX_0272 that has more black than other types. 2. Make the resin of an olive tree (JCLP_053) fine and JCLP_240 and dry the bark of the root of capers (JCLP_076) and mix it all with vinegar (JCLP_142) and apply it in the sun where the itch is. 3. Add litharge (JCLM_018) and stavesacre (JCLP_003) and sulphur (JCLM_033) and make it all fine and add olive oil (JCLP_053) and vinegar (JCLP_142) and mercury (JCLM_037) and laurel oil (JCLP_049) and mix it all and apply in the sun where the itch is. 4. Take roses (JCLP_213) and the leaves of laurel (JCLP_049) and cook. Then add wax and mix it and apply to the itching, just remove the leaves of laurel (JCLP_049) and the rose (JCLP_181). 5. Grind litharge (JCLM_018) and sumach (JCLP_242) and lead white (JCLM_042) and JCLM_031 and liquid pitch (JCLP_244) and vinegar (JCLP_142) and apply to the itch. 6. Grind the leaves of celery (JCLP_186) and put crushed sulphur (JCLM_033) into the juice and JCU_1853 and vinegar (JCLP_142) and mix it all and apply in the sun. 7. Grind the root of lily (JCLP_101) and make it fine and the seed of cress (JCLP_077) and add honey (JCA_0024) and mix and apply in the sun. 8. Make yellow orpiment (JCLM_006) fine and mix it with wine (JCLP_099) apply in the sun.

75.[64] For leprosy. Take the flour of darnel (JCLP_006) and the fine seed of cress (JCLP_077) and vinegar (JCLP_142) and apply in the sun. 2. Grind the root of asphodelus (JCLP_027) and mix the juice of the root with vinegar (JCLP_142) and apply either inside the house or in the sun. 3. Apply crushed sulphur (JCLM_033) and vinegar (JCLP_142) in the sun.

76.[65] For nerve pain. Mix lime (JCLM_007) with JCX_0607 and apply.

77.[66] For sykaminon. Grind plantain (JCLP_152) and mix it with honey (JCA_0024) and put it on top.

78.[66] For the eyes if they can’t see at night. Grind leek (JCLP_169) and mix its juice with honey (JCA_0024) and drip it into the eyes.

79. For migraine. Grind bread (JCLP_024) crumbs and mix with the bile of a cattle (JCA_1015) and vinegar (JCLP_142) and apply where it hurts.

80.[66] On lichen. Wash the bark of the root of monk’s rhubarb (JCLP_111). Then put it on top of a clay vessel, and below the clay vessel put coals (JCLM_015), so that it gets dry. Then crush it and add vinegar (JCLP_142) and apply. 2. Grind nigella (JCLP_126) and mix it with vinegar (JCLP_142) and put it on top. 3. Grind nigella (JCLP_126) and mix it with vinegar (JCLP_142) and apply. Also put it on top. 4. Crush JCU_0278 finely and mix it with liquid pitch (JCLP_244) and apply. 5. Dissolve damson tree (JCLP_047) JCLP_240 with vinegar (JCLP_142) and apply. 6. Add the leaves of capers (JCLP_076) and sulphur (JCLM_033) with crushed frankincense (JCLP_114) and add also vinegar (JCLP_142) and apply. 7. Mix sulphur (JCLM_033) with olive oil (JCLP_053) and vinegar (JCLP_142) and apply. 8. Put gum ammoniacum (JCLP_015) into vinegar (JCLP_142) and let it dissolve and then apply.

81.[67] For aporyphas. For these blood letting once or twice is possible. And at the beginning when the aporyphas are not ripe, soak a new sponge (JCA_0200) in vinegar (JCLP_142) and put it on top and bind it. Do this once or twice or thrice so that the flux is cut off. 2. Soak hair (JCA_0256) of a sheep (JCA_0708) in warm rose oil (JCLP_181) and put it on top. 3. Once you see that it is about to ripen, macerate gum ammoniacum (JCLP_015) with honey (JCA_0024) and put it on top. 4. Grind the leaves of artiplex (JCLP_233) or the seeds and mix them with honey (JCA_0024) and put it on top. 5. Grind pine resin (JCLP_177) and mix it with vinegar (JCLP_142) and macerate and put it on top. 6. Macerate propolis (JCA_0710) with vinegar (JCLP_142) and put it on top. 7. Macerate JCX_2468 with honey (JCA_0024) and apply. 8. Grind figs (JCLP_207) and macerate with honey (JCA_0024) and apply. 9. Macerate resin (JCLP_158) with honey (JCA_0024) and olive oil (JCLP_053) and apply.

82.[68] If the scar of a wound gets black. Grind litharge (JCLM_018) and white pepper (JCLP_154) and mix it with rose oil (JCLP_181) and apply to the wound. 2. Apply donkey (JCA_0619) milk (JCX_0085) onto the pain. 3. Grind the seed of rocket (JCLP_056) finely and mix it with the bile of a goat (JCA_0478) or of a cattle (JCA_1015). 4. Add also litharge (JCLM_018) and apply to the wound. Grind gum ammoniacum (JCLP_015) and make it fine and mix it with the bile of a pig (JCA_0952) and apply to the wound. 5. Dry crushed litharge (JCLM_018) and the leaves of mint (JCLP_063) and crush them and mix it with honey (JCA_0024) and apply to the area. 6. Mix crushed sulphur (JCLM_033) with white vinegar (JCLP_142) and macerate and apply it onto the wound, just apply densely.

83.[69] When the hair of the head falls out. Grind dry leaves of cabbage (JCLP_098) finely and macerate them with water (JCLM_022) and put it on the spot where the hair is falling out. 2. Grind aloe (JCLP_012) and mix it with black and austere wine (JCLP_099) and apply to the area. Grind gum ladanum (JCLP_110) and make it soft with wine (JCLP_099) and apply to the area. 3. Grind the leaves of beetroot (JCLP_189) and mix the juice with table oil (JCLP_232) and apply to the head. 4. Macerate gum ladanum (JCLP_110) with mastic (JCLP_209) olive oil (JCLP_053) and apply.

84. So that the hair of the head does never fall out, do this. Boil fern (JCLP_166) with wine (JCLP_099) and macerate gum ladanum (JCLP_110) with its juice and apply to the hair. 2. Take the plant that is called JCLP_168 and grind and mix the juice with olive oil (JCLP_053) and apply to the head.

85.[70] If you want that the hair of the head grows, do this. Dissolve gum ladanum (JCLP_110) with wine (JCLP_099) and let soak well the gum ladanum (JCLP_110) and apply to the hair. 2. Put rain (JCLM_008) water (JCLM_022) and fern (JCLP_166) and gum ladanum (JCLP_110) into a pan and gourd (JCLP_092) in dry pieces and put them into a glass (JCLM_034) vessel and let it hang in the sun for twenty days. Stir it twice a day. Stir it on every day and put your comb into the juice and comb through.

86. [71] If you want the hair to be black, do this. That is put austere wine (JCLP_099) into a pot and crushed ink-gall (JCLP_085) and JCLM_043 of iron (JCLM_027) and filings of iron (JCLM_027) and add vinegar (JCLP_142) and mix it. And put it into a glass (JCLM_034) vessel and let it hang in the sun for ten days. Then apply to the hair of the head in the morning and let it be like this again in the morning and then comb.

87. If you want to make hair blond. Put raw lupine (JCLP_118) into water (JCLM_022) and let it steep one day and one night and then apply to the hair and comb through. 2. Put white and red roses (JCLP_213) and a little crushed ink-gall (JCLP_085) and chelidonium (JCLP_231) and fern (JCLP_166) and raw lupine (JCLP_118) and lead white (JCLM_042) and water (JCLM_022) into a glass (JCLM_034) vessel and let it hang in the sun for twenty days. And stir it on the second day. If the days are over, soak a new sponge (JCA_0200) in this water (JCLM_022) and rub it onto the head.

88. [72] If there are a lot of lice on the head. Boil raw lupine (JCLP_118) with water (JCLM_022) and instead of another hot washing liquid wash him with the hot liquid of lupine (JCLP_118). 2. Grind stavesacre (JCLP_003) that is JCLP_094 finely and mix it with table oil (JCLP_232) and apply to the head. 3. Grind the leaves of ivy (JCLP_087) and remove the juice and mix it with honey (JCA_0024) and apply to the head. 4. Apply the oil (JCLP_053) of a radish (JCLP_176) to the head. 5. Grind stavesacre (JCLP_003) and make it fine and mix it with vinegar (JCLP_142) and table oil (JCLP_232) and apply to the head. 6. Macerate mercury (JCLM_037) and saliva (JCA_0904) and apply to the head. 7. Mix mercury (JCLM_037) and olive oil (JCLP_053) and vinegar (JCLP_142). 8. Mix mercury (JCLM_037) with wine (JCLP_099) and stir it and apply. 9. Mix mercury (JCLM_037) with dissolved lard (JCA_0077) of a pig (JCA_2865) and apply. 10. Grind henna (JCLP_149) and make it fine and macerate it with wine (JCLP_099) and apply. 11. Mix the bile of a sheep (JCA_0708) with wine (JCLP_099) and apply. This also helps for the eggs of lice.

89. [73] For the so-called *glykea* that appear on the head, which the women call *glykeas*. Let him drink first JCX_0272 and let his blood from the blood vessel leading to the head. 2. If they appear on the head in one location and then in another like wounds and this wound has a lot of holes, take lupine (JCLP_118) and let it boil with water (JCLM_022) and put it warm on the area where the wound is, just shave the hair first. 3. Boil the leaves of bramble (JCLP_032) with wine (JCLP_099) and lay it warm on the area. 4. Boil myrtle (JCLP_134) with wine (JCLP_099) and put it warm on the areas. 5. Boil lentils (JCLP_219) with water (JCLM_022) and a little wine (JCLP_099) and vinegar (JCLP_142) and put it warm on the area. 7. Make litharge (JCLM_018) very fine and mix it with austere wine (JCLP_099) and apply to the head, where the lesions are. But first apply a hot poultice to the area as I have told you before. 8. Burn a sheet of cotton (JCLP_029) paper (JCLP_230) and mix its ashes (JCX_0126) with austere wine (JCLP_099) and macerate it thoroughly and apply to the head. 9. Grind the leaves of rue (JCLP_157) and of myrtle (JCLP_134) and stavesacre (JCLP_003) and blue vitriol (JCLM_014) and vinegar (JCLP_142) and mix it all and apply to the lesions. 10. Make sulphur (JCLM_033) fine and mix it with table oil (JCLP_232) and vinegar (JCLP_142) and apply. Make litharge (JCLM_018) fine and also add salt (JCLM_002) and wine (JCLP_099) and olive oil (JCLP_053) and vinegar (JCLP_142) and apply to the head.

90.[74] On alopecia and ophiasis. Alopecia is called, when the hair of the head falls out. Ophiasis is called if the head becomes smooth so that it does not have any hair at all. 2. If you see that the skin of the head is almost black, let him drink JCX_0272 which has more ink than other ingredients. If you see that the skin of the head is white, let him drink JCX_0498. The same also when the skin of the head is black. If you see that the skin is neither very black nor very white, but in the middle, let him drink crushed aloe (JCLP_012) with wine (JCLP_099) and the so-called JCX_0423 about twelve kernels every day in particular with wine (JCLP_099). 3. Put pennyroyal (JCLP_040) and thyme (JCLP_066) and hyssop (JCLP_216) into a pan. And mustard (JCLP_192) and a little honey (JCA_0024) and oregano (JCLP_141) and vinegar (JCLP_142) and let the vinegar (JCLP_142) boil there, in the morning. Let him put it in his mouth in the morning and let him keep it for a long time. Do this every day. 4. Take a thick fat cloth (JCLP_151) and soak it in sharp vinegar (JCLP_142) and rub it onto the skin of his head. Rub it until the area gets red. 5. Dry the root of reed (JCLP_075) and make it fine and mix it with olive oil (JCLP_053) that remains at the bottom of the lantern, and do not put any clean one inside. 6. Dissolve the lard (JCA_0077) of a female bear (JCA_1536) and take crushed euphorbia (JCLP_058). And mix it and apply it to the head but first rub the head as I said above. 7. Make sulphur (JCLM_033) fine and mix it with liquid pitch (JCLP_244) and apply to the head. 8. Mix finely crushed seed of rocket (JCLP_056) with liquid pitch (JCLP_244) and apply to the head. 9. Make the lard (JCA_0077) of a bear (JCA_0435) and crushed pepper (JCLP_154) and nigella (JCLP_126) fine and mix and apply to the head. 10. Burn the skin of a hedgehog/porcupine (JCA_2578) and mix it with cedar oil (JCLP_083) and with lard (JCA_0077) of a bear (JCA_0435) and apply to the head. Whatever you do for alopecia and ophiasis, it is the same treatment.

91.[75] On headache. If a patient feels that the head is heavy, it is a sign that there is an increase in matter in his head. And let him drink the medication and let his blood and let him drink also JCX_0498, which the doctor called Logadios has made.

92.[75] If it appears that his head is torn apart, it is a sign that he is afflicted by pneumata, and let him drink the so-called JCX_0423. 2. Let him smell crushed pepper (JCLP_154) and cyclamen (JCLP_104).

93.[75] If it appears to him that the skin of his head is being pierced a needle and his head has a lot of heat, let him drink JCX_0272 that has more light coloured ingredients than other types of JCX_1014. Bathe him frequently with fresh water (JCLM_022). He shall not bathe in the so-called JCO_0662. For these JCO_0662 harm the head as do astringents. 2. Apply rose oil (JCLP_181) to the head. 3. Extract the olive oil (JCLP_053) of a wild olive tree (JCLP_053) and mix it with vinegar (JCLP_142) and apply to the head.

94.[76] If it appears to him that the skin of his head is cold like ice and blood coloured. Apply warm dill oil (JCLP_073) onto his head, add whatever olive oil (JCLP_053) you find from them, from crushed castor (JCA_0145) or a little euphorbia (JCLP_058).

95.[77] If he has a headache that is caused by the stomach. You will recognise that he suffers from the stomach, if the headache is sharp, such as hours and hours. 2. He is stricken by intense pain that you would say that he is suffering a heart attack/is hit in the stomach from acute pain of the skin of the head and the veins on his forehead are jumping a lot that is they pulsate, also his temples and the ears have a sound that is the so-called tinnitus. 3. Treat him with a clyster every day that is take hot honey (JCA_0024), salt (JCLM_002) and olive oil (JCLP_053). Apply mixed dill oil (JCLP_073) to the head with a little vinegar (JCLP_142). 4. Make such a patient feel comfortable first and let him vomit then let him drink the so-called JCX_0423. Put the plaster that is called by the doctors Of JCO_2615 onto his stomach. 5. Also make this heat treatment, that is put old good wine (JCLP_099) into a pan. If you do not have old one, take true astringent or white JCO_2657. Take also wormwood (JCLP_028) either its seed or the wood and crushed JCU_1344 and stachys (JCLP_201) and mastic (JCLP_125) and let it boil well. Then apply warm to his stomach as with twenty or fifteen pieces of cloth. Then let him bathe. 6. His food should be like this. Salted food on its own he shall not eat nor flesh (JCA_0303) nor fish (JCA_0526) nor pulses (JCLP_148) nor shall he have dinner. He shall only eat once per day and let him drink a little wine (JCLP_099).

96.[78,79] If he drinks a lot of wine (JCLP_099) and suffers headaches because of this. If he has still not not digested wine (JCLP_099), bring him under your control and let him vomit that is give him warm water (JCLM_022) and let him drink so that he vomits. 2. If he has a headache after he has digested, apply only rose oil (JCLP_181) or vinegar (JCLP_142) with rose oil (JCLP_181). 3. Grind the leaves of ivy (JCLP_087) and apply its juice to the head. 4. Grind the leaves of cabbage (JCLP_098) and apply the juice to the head. 5. Mix the ground leaves of cabbage (JCLP_098) with egg (JCA_0630). And put it onto his forehead. 6. Let him eat well cooked cabbage (JCLP_098) until the headache subsides. 7. Let him also eat pomegranate (JCLP_182) and apples (JCLP_131) and pears (JCLP_021). 8. If he does not want to get drunk, let him eat the stem of cabbage (JCLP_098) or bitter almonds (JCLP_017) on empty stomach.

97.[81] Anti-inflammatories that cleanse the head. Put vinegar (JCLP_142) and syrup (JCO_0157) and hyssop (JCLP_216) and pennyroyal (JCLP_040) and oregano (JCLP_141) and marjoram (JCLP_184) into a pot and let it boil and keep its juice in the mouth and he will expel a lot of fluid. 2. Grind stavesacre (JCLP_003) and make it very fine and ground pepper (JCLP_154). Take also mastic (JCLP_125) and mix it all and let him chew and he will expel a lot of fluids. 3. Mix ground ginger (JCLP_060) and stavesacre (JCLP_003) and let him chew. Ginger (JCLP_060) is called the so-called JCLP_140. 4. Take stavesacre (JCLP_003) and syrup (JCO_0157) and vinegar (JCLP_142) and pennyroyal (JCLP_040) and thyme (JCLP_066) and oregano (JCLP_141) and let it boil and let him keep it in the mouth and he will expel a lot of fluids and saliva.

98.[81] If you want to cleanse his head from liquids. Grind the leaves of beetroot (JCLP_189) and pour the juice into his nose. 2. Crush nigella (JCLP_126) finely and mix it with milk (JCX_0085) of a woman (JCX_0118) and put it into the nose. 3. Grind cyclamen (JCLP_104) and pour the juice into his nose. 4. Grind nigella (JCLP_126) and make it fine and mix it with table oil (JCLP_232) and let it rest and then rub it into his nose.

99.[82] If you want to sneeze a lot. Grind white JCU_0278 and crushed castor (JCA_0145) and fine pepper (JCLP_154) and mix it and bind it into a cloth (JCLP_151) and let him inhale. 2. Grind cyclamen (JCLP_104) and let him inhale the juice into his nose. 3. Bind crushed ink-gall (JCLP_085) into a cloth (JCLP_151) and let him inhale. 4. Grind cyclamen (JCLP_104) and let him inhale the juice into his nose.

100.[83] When you want sneezing to stop, do this. Smell at basil seed (JCLP_031) or smell at fleawort (JCLP_235) frequently.

101.[84] If his head hurts because of cold influence. Take laurel (JCLP_049) kernels and grind and make it fine and equally add seeds of rue (JCLP_157) and the root of resinous wood (JCLP_046) and vinegar (JCLP_142), and let it boil. Then add a little wax (JCA_0576) to the decoction and apply to the head. 2. Break the stone of nectarine (JCLP_179) and crush what is inside and crushed frankincense (JCLP_114) and vinegar (JCLP_142) and macerate these and put it onto the forehead with cloth (JCLP_151). 3. Crush dry cress (JCLP_077) and dry dung (JCA_0254) of a pigeon (JCA_0882) and vinegar (JCLP_142) and macerate these and put it onto the forehead with cloth (JCLP_151).

102.[84] If he has a headache because the sun has hit him a lot. Take land/sea snail (JCA_2072) and break them. And mix their flesh (JCA_0303) with crushed frankincense (JCLP_114) and vinegar (JCLP_142) and put it on the forehead with cloth (JCLP_151). 2. Pound the flesh (JCA_0303) of the land/sea snail (JCA_2073) well, then mix it. 3. Entrails (JCX_0794) of the earth (JCX_0773) that is the worms (JCA_1314) with whom they catch the fish (JCA_0526). Pound the worms (JCA_1314) and crushed frankincense (JCLP_114) and castor (JCA_0145) and vinegar (JCLP_142) and macerate and put it with a cloth (JCLP_151) onto the forehead. 4. Mix crushed euphorbia (JCLP_058) and frankincense (JCLP_114) and vinegar (JCLP_142) put it onto the forehead with a cloth (JCLP_151).

103.[85] On a head cold. If fluid runs from his palate, this is called *katarrhous*. If it is running from his nose, it is called *koryza* and if he has been struck by the sun and there is a lot of burning and it is that severe and penetrating and it appears to him that it burns him, apply to his head rose oil (JCLP_181) or olive oil (JCLP_053) from unripe olive (JCLP_053) and wash him frequently. Let him also smell at nigella (JCLP_126) and cumin (JCLP_105) both mixed and roast them a little in a new frying pan and bind them in a cloth (JCLP_151) and let him smell.

104.[85] If it happens because of cold, what is flowing, is thick and cold. Treat them like this. Let them eat a little, they should not drink anything cold. Wind shall not hit them nor smoke. Apply his head with dill oil (JCLP_073) or rue oil (JCLP_157) or spikenard oil (JCLP_136). Apply finely crushed frankincense (JCLP_114) to the inside of his nose and olive oil (JCLP_053) JCO_0953 and mix it and apply to the inside of his nose. 2. Roast nigella (JCLP_126) and cumin (JCLP_105) and bind it in a cloth (JCLP_151) and let him smell. Put the plaster that is called by the doctors JCX_2303 onto his chest or the JCX_1903 plaster, of the two what you have. 3. Apply his chest with olive oil (JCLP_053) which is called JCLP_030 or rue oil (JCLP_157) or dill oil (JCLP_073) or spikenard oil (JCLP_136) or marjoram (JCLP_184) and also add table oil (JCLP_232) and let it boil. And apply this olive oil (JCLP_053) to his chest.

105.[86] If the eye lashes fall out. Do this and they will grow. Burn the stone of a date (JCLP_222) and crush it finely and mix it with rose oil (JCLP_181) and apply. 2. Burn JCU_2793 of JCU_1945 and make it smooth and mix it with laurel oil (JCLP_049) and apply to the area. 3. Put the stones of a date (JCLP_222) into cedar oil (JCLP_083) and leave them for 15 days, the stones ground however. Then add spikenard oil (JCLP_136) and mix it with cedar oil (JCLP_083) and apply to the area.

106.[87] If you want to make the eyebrows black. Burn the horn (JCA_0502) of a male goat (JCA_2736) and make it fine and mix it with spikenard oil (JCLP_136). Apply to the area. 2. Macerate gum ladanum (JCLP_110) with wine (JCLP_099). Add also olive oil (JCLP_053) of a radish (JCLP_176) and apply. 3. Dissolve lard (JCA_0077) of a wild goat (JCA_1390) and apply. 4. There is a place called Ponticon near the so-called Morea and there grow nuts (JCLP_079). And put one nut (JCLP_079) into a new pan unbroken however. And put the pan onto live coals (JCLM_015) and let the nut (JCLP_079) boil from inside. Then remove it. And grind its slices like flour and mix them with dissolved lard (JCA_0077) of a wild goat (JCA_1389) and macerate them and mix them and apply to the area where the hair on the eyebrow is blond and you want it to be black.

107. [88] For hair if it is double on the eyebrow and you want that it is single like for all people. Tear out the double hair, then take the blood of the so-called JCA_2722 and apply. JCA_2721 is what is found on the skin of male goats (JCA_1540) in the midst of the hair like JCA_2060. 2. Slaughter a bat (JCA_2258) with gold (JCLM_041) and apply the blood to the area. 3. Apply the blood of a crow (JCA_1163) to the area. 4. Apply the blood of a frog (JCA_0764) to the area. 5. Mix crushed frankincense (JCLP_114) with the blood of a frog (JCA_0764) or of a crow (JCA_1163) and apply to the area. 6. Pound a centipede (JCA_1310) of the sea (JCX_0496) and mix it with the juice of hemlock (JCLP_109) and apply to the area. Hemlock (JCLP_109) is a plant.

108.[89] If the eye lid of a person has barley (JCLP_100) or the so-called hail stone (JCLM_038). Do this treatment, that is: Macerate finely ground sulphur (JCLM_033) with vinegar (JCLP_142) and apply. 2. Smash the head of a large of an ant (JCA_2238) on its own and make it soft with your finger and apply to the area. Crush the so-called ferula persica (JCLP_183) and make it fine and mix it with vinegar (JCLP_142) and macerate and apply to the area where the ailment is.

109. If the white of the eye gets very red and it appears that the white of the eye is bigger than the area around the pupil, this disease is called by the doctors chemosis. Treat these first with blood letting from the blood vessel leading to the head, put white (JCX_0101) of an egg (JCA_0105) or milk (JCX_0085) of a woman (JCX_0118) into the eye. 2. He shall not drink wine (JCLP_099). The sun shall not hit him, nor wind nor a sandstorm nor smoke nor shall he bathe, nor apply anything to his head.

110.[91] The emphysema is a swelling of the eye lid. This is its therapy. If the eye lid itches, warm up vinegar (JCLP_142) and wine (JCLP_099) and apply as a warm poultice with a of a cloth (JCLP_151). 2. Boil lentils (JCLP_219) with water (JCLM_022) and apply the broth to the eyes as a warm poultice. 3. If you have lard (JCA_0077) of a goose (JCA_0246) or of a bird (JCA_0346) put it slowly on the eyes. 4. Take rose (JCLP_181) and wine (JCLP_099) and let it boil and apply as a warm poultice to the eyes. 5. Take sweet wine (JCLP_099) and JCX_0114 and let it boil and fumigate the eyes. And also apply the same to the eye lid.

111.[92] On sklerophthalmia. Hardening of the eye is if he moves his eye with force and with pain and the inside of the eye is red and dry and no tears are flowing. Apply a warm poultice on them with river water (JCLM_022), warm however. Make the warm poultice with a new of a sponge (JCA_0924), afterwards put the sponge (JCA_0200) onto the eye lids. Macerate yolk (JCX_0114) of an egg (JCA_0105) with rose oil (JCLP_181) and put it onto the eyes slowly with cotton (JCLP_029). 2. If you have lard (JCA_0077) of a goose (JCA_0246) or of a bird (JCA_2320) put it slowly onto the eye. Apply table oil (JCLP_232) to the head.

112.[93] For xerophthalmia. Dry eye is when they have an itch on the eye lids and a flux from the eye. The therapy of these instances are frequent baths.

113.[94] Ectropion is if the eye lid is turned outwards. Treat it like this. Burn lead (JCLM_024) and mix it also with sulphur (JCLM_033) and apply to the eye lid.

114.[95] Aigilops is if there is an abscess near the nose in the corner of the eye. Grind the root of lily (JCLP_101) and mix it with honey (JCA_0024) and put it on top. 2. Grind lupine (JCLP_118) and sprinkle it onto the wound. 3. Grind the leaves of rue (JCLP_157) and mix them with the bile of a bull (JCA_2695) and make it like JCX_1263 and put them on top. 4. Grind the meat of a land/sea snail (JCA_0678) and mix it with crushed aloe (JCLP_012) and macerate and put it on top.

115.[96] Therapy for when the hair of the eye lids falls out. First let his blood then wash him, then let him drink JCO_1506. Then let him drink JCX_0272 that contains more of the light coloured ingredients than the other types of JCX_1014. For it happens because of an acrid humour that is because of yellow bile that the hair of the eye lids falls out. 2. Grind the stones of a date (JCLP_222) and make them very fine and crushed castor (JCA_0145) and JCX_0114 that they put into JCX_1657. And mix it with dissolved lard (JCA_0077) of a bear (JCA_0435) and apply to the area of the eye lids where the hair has fallen out. Take care however that you anoint the eye lashes conveniently. The ointment must not get into the eye and cause more damage than use. 3. He shall not eat leek (JCLP_169) nor onions (JCLP_102) nor garlic (JCLP_197) nor a lot of wine (JCLP_099) nor pepper (JCLP_154) nor pulses (JCLP_148). Let him wash without water.

116.[97] For if tears are running from the inner corner of the eye involuntarily. This disease is called by the doctors *rhoias* that is it is named after the flow. Treat these patients like this. That is take a new sponge (JCA_0200) and soak it in cedar oil (JCLP_083) and sprinkle the ashes (JCX_0126) of the sponge (JCA_0924) where the tears are flowing. 2. Again, take a new sponge (JCA_0200) and soak it in cedar oil (JCLP_083) and mix the ashes (JCX_0126) of the sponge (JCA_0924) once you have burnt it with the blood (JCX_0103) of a JCA_1717 and macerate it well and apply to the area where the tears are flowing. He shall not drink wine (JCLP_099) nor anything else that has a warm influence such as onions (JCLP_102) leek (JCLP_169) and the like, he shall not wash nor should the sun nor wind hit him.

117.[98] If there is something white on the eye. Burn the shell of cuttle fish (JCA_1305) and mix its ashes (JCX_0126) with good honey (JCA_0024) and put it into the eye. 2. Mix the bile of a cat (JCA_1407) with honey (JCA_0024) which is called JCO_0621 and macerate it well and put it into the eye. 3. His food shall be absorptive that is all fresh food. He shall not eat salted food nor pig (JCA_2867) nor fishes (JCA_0594) nor pulses (JCLP_148) nor vegetables (JCLP_112) nor the so-called JCX_1213 nor white (JCX_0101) of an egg (JCA_0105) except yolk (JCX_0114) on its own.

118.[99] For the pterygion of the eye. Pterygion is if a small piece of flesh grows from the inner corner of the eye and it constricts and it spreads towards the pupil. This is called *pterygion*. Do this treatment. That is take the so-called magnet (JCLM_020) and crush it and make it very fine and crushed JCX_0114 and good honey (JCA_0024) and unify and macerate it well and apply to the area where the flesh is. 2. Grind JCLP_204 the so-called JCLP_038 and mix it with the flour of lupine (JCLP_118) and with good unsmoked honey (JCA_0024) and mix it and macerate them well and apply to the flesh just this one and alone.

119.[100] For blindness and trouble seeing. Blindness is called if the patient doesn’t see at all without a disease. Treat them like this. First blood letting and baths. Let him also drink JCX_0272 and wind shall not hit him, nor sun nor a dust storm nor smoke nor shall he eat pulses (JCLP_148) nor salted food nor vegetables, unless it is meat based fresh food and cooked food and yolk (JCX_0114) on its own of an egg (JCA_0105) and a little hot wine (JCLP_099).

120.[100] Amblyopia is if he sometimes sees a little sometimes very very little and sometimes not at all. Let his blood from the tip of the eye near the nose, which the doctors call corner. Put leeches onto the temples if both eyes are affected by the disease. If one side is diseased, do it on one. 2. Also treat him with a clyster on each day. 3. Apply old olive oil (JCLP_053) and good honey (JCA_0024) on the outside of the eyes. 4. First wipe the eyes with water (JCLM_022) and salt (JCLM_002), just hot with a sponge (JCA_0923). Then grind the leaves of fennel (JCLP_123). And put ground JCX_0114 into their juice and the bile of a female pig (JCA_2600) and honey (JCA_0024) and crushed pepper (JCLP_154) and crushed gum ammoniacum (JCLP_015) and mix it all and apply to the eyes on the outside. 5. Grind the leaves of rue (JCLP_157) and fennel (JCLP_123) and mix their juice with unsmoked honey (JCA_0024) and make it smooth and apply to the eyes. 6. Let him also eat the stem of cabbage (JCLP_098) raw. Harmful to them are dill (JCLP_073), lentils (JCLP_219), black olives (JCLP_053), lettuce (JCLP_119), leek (JCLP_169), basil (JCLP_031), vegetables (JCLP_112), celery (JCLP_186). 7. Grind nigella (JCLP_126) and mix it with water (JCLM_022) and make it good and smooth and apply to the eyes.

121. When there is something white on the eye. Let his blood from the blood vessel leading to the head. Let him drink also JCX_0272. Cup him on the head. Let him drink three times mixed wine (JCLP_099). 2. Crush stavesacre (JCLP_003) and pepper (JCLP_154) and mastic (JCLP_125) and wax (JCA_0450) and mix it all well and let him chew. 3. Put vinegar (JCLP_142) into a vessel and thyme (JCLP_066) and oregano (JCLP_141) and pennyroyal (JCLP_040) and hyssop (JCLP_216) and let it boil well. And in the morning let him take from it into his mouth and let him keep it for several hours and then let him spit it out. And he shall expel a lot of saliva and spit that is dribble. He shall not sit in the sun nor shall wind hit him nor smoke. 4. Let them drink the so-called JCX_0423 with warm wine (JCLP_099). 5. Grind fennel (JCLP_123) and mix the juice with a little table oil (JCLP_232) and honey (JCA_0024) and apply to the eyes externally. 6. Crush white JCU_0278 and make it very fine. And add honey (JCA_0024) and mix it and apply to the eyes externally. 7. Grind the plant called tordylium (JCLP_081) and mix the juice with honey (JCA_0024). Apply to the eyes externally.

122.[101] If the area under the lower eye lid gets black. This disease happens mostly in women and in children. This disease is called by the doctors *hypopion*. Do this treatment. That is grind radish (JCLP_176) and put it on top. If the radish (JCLP_176) eats away the area of the wound, cup it. 2. Grind fat cheese (JCA_2761) and put it on top of the wound. 3. Grind green wormwood (JCLP_028) and remove its juice and add wax (JCA_2021) and dissolve it. Then add chamomile oil (JCLP_227) and rose oil (JCLP_181) and lard (JCA_0077) of a goose (JCA_0246) and of a bird (JCA_0346), then add the juice of wormwood (JCLP_028) and stir it until it gets cold. Then put it onto the wound. 4. Grind the green leaves of wormwood (JCLP_028) and put them on top. 5. Crush litharge (JCLM_018) and mix it with honey (JCA_0024) and put it onto the wound. 6. Dissolve wax (JCA_0450) and add chamomile oil (JCLP_227) and crushed mustard (JCLP_192) and mix it and put it onto the wound. 7. Bind crushed cumin (JCLP_105) into a cloth (JCLP_151) and soak it in hot vinegar (JCLP_142) and fumigate the area. 8. Dissolve wax (JCA_0450) and add rose oil (JCLP_181) and crushed wormwood (JCLP_028) and frankincense (JCLP_114) and mint (JCLP_063) and litharge (JCLM_018) and mix it all and apply to the wound.

123.[101] If the lower eye lid gets red like blood or the colour of the area is like yellow. Grind the leaves of hyssop (JCLP_216) and raisins (JCLP_200) from their kernels and put on the wound. 2. Grind wormwood (JCLP_028) and put it on top. Grind oregano (JCLP_141) and mix it with honey (JCA_0172) and put it on top. 3. Grind JCLP_036 and mix it with honey (JCA_0024) and put it on top. 4. Grind cyclamen (JCLP_104) and put it onto the wound, where the red or yellow area is. He shall not drink wine (JCLP_099). Neither the sun nor the wind shall hit him. Let his blood from the blood vessel leading to the head.

124.[102] When there is pain from cold. Crush castor (JCA_0145) very finely and mix it with rue oil (JCLP_157), drip it into the ear. 2. Mix very fine castor (JCA_0145) with spikenard oil (JCLP_136) and drip it into the ear. 3. Mix fine castor (JCA_0145) with laurel oil (JCLP_049) and drip it into the ear. 4. Mix fine castor (JCA_0145) with marjoram (JCLP_014) olive oil (JCLP_053) and drip it into the ear. 5. Mix very fine pepper (JCLP_154) with spikenard oil (JCLP_136) and drip it into the ear. 6. Mix fine castor (JCA_0145) with olive oil (JCLP_053) that is called JCO_2825 and drip it into the ear. 7. Mix table oil (JCLP_232) with crushed euphorbia (JCLP_058) and drip it into the ear. 8. JCLP_080. Mix what I said above with olive oil (JCLP_053) and drip it into the ear. 9. Grind crushed garlic (JCLP_197) well with table oil (JCLP_232). Then let it get warm and drip into the ear. 10. Grind onion (JCLP_102) well and boil it in the shell of a JCA_2293 or mussel (JCA_1245) with table oil (JCLP_232) and drip it into the ear. 11. The wind shall not hit him, nor shall he drink cold things nor shall he eat pulses (JCLP_148) nor salted food.

125.[103] If there is pain in the ear because of a hot imbalance, like from the sun and heat and a lot of exhausting activity. Mix white (JCX_0101) of an egg (JCA_0105) with rose oil (JCLP_181) [two partly legible words] and drip it into the ear. 2. Mix white (JCX_0101) of an egg (JCA_0105) with rose oil (JCLP_181) and a little vinegar (JCLP_142) and warm it up and drip it into the ear.

126.[103] If there is pain inside the ear through hot imbalance. Mix white (JCX_0101) of an egg (JCA_0105) with the juice of JCLP_204, which is called by the locals JCLP_038, and warm up this juice with an egg (JCA_0105) and drip it into the ear. 2. Pound the root of asphodelus (JCLP_027) and mix the juice with a little rose oil (JCLP_181) and drip it warm into the ear. 3. Drip almond oil (JCLP_017) into the ear. 4. Mix milk (JCX_0085) of a woman (JCX_0118) with rose oil (JCLP_181) and a little vinegar (JCLP_142) and drip it warm into the ear. 5. Mix syrup (JCO_0157) with rose oil (JCLP_181) and drip it warm into the ear. 6. Warm up syrup (JCO_0157) and a little vinegar (JCLP_142) and warm it up and drip it into the ear. 7. Mix milk (JCX_0085) of a woman (JCX_0118) with the juice of JCLP_038 and drip it warm into the ear. 8. Mix the juice of asphodelus (JCLP_027) with rose oil (JCLP_181) and milk (JCX_0085) of a woman (JCX_0118) drip it into the ear. He shall not sit in the sun and wind shall not hit him.

127.[104] If there is pain in the ear through thick humours. You will recognise that the pain comes from thick humours from there being heaviness in the head and the ears having *akhos*. If the head is not heavy and it appears as if the skin of the head is stretched and this is the only sign, this is a disease of the ear and not of the head. Do this treatment. That is take vinegar (JCLP_142) and honey (JCA_0024) and warm it up and drip it into the ear. 2. Mix the bile of a sheep (JCA_0708) with almond oil (JCLP_017) and warm it up and drip it into the ear. 3. Pound leek (JCLP_169) and mix the juice with good honey (JCA_0024) and warm it up and drip it into the ear. 4. Grind onion (JCLP_102) and mix the juice with honey (JCA_0024) and drip it into the ear also warmed up. 5. Mix the juice of the plant JCLP_051 with honey (JCA_0024) and drip it into the ear.

128.[105] If the ear is inflamed. Do this treatment. That is let his blood and apply a warm poultice with table oil (JCLP_232). And warm up syrup (JCO_0157) and soak hair (JCA_0256) of a sheep (JCA_1282) from the area of the testicles, so that they are unwashed. And apply as a warm poultice to the area. 2. Drip warm dissolved lard (JCA_0077) of a goose (JCA_0246) into the inside of the ear. 3. Drip warm unwashed lard (JCA_0077) of a fox (JCA_1448) into the ear. 4. Mix very crushed castor (JCA_0145) with milk (JCX_0085) of a woman (JCX_0118) and warm it up and drip it into the ear. 5. Warm up syrup (JCO_0157) and drip it into the ear. 6. He shall not drink a lot of wine (JCLP_099), this is almost to say none at all. Let him be hot. The sun shall not hit him nor wind nor smoke.

129.[106] If you want that the teeth of a child grow. Do this treatment. That is anoint the gums of the teeth with a female dog’s (JCA_1315) milk (JCX_0085) and the teeth will come out. 2. Anoint the marrow of a hare (JCA_0453) from his head onto the gums and the teeth will come out. 3. Anoint the urine (JCX_1131) of a small child to the gums just mixed with milk (JCX_0085) of a female dog (JCA_1315).

130.[131] If there is a tumour under the tongue. This tumour happens more often on the blood vessels of the tongue. Do this treatment. That is mix finely crushed inkgall (JCLP_085) with syrup (JCO_0157) and apply it to the swelling. 2. Mix very fine and sieved vetch (JCLP_178) with honey (JCA_0024) and apply it to the swelling where it is on the veins of the tongue. 3. Grind the seed of roses (JCLP_213) and make it fine and mix it with honey (JCA_0024) and apply to the swelling. 4. Pound the leaves of an olive tree (JCLP_053) and apply the juice to the tongue where the disease is. 5. Keep the juice of the leaves of an olive tree (JCLP_053) on the tongue for a long time.

131.[132] Aphtha is if the lips of children when they are breastfeeding are eroded or because the milk (JCX_0085) of a woman (JCX_0362) is acrid and they are eroded because of that. This also happens to adults. Do this treatment. Take lentils (JCLP_219) and grind and mix it with bread (JCLP_024) crumbs and macerate with wine (JCLP_099) and apply to the lips. 2. Apply marrow (JCA_0307) of a deer (JCA_0366) to the lips. 3. Apply marrow (JCA_0307) of a male calf (JCA_0515) to the lips. 4. Apply the juice of pears (JCLP_021) to the lips. 5. Pound chicory (JCLP_068) and apply the juice to the lips. 6. Burn the horn of a deer (JCA_0366), pound it and apply to the lips. 7. Burn the root of fennel (JCLP_123) and sprinkle the ashes (JCX_2632) onto the lips. 8. He shall not drink much wine (JCLP_099). Let their blood if it is big.

132.[133] If the so-called jaws are inflamed. Do this treatment. That is let his blood from the blood vessel leading to the head and once ten days have passed, let his blood from the middle vein, some call this basilik vein the main vein because this vein exceeds the other veins and leads the other veins. These patients shall not eat much. They shall not drink much. 2. Boil lentils (JCLP_219) and roses (JCLP_181) and dates (JCLP_222) with wine (JCLP_099) and once it has boiled well keep the wine (JCLP_099) in the mouth for a long time. 3. When you let his blood, after one day, take liquorice (JCLP_043) and syrup (JCO_0157) and figs (JCLP_207) and let it boil and later add rose water (JCLP_181) and keep it in the mouth. 4. Take hyssop (JCLP_216) and the juice of pomegranate (JCLP_182) and mix it and let it boil a little. Then add JCLM_010 and keep it in the mouth. 5. Keep sweet milk (JCX_0085) of a sheep (JCA_0709) in the mouth. 6. Grind water (JCLM_022) rain water (JCLM_008) and soak a new sponge (JCA_0200) in this water (JCLM_022) and apply as a warm poultice to the area for a long time. 7. Let rain (JCLM_008) water (JCLM_022) boil and soak raw JCU_2097 and apply as a warm poultice to the area. 8. Boil barley flour (JCLP_100) and fine sifted linseed (JCLP_116) with water (JCLM_022) and a little wine (JCLP_099). Put it with cloth (JCLP_151) on top of the area. Do this three days in the morning and evening. 9. Pound dates (JCLP_222) well. Mix it with the flour of fenugreek (JCLP_212) and add water (JCLM_022) and a little wine (JCLP_099) and boil it and put it on the location. 10. Boil liquorice (JCLP_043) and JCX_0114 and wine (JCLP_099) and let it boil and keep it in the mouth.

133.[134] For cough. Let him drink in the evening the socalled storax (JCLP_206) in the equal amount of a chickpea (JCLP_055) and he shall not have a meal. The same so-called storax (JCLP_206) also helps for a runny nose. 2. In the morning before he eats let him take dry fat figs (JCLP_071) and let him chop them up into small pieces and let him put them into unmixed good wine (JCLP_099) and let him eat. Once he has completed this, let him drink on top a little sweet wine (JCLP_099). 3. Five figs (JCLP_207) in the morning in sweet wine (JCLP_099) and let him add also a little water (JCLM_022) and let him drink. 4. Let them also make the so-called JCΧ_1669, that is let him put hyssop (JCLP_216), pennyroyal (JCLP_040), calamint (JCLP_074) and oregano (JCLP_141) into this millet (JCLP_082) that boils and let it boil well and remove it and also take good honey (JCA_0024) and stir it and let him eat. Let him throw away the plants. Let him drink it until the cough resolves, and do not let him drink anything cold nor shall wind nor sun hit him. He shall be hot as much as possible and let him drink warm wine (JCLP_099) and shall not have a meal and when he wants to go to sleep, let him eat five bitter almonds (JCLP_017) in the evening. If he doesn’t have bitter ones, let him eat ten sweet ones and let him drink warm wine (JCLP_099) in addition. If you also do not have sweet ones let him eat three dates (JCLP_222). 5. Boil good honey (JCA_0024) and boil it down and candy it so that it is neither very moist nor very dry but in the middle. And then add 100 ground sweet almonds (JCLP_017) and 20 ground JCLP_108, ground JCX_0404 and twenty ground bitter almonds (JCLP_017). And if he does not have fever take also ground pepper (JCLP_154) just coarse. And very finely ground liquorice (JCLP_043) and half a spoon of fine linseed (JCLP_116) and ground fat dates (JCLP_222) ground dry leaves hyssop (JCLP_216) and mastic (JCLP_125) and ginger (JCLP_060), the so-called JCLP_140, and this very fine. And very fine stachys (JCLP_201) and fine JCU_1344 which is called cinnamon (JCLP_086) and a little fine cumin (JCLP_105) and white pepper (JCLP_154) that is eaten and the so-called long pepper (JCLP_121) just very fine one. And ground seed of stinging nettle (JCLP_211) and make it fine and unify it all and add it all once you take out the JCU_2719 where there is honey (JCA_0024) and once you add the articles do not boil. In the evening, let him eat from it the equal amount of a nut (JCLP_079), and then let him drink very good wine (JCLP_099). 6. Apply a little butter (JCA_0330) of a sheep (JCA_0708) to his chest and do not rub it at all as you will cause more damage than good. 7. Apply lard (JCA_0077) of a goose (JCA_0246) or of a bird (JCA_0346) to his chest. 8. Grind all heal (JCLP_224) and make it very fine and very fine castor (JCA_0145) and mix it and mix the three and also add a little wine (JCLP_099) as much as it takes to make a hard dough. And then make small kernels like chickpeas (JCLP_055) and smaller and let him swallow in the evening when he is about to go to sleep. And he shall not have dinner. On top of this let him drink hot good wine (JCLP_099). Let him drink in the same way during the night. Put a plaster Of Butter (JCX_0554) onto his chest and another plaster which is called JCLP_073. And put the one of the two plasters that you can find on his chest with cloth (JCLP_151) or with JCU_2179.

134.[111] For those which a very hoarse voice. Do this treatment. That is wash him frequently and his diet shall not be acrid like leek (JCLP_169) and the like, neither salted flesh (JCA_0303) or fish (JCA_0526) unless all that is fresh. 2. Let him also eat JCX_0404 and eggs (JCA_0761) drunk raw, and sweet warm milk (JCX_0085) of a sheep (JCA_0709).

135.[112] For those who happen to have difficulty breathing and cannot take a breath and are mostly doing poorly when it is cold. Do this treatment. That is scilla (JCLP_195) that is called by the doctors *scylla* (JCLP_195) and by the Lombards *scal* (JCLP_195), boil this with vinegar (JCLP_142). Let him also add water (JCLM_022) and let him drink it instead of wine (JCLP_099). 2. Boil scilla (JCLP_195) in coals (JCLM_015) and take its peels one by one. And dip them into honey (JCA_0024) and a little vinegar (JCLP_142) and let him eat them. 3. Let him drink it and the so-called JCX_0498. 4. Let him eat radishes (JCLP_176) and then let him vomit, so that he expels the humours. 5. Grind the seed of JCLP_167 and make it fine let him drink in the morning with warm wine (JCLP_144). 6. If it has been a long time that his blood has not been let, let his blood then. 7. Put the flour of darnel (JCLP_006) onto his chest and water (JCLM_022) and macerate and also add pounded figs (JCLP_207) and macerate them and put it onto his chest with cloth (JCLP_151). 8. Make the fruit of hyssop (JCLP_216) fine and let him drink it with warm wine (JCLP_144), and apply sometimes dill oil (JCLP_073) sometimes rue oil (JCLP_157) to his chest. 9. Boil wormwood (JCLP_028) with wine (JCLP_099) and mix it with ground castor (JCA_0145) and make about five pills. And let him ingest these in the morning. 10. Crush gum ammoniacum (JCLP_015) and let him drink with hot wine (JCLP_144). 11. Let him be hot as much as possible and he shall not drink anything cold nor shall the wind hit him.

136.[123] If someone spits a lot of blood and in one go, this is a sign that he has burst a blood vessel for instance if someone falls from a height and vomits or spits blood. Let him drink lump of earth (JCLM_009) with water (JCLM_022). If he is similar to bedridden and he spits blood let his blood. 2. If he spits blood it is a sign that the blood comes from the airways. And let his blood. Also soak a new sponge (JCA_0200) in vinegar (JCLP_142) and apply as a warm poultice to the chest. 3. Soak hair (JCA_0256) of a sheep (JCA_1282) from the testicles so that they are unwashed in warm rose oil (JCLP_181) and apply as a warm poultice to the area. 4. Boil the leaves of myrtle (JCLP_134) with vinegar (JCLP_142) and apply as a warm poultice to the chest. 5. Soak a sponge (JCA_0200) in austere warm wine (JCLP_144) and apply as a warm poultice. 6. Put the plaster that is called by the doctors Of Willow (JCLP_072) onto the chest. Let him drink crushed lump of earth (JCLM_009) with warm water (JCLM_022). 6. Grind Lemnian sealed medication (JCLM_032) and let him drink it with wine (JCLP_099) on empty stomach. 8. Let the patient drink the juice of pomegranate (JCLP_182). 9.Boil dates (JCLP_222) with water (JCLM_022) and the juice should have grated onion (JCLP_102) and let him drink.

137.[112] On pleuritis. If there is pain and it reaches towards the shoulder, let his blood. If the pain is in the belly or the intestines let him drink JCX_0272. 2. Boil the socalled centaury (JCLP_084), but by the locals it is called laurel (JCLP_005). Boil it also with bran (JCLP_160) and administer their juice also as a clyster with honey (JCA_0172) and olive oil (JCLP_053) and salt (JCLM_002). 3. Roast millet (JCLP_082) in a pan and apply as a warm poultice to the area. 4. Boil unwashed hair (JCA_0256) in warm wine (JCLP_099) and table oil (JCLP_232) and apply as a warm poultice to the area. 4. Add the so-called chamomile (JCLP_227) and wormwood (JCLP_028) and iris (JCLP_070) and water (JCLM_022) and let it boil. And add water (JCLM_022) into barley flour (JCLP_100) and into flour of linseed (JCLP_116) and boil it and wash it into a cloth (JCLP_151) and put it where the pain is. Once the seventh day has passed, make an electuary with sweet of almonds (JCLP_017) and roast the crushed seed of stinging nettle (JCLP_211) barley (JCLP_100) and apply as a warm poultice to the area. 5. Add the plaster called marjoram (JCLP_184) onto the area where the pain in the side is. 6. Put the plaster that is called by the doctors Of Rue (JCLP_157) also on the area where the pain is. 6. If you do all this and there is again pain in this area where it was first cup him on top of the location where he is in pain just as necessary. 7. Dissolve lard (JCA_0077) of a pig (JCA_0266) and dissolved wax (JCA_0450) and add sulphur (JCLM_033) and henna oil (JCLP_107). This is also called butter (JCA_0330). And crushed chickpea (JCLP_055) and mix it up and macerate it and make it like a wax plaster and put it on the place where the pain of the flanks is. 8. Put rue (JCLP_157) and hyssop (JCLP_216) and calamint (JCLP_074) and the so-called iris (JCLP_070) into JCX_1205. And the so-called lavender (JCLP_202) and the so-called ground pine (JCLP_228) and a little crushed sumach (JCLP_175). However, first let the herbs boil, then add the juice, sumach (JCLP_175) about six kernels and let him drink every evening a little sumach (JCLP_175) [two partly legible words] its juice.

138.[113] On inflammation of the area of the lungs. Pneumonia is a disease where he coughs and spits thick yellow pus. It smells sometimes a little, sometimes a lot and sometimes a little bit. Let his blood. And make sure he does not pass out. Let him drink the juice of JCLP_045. Let him also eat wheat flour (JCLP_187). Let them drink almond extract (JCLP_245) with sweet almonds (JCLP_017) and JCLM_010 and the so-called JCX_0985. 2. Boil hyssop (JCLP_216) with dried figs (JCLP_071) and reed (JCLP_075) ...... and water (JCLM_022). And let it boil and let him drink it. 3. Put onto the chest of the patient that is onto the thorax a wax based plaster called JCX_2394 that is take wax (JCA_0576) and let it dissolve. Then take lard (JCA_0077) of a goose (JCA_0246) or of a bird (JCA_0346) or deer (JCA_0793) marrow (JCA_0307). Instead of table oil (JCLP_232) add rue oil (JCLP_157) and macerate and stir thoroughly until it has all dissolved. If you don’t have rue oil (JCLP_157), add the so-called henna oil (JCLP_107) or table oil (JCLP_232).

139.[114,115] Empyema is called if pleuritis has a major appearance and he coughs and spits quickly something like pus and cough and the saliva (JCA_0904) smells bad. Take hyssop (JCLP_216) and thyme (JCLP_066) and water (JCLM_022) and let the patient drink. 2. Take honey (JCA_0024) and let it boil and add crushed liquorice (JCLP_043) and crushed sesame (JCLP_190) into honey (JCA_0024) inside and stir it and let him eat in the evening little by little and on top let him drink boiled wine (JCLP_099). 3. Give him also the antidote Of JCLP_241 and the antidote that is called Of Vetch (JCLP_178). Take honey (JCA_0024), let it boil and later add sweet almonds (JCLP_017) and JCLP_097 into honey (JCA_0024) and let him eat it in the evening little by little and on top let him drink warm wine (JCLP_099) but he shall not have a meal at all.

140.[115] When the disease called *empyema* leads to the very disease that the locals call *phthisis* which the doctors call consumptive disease. Do this treatment. That is the so-called antidote Of Esdra. Put a wax plaster on top of his chest that is called Of butter (JCA_0554) and Of Henna (JCLP_107) and the plaster that is called JCX_0854. Give this patient the antidote that is called JCX_1862 to drink and theriac (JCX_0301).

141.[115] Where it comes and someone has a headache and fever. Make the same juice with JCLP_045. And also add raisins (JCLP_200) without the kernels and Syrian jujube (JCLP_061) and damson (JCLP_047) and two or three dates (JCLP_222). After it has boiled, add mastic (JCLP_125) and sugar (JCLP_185). If you don’t have sugar (JCLP_185), add a little honey (JCA_0024).

142.[115] Phthisis is called, if the lung of a patient makes ulcerated matter and he spits ulcerated matter and it stinks and withers away and the body ....... and he spits ulcerated matter with blood, like foam. If he spits something like this, it is a sign that the lung is rotting. Do this treatment. That is let him drink milk (JCX_0085) of a donkey (JCA_0619) and of a woman (JCX_0118) as Galen says and writes. Put JCX_1140 Of Butter (JCA_0554) on top of the chest. And JCX_1141, if possible take laurel oil (JCLP_049) instead of table oil (JCLP_232) and make it and add wax (JCA_0450) and let it dissolve. And then add instead of table oil (JCLP_232) laurel oil (JCLP_049). 2. Apply rue oil (JCLP_157), dill oil (JCLP_073) to his chest. Then put also the plaster that is called by the doctors JCX_0854. 3. Let them drink the so-called JCX_1205 with JCX_0404. Or let liquorice (JCLP_043) boil with water (JCLM_022) and let him drink. 4. Give him also milk (JCX_0085) of a cow (JCA_0769) or of a goat (JCA_0478). But leave out milk (JCX_0085) of a donkey (JCA_0619) and of women (JCX_0491). 5. Take honey (JCA_0024) and let it boil and sweet crushed almonds (JCLP_017) and JCLP_108 and pistachios (JCLP_159) and crushed JCX_0114 and bitter almonds (JCLP_017) and sesame (JCLP_190) and the pounded flesh of a date (JCLP_222). 6. Let these patients drink the antidote that is called JCX_0964 and the so-called JCX_1911 and the so-called antidote Of Esdra and theriac (JCX_0301) about three or four kernels with a little wine (JCLP_099) in the morning.

143.[116] If someone first gets enraged by fever and thus the person becomes feverish. From the first day onwards wash him and apply table oil (JCLP_232) to the whole body and after you wash him, feed him and on the next day let him carry on with whatever work he may have. Do the same therapy if someone faints and has fever of mental origin or from exhaustion or from heat stroke. Do the same therapy which you have done if someone is enraged.

144.[117] For tertian fever. For tertian fever if he has a remission on one day and a fever on the other. And for those people it is necessary to do this therapy. That is lettuce (JCLP_119), chicory (JCLP_068) with vinegar (JCLP_142) and water (JCLM_036) and yoghurt (JCA_0870). 2. Also treat him with a clyster. That is boil olive oil (JCLP_053), honey (JCA_0024), salt (JCLM_002), mallow (JCLP_132) and boiled bran (JCLP_160) and add their juice instead of hot water (JCLM_036). 3. He shall not drink wine (JCLP_099) nor shall he eat salted food nor flesh (JCA_0303) nor fish (JCA_0526) nor anything else that is salted. 4. Let them drink the root of celery (JCLP_186) and the root of dill (JCLP_073) and the root of JCLP_002, washed, and chicory (JCLP_089) and agrimony (JCLP_057) and equisetum (JCLP_069) the so-called equisetum (JCLP_163) and rosemary (JCLP_050) and let it boil and let him drink it instead of water (JCLM_022). After it has boiled add mastic (JCLP_125) and instead of honey (JCA_0172) sugar (JCLP_185). Let these drink boiled wormwood (JCLP_028) with water (JCLM_022) in the morning. 5. When it is time for a bath, bathe him in hot fresh water not the one they call JCO_0662. By Galen they are called JCLM_003. 6. If there happens to be such a patient who likes to bathe you will not cause him any harm if you bathe him every other day or day after day. 7. Let these also drink JCX_2771 and the so-called *zoulapin* (JCX_1849) with cold water (JCLM_036).

145.[118] For tertian fever. Tertian fever is if he has a fever for two days and a remission for one day. It comes from black bile, which is dry and cold. This humour is outside the blood vessels. Do this therapy. That is to begin, do not let his blood and do not purge. If it has been several days that his blood had not been let, let a small amount of blood evacuate, and if you see that the blood is black, let it run, but make sure that he does not pass out. If you see that it is light coloured, stop it immediately and give him useful food such as fish (JCA_0526), flesh (JCA_0303) just fresh and fresh things to follow. 2. Boil pennyroyal (JCLP_040) and the so-called dodder (JCLP_229) that is what is on top of a thyme (JCLP_066). Boil with water (JCLM_022) and take their juice and honey (JCA_0024) and olive oil (JCLP_053) and salt (JCLM_002) and pierce him with a clyster. And once you make this clyster, and three days have passed, let him drink the so-called by the doctors Of the Three Peppers (JCLP_154) or the so-called JCX_0783. 3. Let the patient drink crushed pepper (JCLP_154) with warm water (JCLM_022). Let the patient drink the following, that is take hazelwort (JCLP_025) and maidenhair (JCLP_004) and celery seed (JCLP_186) and equisetum (JCLP_069) that is called equisetum (JCLP_163) and chicory (JCLP_089) and rosemary (JCLP_050) and agrimony (JCLP_057) and water (JCLM_022) and let it boil. And once they have boiled, later add mastic (JCLP_125) and instead of honey (JCA_0172) sugar (JCLP_185) and let him drink. 5. Onto the intestines that is the spleen put the plaster called by the doctors JCX_2058 or the so-called JCX_2877 and the so-called JCX_2438. 6. Also give the antidote that is called by the doctors Of JCX_1872. And the other antidote that is called JCX_2236. 7. First evacuate the black bile through the stomach, then let him drink the antidote called theriac (JCX_0301) and the medication of JCX_0078 which was made by Kyrenaikos. 8. Let him also eat salted food such as flesh (JCA_1165) of a pig (JCA_2866) and fishes (JCA_0594) and mustard (JCLP_192) and other warming things like pepper (JCLP_154) garlic (JCLP_197) and other similar things. This disease holds on for a long time in the body of a person and still does not cause death.

146.[119] For daily fever. In daily fever bring him at the start, and let him drink, the so-called JCX_1239 and diuretic medication such as take the root of celery (JCLP_186) and agrimony (JCLP_057) and fumitory (JCLP_239) and water (JCLM_022) and the roots of chicory (JCLP_089), and once they have boiled, later add instead of honey (JCA_0172) sugar (JCLP_185) and let him drink. 2. First let him drink the so-called JCX_1239, then let him eat radishes (JCLP_176) and let him vomit his food and radishes (JCLP_176) so that he expels some sticky and thick humours which cause the daily fever. Let him also eat cumin (JCLP_105) and pepper (JCLP_154) and other warming substances. Do not let him drink anything cold nor let him eat anything cold and let everything be hot.

147.[120] For the disease that is called fever disease if there is a pestilential disease that the locals call fever disease. Do this treatment. First of all treat him with a clyster. And if there are several days that he did not have blood letting let a little blood out. Add celery (JCLP_186) and equisetum (JCLP_069) that is called equisetum (JCLP_163) and chicory (JCLP_089) and let them boil with water (JCLM_022) and let him drink. 2. Let him also drink the juice of chamomiles (JCLP_227) with water (JCLM_022) once it has boiled. 3. If he burns inside his cavities a great degree, let him drink cold water (JCLM_022) as much as he likes so that it extinguishes the flame that he has. 4. Boil also water (JCLM_022) and once it has boiled let it get very cold and let him drink it. 5. Let him drink the so-called JCX_0985 and JCLP_234 with JCLP_045 and raisins (JCLP_200) without the kernels and also add the seeds of stinging nettle (JCLP_211) and water (JCLM_022) and let it boil and afterwards add sugar (JCLP_185) and mastic (JCLP_125) and let him drink. 6. Let him also drink the so-called Armenian lump of earth (JCLM_009), ground, with warm water (JCLM_022) just on an empty stomach. 7. Grind aloe (JCLP_012) and gum ammoniacum (JCLP_015) and add a little myrrh (JCLP_133) and mix it with a little wine (JCLP_099) and let him drink every day on empty stomach in the morning. 8. Let him also drink the antidote that is called theriac (JCX_0301) and the so-called JCX_0964 with a little wine (JCLP_099) in the morning on empty stomach.

148.[121] For the cancerous disposition of the breasts, that is if the breasts of women or men have the disease that the doctors call cancer. Do this treatment for this disease. Take crushed litharge (JCLM_018) and table oil (JCLP_232) and a little vinegar (JCLP_142) and mix it together and apply where the diseases is on the breasts. 2. Take so-called very finely crushed wild cress (JCLP_077) and crushed lead white (JCLM_042) and myrtle oil (JCLP_134) and mix it and apply to the disease of cancer.

149.[121] Once this disease, the cancer, has healed and passed and if you want that her breasts are fine and don’t suffer anything bad like cancer or worse. Do this treatment. Mix finely crushed cumin (JCLP_105) with water (JCLM_022) and macerate and apply a cataplasm onto the breasts that is put it onto the breasts of the woman, where the disease is. Once you put the cumin (JCLP_105) on top of the of cumin (JCLP_105), put a new sponge (JCA_0200) soaked with vinegar (JCLP_142) and wine (JCLP_099) and bind it with cloth (JCLP_151). Once three days have passed, remove the cumin (JCLP_105) and add pounded JCLP_036 softened with honey (JCA_0024) and put it on top of the disease. Bind this JCLP_036 with honey (JCA_0024) for three days. Drink this three times a month.

150.[122] If their armpits are smelling bad and burning. Do this treatment so that they don’t smell bad. That is, crush liquid JCLM_030 and crushed myrrh (JCLP_133) and mix it with wine (JCLP_099) and apply to the armpits. 2. Do the same also for the men if the armpits are smelling bad or sweating. 3. Crushed litharge (JCLM_018) and crushed myrtle (JCLP_134) and what is called by the doctors black cardamom (JCLP_018). Crush it all and mix it with good wine (JCLP_099) and apply to the armpits. 4. Crushed liquid JCLM_030 and myrrh (JCLP_133) and black cardamom (JCLP_018) and what is called by the doctors spikenard (JCLP_136). Pound it all and mix with good spiced (JCX_2243) wine (JCLP_099) and apply to the area of the armpits where the bad smell is.

151.[124] If a person vomits something like fried eggs, it is a sign that there is a strong and warm imbalance in his stomach. Do this treatment. That is let him dip bread into vinegar (JCLP_142) and into mixed water (JCLM_022) and let him eat. Let him also eat very thick sour yoghurt (JCA_0870) and chicory (JCLP_068) with acrid vinegar (JCLP_142) and let him drink instead of wine (JCLP_144) acrid vinegar (JCLP_142) with water (JCLM_022).

152.[125] Those who don’t have an appetite because of freezing, that is from cold that is they are cold, do this treatment. That is let him drink old unmixed wine (JCLP_099) or with warm water and the so-called JCX_2047 wine (JCLP_144) and water (JCLM_036) and let him drink mixed fish (JCA_0526) sauce (JCA_0489). 2. Put water (JCLM_022) into a pan and dill (JCLP_073) and rue (JCLP_157) and the root of parsley (JCLP_120) and let it boil and once it has boiled, then put a little crushed pepper (JCLP_154) into a vessel and let him drink in the morning on empty stomach, the amount of a full cup. Let it be a medium sized cup. Let him also eat this in the evening, that is take honey (JCA_0024) and let it boil and this is made as you make Of Quince (JCLP_103) or the Of Medic Apple (JCLP_088). And take crushed pepper (JCLP_154) and crushed white pepper (JCLP_154) like the one we eat and crushed long pepper (JCLP_154) and crushed white pepper (JCLP_154). This white pepper (JCLP_154) can be found in the pepper (JCLP_154) that we eat. And stir it thoroughly and well. In the evening, he shall not have dinner and let him eat from it about the equal amount of a nut and he shall not chew it a lot at all if possible and there is a way. Let him drink like this. Then let him drink on top of this warm old wine (JCLP_099) and let him sleep. Let him do the same in the evening but do not let him have dinner at all nor let him eat anything else neither before nor after. So that there is no great harm instead of the good, he shall take care not to have dinner. 3. Let him also eat, once this has been completed, what is called by the doctors Of Calamint (JCLP_074). That is take honey (JCA_0024) and let it boil a little bit as I have told you above. Then take the dry leaves of calamint (JCLP_074) that is the so-called JCLP_167 crushed the same way and the leaves of pennyroyal (JCLP_040) and dry parsley (JCLP_155) crushed that is parsley (JCLP_120) and the dry crushed leaves of the so-called thyme (JCLP_066) and a little crushed pepper (JCLP_154) and stir it with honey (JCA_0024) and let him eat it like the other one that I have told you above. Let him also eat what is called by the doctors JCX_1702 that is take honey (JCA_0024) and let it boil as I told you a bit earlier. Then add crushed pepper (JCLP_154) like the one we eat and crushed leaves of rue (JCLP_157) and crushed cumin (JCLP_105) and JCLP_140 and what is called by the doctors nitron (JCLM_023) and mix it with honey (JCA_0024). And stir it well and have it done in the same way as I told you a little bit earlier just make sure that he does not have dinner. 4. Let him drink the antidote that is called Of a Male Calf (JCA_0515) about four with wine (JCLP_099) with the antidote called theriac (JCX_0301) and let him also eat raw and cooked garlic (JCLP_197).

153.[126] For those that have a lot of fluids in the stomach and he is anorexic because of these fluids. Do this treatment. That is let him drink well in the morning JCX_0423 of the quantity of eight kernels with wine (JCLP_099). 2. Make sure that he does not eat anything salted either flesh (JCA_0303) or fishes (JCA_0594) nor drink JCX_1855 nor water nor pulses (JCLP_148). Let him eat once a day and he shall not have a meal. 3. If not let him drink warm old wine (JCLP_099). 3. Plaster for anorexia. Grind dry roses (JCLP_181) and aloe (JCLP_012) and ink-gall (JCLP_085) and ground dates (JCLP_222) and a little vine flower (JCLP_143) and dry ground melilot (JCLP_128) and crushed mastic (JCLP_125) and crushed JCX_0114 and crushed seed of wormwood (JCLP_028) and make it fine and yolk (JCX_0114) of an egg (JCA_0105) and a little resin (JCLP_158) and make a large plaster and put it on the stomach.

154.[127] Dog-like appetite is, if a person yearns for much more to eat and eats and then continues to be hungry and eats again like the dogs eat and always has an appetite. Such is the appetite of the person similar to that of a dog and therefore it is called dog-like appetite because it is similar to the appetite of the dog. This disease arises from a cold humour and through sour humour in the stomach. It is useful for those to drink water (JCLM_036) and mixed fish (JCA_0526) sauce (JCA_0489) and the so-called Of Three Peppers (JCLP_154), of which I have written down the composition for you where it is written on the anorexia through thick humours and JCX_0783 and I have told you how it is made. Let him also eat Of Quince (JCLP_103) in the evening and let him drink on top old warm wine (JCLP_099) and he shall not have dinner.

155. [128] If a person has a hiccup, which the doctors call *lygmon*, but the locals call *louxikas*. Some call it *kloxon*. Do this treatment. That is, boil rue (JCLP_157) with wine (JCLP_099) and let him drink. 2. Let him drink boiled calamint (JCLP_074) and JCLP_167 with wine (JCLP_099) or crushed castor (JCA_0145) with a little vinegar (JCLP_142) and wine (JCLP_144) and let him drink it but it should not boil.

156. If a person has indigestion, it is a sign that he vomits something like acid. Do this treatment. That is, warm fish (JCA_0526) sauce (JCA_0489) and a little water (JCLM_022) and let him drink it on empty stomach and then let him eat. 2. Let him eat Of Quince (JCLP_103) slowly and let him drink warm old wine (JCLP_099) on top of that and he shall not eat anything else. 3. Let him also eat the so-called JCX_0783 and Of Three Peppers (JCLP_154) and Of Calamint (JCLP_074) similarly to Of Quince (JCLP_103). I wrote the same for you a bit earlier on how it is made in when they have anorexia because of thick humours. 4. Let him also eat what is called Of Medic Apple (JCLP_088) in the evening and let him drink old warm wine (JCLP_099) and he shall not have dinner. 5. Let a small child sleep next to the patient, just let it be well fed and fat and thick, so that it warms up the patient. If you do not have a child, let him take fat and thick puppies and let him sleep next to these. If you do not have puppies, the patient shall sleep next to a young unmarried woman just let her be fat and thick like they used to do in the olden days. They had this custom. If there is no unmarried woman, he shall sleep next to a married woman. But one should take good care as far as possible that he shall not have intercourse with the unmarried woman or the other as it would increase the indigestion. If the patient is a woman let a child or an unmarried woman or a puppy sleep with her, just thick. Likewise, let the unmarried woman be thick and fat a lot as well as the child, be it either male or female it should be fat. 6. If the indigestion arises from heat in the hollow of the stomach. It is a sign that he burps something like the smell of grilled meat and he burns inside a lot but he does not evacuate. Someone like this, thus, should either eat cold things like fruit and both unripe and ripe things and chicory (JCLP_068) lettuce (JCLP_119) with acrid vinegar (JCLP_142) and JCX_1262 and very sour and thick yoghurt (JCA_0870). Instead of wine (JCLP_144) let him drink water (JCLM_022) with acrid vinegar (JCLP_142).

157.[129] If a person discharges bile both from above and below with urgency. Do this treatment. That is grind what is called *psalidas* by the common people, and by the doctors tendrils of the vines (JCLP_016) and let him drink their juice. 2. Boil dry roses (JCLP_181) with stern wine (JCLP_099) and also add vine flower (JCLP_143) and let him drink. 3. Grind a sour pomegranate (JCLP_182) and let him drink the juice. 4. Let him also drink stern wine (JCLP_099) and let him eat and crumbs with stern wine (JCLP_099). 5. Let him also eat wheat flour (JCLP_187) with stern wine (JCLP_099) and let it boil. 6. Let him also eat fruit like the so-called medlar (JCLP_130) and unripe quince (JCLP_103) and pears (JCLP_021) and sour pomegranate (JCLP_182). 7. Also rinse his feet with warm water (JCLM_022) in the evening and he shall keep them warm. 8. Grind the kernels of raisins (JCLP_200) and let him drink them with stern wine (JCLP_099). 9. Crush the fruit of bramble (JCLP_032) that is its unripe dry kernels and let him drink with stern wine (JCLP_144). 10. Add vinegar (JCLP_142) and wine (JCLP_099) and the leaves of laurel (JCLP_049) and of myrtle (JCLP_134) and the leaves of mastic (JCLP_209) and broken ink-gall (JCLP_085) and the peel of pomegranate (JCLP_182) which the doctors call pomegranate fruit shell (JCLP_243) and let it boil and apply as a warm poultice on the stomach four times a day each time about ten cloths. Also apply a hot poultice to the anus from below with these. 11. The same treatment that is appropriate for a person who is suffering from freezing that is cold and his stomach is running a lot, but let it be hot. And someone like this shall not drink anything cold nor shall he eat anything cold and if he is not feverish bathe him.

158.[130] For an inflammation of the intestines. If it happens to a person that his intestines (JCX_0794) are inflamed or he vomits stool and it does not find a way to pass through and the stool comes up again and the person vomits it, this disease is called ileus by the doctors. The disease is fatal for some if there is an inflammation of the intestines. If this disease arises from a constipation of stool, it is salvageable. And if the patient is a child that is fourteen years old, do baths and cataplasms and fumigations. If the patient is a young man that is twenty two years old, let his blood. If the patient is at the hight of his life, that is forty two years old, also let his blood and they shall not drink wine (JCLP_099) and let it be hot.

159.[131] If the kidneys are inflamed and there is pain and pressure on the place, where the inflammation of the kidneys is and these people have fever and vomit bile. Let the blood of these people. 2. Grind the kernels of fenugreek (JCLP_212) and of rue (JCLP_157) and the seed of dill (JCLP_073) and macerate with water (JCLM_036) and put it on the area of the kidneys. 3. Let him drink the crushed seed of a pumpkin (JCLP_153) mixed with honey (JCA_0024) and with wine (JCLP_099). On top of the kidneys put a hot poultice of good warm wine (JCLP_099) and good warm cloth (JCLP_151). 4. Apply the plaster that is called Of Gruel, by the doctors JCX_0854 plaster and spread on the cloth (JCLP_151) place it onto the kidneys, where the pain is. 5. Dissolve wax (JCA_0576) and add rose oil (JCLP_181) and yolk (JCX_0114) of an egg (JCA_0105) and macerate them and put on top of the kidneys with cloth (JCLP_151).

160.[132] Diabetes is called, if he urinates as soon as he drinks, and he does this frequently and every hour. Let these drink the juice of polygonum (JCLP_164) or the so-called JCU_2493. Boil it with thick black wine (JCLP_099) and let him drink. 2. Boil dates (JCLP_222) and let him drink the juice. 3. Boil the leaves of myrtle (JCLP_134) with wine (JCLP_099) and let him drink. 4. On top of the kidneys put boiled barley flour (JCLP_100) with water (JCLM_022). Then once it has boiled also add vinegar (JCLP_142) and rose oil (JCLP_181) and boil it, then add pounded tendrils of vine (JCLP_016) and pounded leaves of vine (JCLP_016) and pounded JCLP_078 and put it on the kidneys.

161.[133] Fissures are called by the doctors, if the testicles split or the anus. Useful for these are crushed pine resin (JCLP_177) macerated in rose oil (JCLP_181) and applied to the split areas, but roast the pine resin (JCLP_177) in the sun. Then crush yolk (JCX_0114) of an egg (JCA_0105) put it on top of the wounds or opium (JCLP_145) with JCX_0114. 2. Rub dry leaves of ivy (JCLP_087) onto the wound. 3. Mix ashes (JCX_0126) of the leaves of ivy (JCLP_087) with rose oil (JCLP_181) and put them into a of lead (JCLM_024) vessel and stir it and then apply. But first wipe the wounds. Wine (JCLP_144) instead of water (JCLM_022) but let the wine (JCLP_099) be warm. Then rub crushed dry roses (JCLP_181). 4. Rub crushed lead white (JCLM_042) and litharge (JCLM_018) onto the wounds. If the crotch is inflamed, pound the tender leaves of vine (JCLP_016) and mix them with lead white (JCLM_042) and put them on top on the inflammation. 5. Rinse the crotch first with cold water (JCLM_022) of the sea (JCX_0496). If there is none of the sea (JCX_0496), take salt (JCLM_002) and water (JCLM_022). Then pound JCLP_078 and mix it with lead white (JCLM_042) and yolk (JCX_0114) of an egg (JCA_0105) and put it on the inflammation of the crotch. Crotch is called what we are ashamed to show the others.

162.[134] If someone swallows leeches by mistake, let him drink salt water (JCLM_004) with water (JCLM_022). 2. Mix ground nitron (JCLM_023) with water (JCLM_022) and let him keep it in his mouth. And let him move this water (JCLM_022) back and forth in his mouth. Let him also eat garlic (JCLP_197). 3. Grind JCU_0677 and mix them with acrid vinegar (JCLP_142) and let him drink them.

163.[134] If someone eats a lot of green/fresh mushrooms and almost chokes. Force him to vomit like this, just let him drink olive oil (JCLP_053) and he will vomit. 2. Let him drink tendril (JCLP_090) ashes (JCX_0126) with a lot of vinegar (JCLP_142) and wine (JCLP_099) that is two parts of vinegar (JCLP_142) and one part of wine (JCLP_099). 3. Grind the so-called nitron (JCLM_023) and let him drink it as well as you gave to drink the ashes (JCX_0126) of a tendril (JCLP_090). 4. Boil eggs (JCA_0761) of a bird (JCA_2319) in vinegar (JCLP_142) and wine (JCLP_099) and let him drink boiled. 5. Boil wormwood (JCLP_028) with wine (JCLP_099) and let him drink. 6. Boil melissa (JCLP_129) and ground nitron (JCLM_023) with honey (JCA_0024) and add also a little water (JCLM_022) and let him drink. 7. Boil rue (JCLP_157) with olive oil (JCLP_053) add also wine (JCLP_099) and let him drink. 8. Boil oregano (JCLP_141) with wine (JCLP_099) and honey (JCA_0024) and let him drink. 9. As far as possible, let him vomit, so that he expels it and also bathe him frequently.

164.[135] If the milk (JCX_0085) turns into cheese in the stomach of a person. Let him drink the dry leaves of JCLP_167 with water (JCLM_022) and pound the fresh ones and let him drink their juice. 2. Boil thyme (JCLP_066) with wine (JCLP_099) and let him drink. 3. Rennet (JCA_2401), whatever kind there is, let him drink with vinegar (JCLP_142) just well crushed.

165. If all hair of the head falls out. Do this treatment. That is grind maidenhair (JCLP_004) and mix it with gum ladanum (JCLP_110) and make it like JCX_1263 and put it on top of the entire head. 2. Burn the root of asphodelus (JCLP_027) and macerate the ashes (JCX_0126) with table oil (JCLP_232) and apply to the head. 3. Grind the bark of fresh reed (JCLP_075) and make it smooth with vinegar (JCLP_142) and put it on the head. 4. Mix ground pepper (JCLP_154) with wine (JCLP_099) and vinegar (JCLP_142) and apply to the entire head. 5. Pound the leaves of a wild fig tree (JCLP_207) and of a domesticated fig tree (JCLP_207) and macerate them with honey (JCA_0024). And put them on the head. 6. Burn the skin of a hedgehog/sea urchin (JCA_1087) and mix its ashes (JCX_0126) with vinegar (JCLP_142) and table oil (JCLP_232) and apply to the head. 7. Burn a sea urchin (JCA_1832) just with its shell and mix its ashes (JCX_0126) with vinegar (JCLP_142) and table oil (JCLP_232) and apply to the head. 8. Grind the root of elm tree (JCLP_172) well and mix it with lard (JCA_0077) of a bear (JCA_0435) and apply to the head. 9. Crushed stavesacre (JCLP_003) and sulphur (JCLM_033) and split yellow orpiment (JCLM_006) and mix it with vinegar (JCLP_142) and apply. 10. Mix lead white (JCLM_042) and ground litharge (JCLM_018) with vinegar (JCLP_142) and olive oil (JCLP_053) and apply. 11. Wash lime (JCLM_007) with water (JCLM_022) and pour out the water (JCLM_022) and add old olive oil (JCLP_053) and apply to the head. 12. Shave his whole head well and take vinegar (JCLP_142) and dissolved lard (JCA_0077) of a goose (JCA_0246) and apply. 13. Dissolve lard (JCA_0077) of a goat (JCA_0966) and add cedar oil (JCLP_083) and vinegar (JCLP_142) and apply to the head very calmly. 14. Apply crushed JCX_1560 and mastic (JCLP_125) with vinegar (JCLP_142) to the head. 15. Shave his whole head well and add vinegar (JCLP_142) and dissolved lard (JCA_0077) of a goose (JCA_0246) and apply to the head. 16. Apply mercury (JCLM_037) and saliva (JCA_0904) of a person and spikenard oil (JCLP_136) to the head. 17. Mix dissolved lard (JCA_0077) of a fox (JCA_0624) with vinegar (JCLP_142) and wine (JCLP_099) and apply to the head. 18. Pound beetroot (JCLP_189) and mix their juice with vinegar (JCLP_142) and table oil (JCLP_232) and apply to the head. 19. Mix dry ground mouse (JCA_2443) dung with vinegar (JCLP_142) and table oil (JCLP_232) and apply to the head. 20. Grind the root of celery (JCLP_186) and mix it with vinegar (JCLP_142) and apply to the head. 21. Add rust (JCLM_028) of iron (JCLM_027) and vinegar (JCLP_142) and table oil (JCLP_232) and let it do for one night and one day and apply to the head. 22. The leaves of hyoscyamus (JCLP_217) applied as a cataplasm with vinegar (JCLP_142) but first pound them and crushed pine resin (JCLP_177) with lard (JCA_0077) of a crane (JCA_1646) and marrow (JCA_0307) of a deer (JCA_0366) and vinegar (JCLP_142) and mix everything well and apply. 23. Macerate gum ladanum (JCLP_110) with wine (JCLP_099) and with myrtle oil (JCLP_134) and apply to the head. 24. Pound the tender shoots of mastic (JCLP_209) and macerate them all with rose oil (JCLP_181) and wine (JCLP_099) and put them on the head.

166. On pityriasis of the head. Grind the root of lily (JCLP_101) and apply to the head. 2. Grind the root of a mulberry tree (JCLP_208) and add vinegar (JCLP_142) and macerate and apply to the head. 3. There is a plant called quince (JCLP_203). Grind its root and mix it with vinegar (JCLP_142) and apply. 4. Macerate gum ladanum (JCLP_110) with vinegar (JCLP_142) and apply. 5. Grind the seeds or the leaves of wild cress (JCLP_077) and mix them with vinegar (JCLP_142) and apply. 6. Crush coriander (JCLP_091) and the seed of rue (JCLP_157) or its leaves. And also add vinegar (JCLP_142) and macerate and apply. 7. Pound the leaves of ivy (JCLP_087) and apply their juice to the head. 8. Grind the root of beetroot (JCLP_189) and apply its juice to the head. 9. Pound wild rue (JCLP_157) and also crush litharge (JCLM_018) and pound stavesacre (JCLP_003) and add vinegar (JCLP_142) and myrtle oil (JCLP_134) as much as required and unify these and apply to the head. 10. Mix ground sulphur (JCLM_033) with myrtle oil (JCLP_134) and apply. 11. Grind the root of beetroot (JCLP_189) and apply the juice to the head. 12. Mix crushed litharge (JCLM_018) and salt (JCLM_002) and wine (JCLP_144) and olive oil (JCLP_053) and apply to the head. 13. Add the root of wild cucumber (JCLP_001) and lupine (JCLP_118) and boil these with water (JCLM_022) and rinse with water (JCLM_022). 14. Boil fenugreek (JCLP_212) and also pound the leaves of beetroot (JCLP_189) and mix its juice with water (JCLM_022) of fenugreek (JCLP_212) and apply to the head. 15. Macerate the ashes (JCX_0126) of a tendril (JCLP_090) with water (JCLM_022) and apply.

167.[136] *Alphoi* are called if the person develops something like the kernels of a lentil (JCLP_219) on his face. It is useful for lichen and leprosy. Pound rosemary (JCLP_050) and mix it with cedar oil (JCLP_083) and apply just also add table oil (JCLP_232). 2. Mix crushed sulphur (JCLM_033) and litharge (JCLM_018) and stavesacre (JCLP_003) and table oil (JCLP_232) and apply to the head. 3. Boil fat resinous wood (JCLP_046) with table oil (JCLP_232) and apply. Just also add litharge (JCLM_018) and vinegar (JCLP_142). Apply in the bath. 4. Mix very finely ground marble (JCLM_021) with white (JCX_0101) of an egg (JCA_0105) and apply. 5. Mix lead white (JCLM_042) and dry dung of a swallow (JCA_2853) and white (JCX_0101) of an egg (JCA_0105) and apply. 5. Grind the root of lily (JCLP_101) and mix it with vinegar (JCLP_142) and apply. 6. Grind the root of mulberry (JCLP_208) and macerate it with vinegar (JCLP_142) and apply. 8. Pound the leaves of a wild fig tree (JCLP_207) or also of a domesticated fig tree (JCLP_207) and macerate these with vinegar (JCLP_142) and put them on the location of the disease. 9. Mix crushed stavesacre (JCLP_003) and crushed sulphur (JCLM_033) with vinegar (JCLP_142) and apply. 10. Pound the leaves of JCLP_167 and also grind sulphur (JCLM_033) and add vinegar (JCLP_142) and macerate and apply. 11. Grind JCU_0278 and nitron (JCLM_023) and add vinegar (JCLP_142) and apply.

168. Aphtha is if the mouth of a person has light membranes on the inside and also the gums. 1. Keep rose water (JCLP_181) in the mouth. 2. Boil JCLP_135 with wine (JCLP_144) and let him keep it in his mouth. 3. Let him chew black olives (JCLP_053) of an olive tree (JCLP_053) and let him keep it on his tongue. 4. Boil the peel of pomegranate (JCLP_182) with wine (JCLP_099) and keep in the mouth. 5. Boil stavesacre (JCLP_003) with vinegar (JCLP_142). 6......... let him keep in his mouth. 7. Mix JCLP_135 and rose water (JCLP_181) and honey (JCA_0024) and let him keep it in the mouth. 8. He shall chew the delicate leaves of a wild olive tree (JCLP_053) and he shall apply the juice of the leaves of an olive tree (JCLP_053). 9. Boil the leaves of a wild olive tree (JCLP_053) with vinegar (JCLP_142) and let him keep the juice in his mouth. 10. He shall chew the delicate leaves of bramble (JCLP_032). 11. Let the delicate leaves of bramble (JCLP_032) boil with vinegar (JCLP_142) and let him keep vinegar (JCLP_142) in his mouth. 12. There is JCLP_008 and it is called JCLP_214 and it produces quadrilateral round thorns. Boil the leaves of the thorny plant with rose water (JCLP_181) and apply to the gums.

169. On amblyopia. Pound fresh rue (JCLP_157) and fennel (JCLP_123) and macerate their juice with honey (JCA_0024) and apply to the eyes on the outside. 2. Burn rosemary (JCLP_050) and mix the ashes (JCX_0126) with honey (JCA_0024) and apply to the eyes on the outside. 3. Grind black ivy (JCLP_087) and put the juice into his nose. 4. Drip the juice of JCLP_035 into the inside of the eyes. 5. Apply the bile of geese (JCA_2858) to the eyes externally. 6. Mix crushed litharge (JCLM_018) with vinegar (JCLP_142) and table oil (JCLP_232) and apply to the eyes externally. 7. Mix ground Cappadocian salt (JCLM_002) with the juice of fennel (JCLP_123) and add unsmoked honey (JCA_0024) and mix it and apply it to the eyes externally. 8. Pound fennel (JCLP_123) and apply the juice to the eyes externally. 9. Grind the root of chelidonium (JCLP_231) and mix it with unsmoked honey (JCA_0024) and apply to the eyes externally.

170.[137] It softens joint pain and hardening. Put gum ammoniacum (JCLP_015) one night and one day into acrid vinegar (JCLP_142) and once it gets soft, macerate it with honey (JCA_0024) and put it on top of the location of the joints. 2. Crush pyrethrum (JCLP_173) finely and pepper (JCLP_154) and add table oil (JCLP_232) and unify and apply the painful area. 3. Boil the fresh leaves of JCLP_167 with water (JCLM_022) and then pound these and put them onto the hardening and on the pain of the joints. 4. Mix cedar oil (JCLP_083) with table oil (JCLP_232) and apply onto the pain. 5. Burn the root of ivy (JCLP_087) and mix ashes (JCX_0126) with yolk (JCX_0114) of an egg (JCA_0105) and put it onto the pain. 6. Put crushed, very very fine pepper (JCLP_154) on top of the pain. And put JCU_2779 on top of the pepper (JCLP_154) and burn the area where the pain is. 7. Grind the seed of hyoscyamus (JCLP_217) and make it fine and also add very finely crushed nitron (JCLM_023) and drink it. 8. Apply lard (JCA_0077) of a deer (JCA_0649) or of a bird (JCA_0346) or of a bull (JCA_0466) or of a bear (JCA_0435) to the area where the pain of the joints is.

171.[138] It dissolves and softens an apostema. Macerate pounded leaves of hyoscyamus (JCLP_217) with lard (JCA_0077) of a pig (JCA_0266) and put them on top onto the apostema. 2. Pound hypericum (JCLP_199) and put it on top of the area where the hardening is. 3. Pound the leaves of wild or domesticated monk’s rhubarb (JCLP_111) and boil and put onto the area while warm. 4. Grind linseed (JCLP_116) and make it fine and boil with water (JCLM_022). Then add lard (JCA_0077) of a pig (JCA_0266) and macerate and put it on top onto the apostema. 5. Boil the leaves of mallow (JCLP_132) with water (JCLM_022) then pound them and put them on top of the area. 6. Boil the flour of darnel (JCLP_006) with sweet water (JCLM_022), then add lard (JCA_0077) of a pig (JCA_0266) and put it on top of the wound. 7. Boil barley (JCLP_100) flour and fine linseed (JCLP_116) with water (JCLM_022). Then add lard (JCA_0077) of a pig (JCA_0266), of a goose (JCA_0246) of a bird (JCA_0346), marrow (JCA_0307) of a deer (JCA_0366), lard (JCA_0077) of a bull (JCA_0466), of a panther (JCA_2364), of a bear (JCA_0990), of a male calf (JCA_0515), of a lion (JCA_0839), of a raven (JCA_1160), of a fox (JCA_0624), all dissolved, and add them and macerate and put it on top with a cloth (JCLP_151). 8. Take dung (JCA_0254) of a pigeon (JCA_0882) and vinegar (JCLP_142) and macerate and put it on top with a cloth (JCLP_151) onto the wound.

172.[139] It softens aporyphas and ekbata. Mix finely ground JCU_0278 with propolis (JCA_0710) and put it on top. 2. Boil barley flour (JCLP_100) with the juice of beetroot (JCLP_189) and macerate well and apply them. 3. Pound the leaves of the plant mullein (JCLP_221) and put them on top. 4. Boil onion (JCLP_102) on coals (JCLM_015) and pound it and put it on top. 5. Pound onion (JCLP_102) and make it fine and apply as a cataplasm. 6. Grind the root of monk’s rhubarb (JCLP_111) and macerate with lard (JCA_0077) of a goose (JCA_0246) and of a pig (JCA_0266) and put it on top. 7. Crush resin (JCLP_158) and sprinkle it on top.

173.[140] If someone is bitten by a snake or a viper. Do this treatment. Pound the leaves of an apple tree (JCLP_131) and drink their juice with wine (JCLP_144). Also pound the leaves and put them onto the wound. 2. Pound the leaves of myrtle (JCLP_134) and put them on top of the wound. 3. Split a live JCA_1576 and put it onto the wound. 4. Pound mint (JCLP_063) and drink with warm water (JCLM_022). 5. Pound the leaves of JCLP_096 and put them onto the wound. 6. Crush all heal (JCLP_224) and mix it with table oil (JCLP_232) and apply to the area. 7. Grind the root of JCLP_051 and the leaves and mix them with lard (JCA_0753) of a deer (JCA_0649) and apply. 8. Apply dissolved lard (JCA_1490) of a bear (JCA_0435) onto the wound.

174. If a person has indigestion. Let him drink dry crushed pennyroyal (JCLP_040) with wine (JCLP_099). 2. Grind crushed pepper (JCLP_154) and parsley (JCLP_120) seeds and crush it all together and let him drink it with fish (JCA_0526) sauce (JCA_0489) in the bath if it is coming along well. Make sure that he does not bathe if he evacuates the indigestion. 3. Let him drink crushed cinnamon (JCLP_086) and crushed rue (JCLP_157) seed together with warm water (JCLM_022). 4. Pound hyssop (JCLP_216) and let him drink it with water (JCLM_022).

175. Therapy for cough. Grind the skin of carrot (JCLP_048) and let him drink it with wine (JCLP_144). 2. Grind gum of balsamodendrum (JCLP_034) and let him drink with wine (JCLP_144). 3. There is a plant called JCLP_051. Let him eat its root raw and boiled. 4. Let him eat the raw root of JCLP_051 with honey (JCA_0024). 5. Pound the leaves of rue (JCLP_157) and let him drink the juice. 6. Grind Pontic sumach (JCLP_175) and let him drink with water (JCLM_036). 7. There is a plant called polygonum (JCLP_164). Pound it and let him drink the juice. 8. Pound green wormwood (JCLP_028) and let him drink the juice. 9. Boil maidenhair (JCLP_004) with water (JCLM_022) and let him drink it with wine (JCLP_099). 10. Grind the root of white JCLP_008 and let him drink with water (JCLM_036). 11. Grind gum ammoniacum (JCLP_015) and make it fine and let him drink it with honey (JCA_0172) or with the juice of JCLP_045. 12. Boil purslane (JCLP_019) that is called purslane (JCLP_041) with vinegar (JCLP_142) and wine (JCLP_099) and let him eat it. 13. Put propolis (JCA_0710) on live coals (JCX_0499) and fumigate him. 14. Make green pine resin (JCLP_177) into kernels like chickpeas (JCLP_055) and let him swallow them with warm water (JCLM_022). 15. Fumigate him from below with lotus (JCLP_042). 16. Put yellow orpiment (JCLM_006) and all heal (JCLP_224) onto burning coals (JCX_0499) and fumigate him and let him swallow the smoke. 17. Put resin (JCLP_158) and frankincense (JCLP_114) and storax (JCLP_206) onto burning coals (JCX_0499) and fumigate. 18. Grind the fruit of JCLP_030 and make it fine and let him drink it with water (JCLM_036). 19. Let him eat the green shoots of cypress (JCLP_106) with vinegar (JCLP_142). 20. Boil rosemary (JCLP_050) with wine (JCLP_144) and let him drink it in the morning. 21. Boil the root of fennel (JCLP_123) with wine (JCLP_099) and let him drink. 22. Grind the seed of fennel (JCLP_123) and let him drink with wine (JCLP_144). 23. Boil the leaves of a pine (JCLP_156) and its tender twigs with wine (JCLP_144) and let him drink. 24. Grind the seed of hyoscyamus (JCLP_217) and let him drink it with water (JCLM_022). 25. Boil hyssop (JCLP_216) and rue (JCLP_157) with honey (JCA_0024) and wine (JCLP_099) and let him drink. 26. Boil the root of mullein (JCLP_221) with wine (JCLP_144) and let him drink. 27. Make fatty storax (JCLP_206) into kernels like of a chickpea (JCLP_055) and let him swallow it with wine (JCLP_099). 28. Make all heal (JCLP_224) into kernels and with white (JCX_0101) of an egg (JCA_0105) let him swallow these.

176.[141] It brings down the milk of women and rushes down menstruation. Grind the seed of agnus castus (JCLP_115) and let her drink with wine (JCLP_144). 2. Boil the root of dill (JCLP_073) and let her drink. 3. Boil the seed of dill (JCLP_073) with water (JCLM_022) and let her drink the root of fennel (JCLP_123) and the leaves and the seed of ......... and let her drink. 4. Boil the shoot of dill (JCLP_073) or its seed with water (JCLM_022) let her drink. 5. Boil the seed, root and the leaves of fennel (JCLP_123) with wine (JCLP_144) and let her drink. 6. Grind nigella (JCLP_126) and let her drink with wine (JCLP_144). 7. Grind basil seed (JCLP_031) and let her drink it with water (JCLM_022). Next boil a cabbage (JCLP_098) and let her eat it. 8. Let her eat the boiled leaves of JCLP_174 and its tender shoots. 9. Rinse frequently with salt water (JCLM_004) of the sea (JCLM_011). 10. Pound JCLP_038 and apply its juice to the breasts of the woman and then sprinkle lead white (JCLM_042) on top. 11. Grind the leaves of hyoscyamus (JCLP_217) and apply their juice to the breasts. 12. Mix vinegar (JCLP_142) and water (JCLM_022) and salt (JCLM_002), then add lead white (JCLM_042) and ground litharge (JCLM_018) and mix it all and apply to the breasts.

177.[142] If a woman can’t give birth quickly. Take river crabs (JCA_0667) and put them on live coals (JCX_0499) and fumigate her and she will immediately give birth.

178.[143] It softens roughness of the tongue. That is boil honey (JCA_0024) and keep fine cloth (JCLP_151) on top which has inside linseed (JCLP_116) and once the cloth (JCLP_151) starts to sweat from the vapour of the honey (JCA_0172) then rub it onto the tongue. 2. Pound fresh mint (JCLP_063) and make it fine and rub it onto the tongue. 3. Boil dried figs (JCLP_071) with water (JCLM_022) and the water (JCLM_022) of the dried figs (JCLP_071), let him keep it in his mouth. 4. Pound fresh mint (JCLP_063) and remove the juice and mix it with rose oil (JCLP_181) and chamomile (JCLP_227) and apply to the tongue. 5. Pound fresh mint (JCLP_063) and apply to the head. 6. Boil green mint (JCLP_063) with water (JCLM_022) and a little wine (JCLP_099) and keep this liquid in the mouth. 7. Chew the leaves of an olive tree (JCLP_053), keep it on the tongue.

179. Therapy for dysuria. Boil wormwood (JCLP_028) with water (JCLM_022) and when it is boiling, mix it with wine (JCLP_099) and let him drink. 2. Boil asparagus (JCLP_026) with water (JCLM_022) and let him drink it. 3. Boil JCA_1310 with water (JCLM_022) and let him drink. 4. Grind JCU_0677 well and let him put them in front of the opening of the crotch. 5. Boil asparagus (JCLP_026) with water (JCLM_022) and let him drink it. 6. Grind the kernels of wild and domesticated grapes (JCLP_237) and drink with wine (JCLP_144). 7. Grind the seed of the plant clover (JCLP_215) and drink with wine (JCLP_144). 8. Boil JCLM_039 with wine (JCLP_144) and drink. The doctors call it *pyrites* (JCLM_026) stone (JCLM_019). 9. Pound a stone (JCLM_019) which is found in new sponges (JCA_2623) thoroughly and drink it with wine (JCLP_144). 10. Boil rosemary (JCLP_050) with wine (JCLP_144) and drink it. Take the root of chicory (JCLP_089), the bark of it, and the root of celery (JCLP_186) and the root of parsley (JCLP_120) and the root of fennel (JCLP_123) and the root of chicory (JCLP_068) and of dill (JCLP_073) and of wild and domesticated rue (JCLP_157) and let it boil with water (JCLM_022) and let him drink their juice. To these also add the root of a radish (JCLP_176) and of sowthistle (JCLP_062).

180. On dysentery. Pound JCLP_078 let him drink the juice. 2. Crush myrrh (JCLP_133), the amount of a bean (JCLP_218), and let him drink it with wine (JCLP_144). 3. Boil the leaves of agnus castus (JCLP_115) with wine (JCLP_144) and let him drink. 4. Boil the leaves and shoots of bramble (JCLP_032) with wine (JCLP_099) and let him drink. 5. There is a plant called ground pine (JCLP_228). Boil it with wine (JCLP_144) and let him drink in the morning with wine (JCLP_099).

181. If he has pain in the ear and does not hear. Do this treatment. That is grind nuts (JCLP_079) and their oil (JCLP_053) or that of sweet almonds (JCLP_017). Make it warm and drip it into the ear. 2. Drip JCLP_030 into the ear. 3. Drip the bile of a hare (JCA_0453) with milk (JCX_0085) of a woman (JCX_0118) into the ear. 4. Mix the bile of a raven (JCA_1160) with a good new honey (JCA_0024) and drip it into the ear. 5. Drip warm juice of an onion (JCLP_102) into the ear. Pound the root of asphodelus (JCLP_027) and drip the juice into the ear.

182. If the menstruation of a woman is late. Do this treatment. That is let her drink the amount of six kernels of JCO_1382 in the morning with water (JCLM_022). 2. Boil maidenhair (JCLP_004) with water (JCLM_022) and let him drink in the morning. 3. Pound the leaves of stinging nettle (JCLP_211) and pound myrrh (JCLP_133) and mix it up and give it to another woman and let her put it into her womb of let her put it outside of her ........ 4. Pound hazelwort (JCLP_025) and let her drink it with honey (JCA_0024). 5. Pound the root of asphodelus (JCLP_027) and let her drink its juice with wine (JCLP_099).

183.[184] If it happens to a pregnant woman that the child dies inside the womb. Do this treatment, so that it is expelled. Let her drink boiled wormwood (JCLP_028) with wine (JCLP_099). 2. Pound wormwood (JCLP_028) and macerate with honey (JCA_0024) and put it inside of her body. 3. Let her drink carrot (JCLP_048) grown somewhere without having sown its seed with wine (JCLP_144). 4. Grind the tear of a wild olive tree (JCLP_053) and let her drink it with wine (JCLP_144). 5. Grind the seed of JCLP_051 and let her drink it with honey (JCA_0024) and wine (JCLP_099) in the morning. 6. Boil the bark of capers (JCLP_076) or its root with wine (JCLP_144) and let her drink. 7. Let her drink fine cinnamon (JCLP_086) and fine myrrh (JCLP_133). 8. Boil the root of cyclamen (JCLP_104) with water (JCLM_022) and let her drink. 9. Grind the seed of parsley (JCLP_120) and let her drink it with water (JCLM_022). 10. There is a plant called clover (JCLP_215). Boil its leaves and the flowers and the seed with water (JCLM_022) and let her drink. 11. Boil oregano (JCLP_141) with water (JCLM_022) and let her drink. 12. Boil the root of elm tree (JCLP_172) with honey (JCA_0024) and wine (JCLP_099) and let her drink. 12. There is a plant called JCLP_241. Boil it with water (JCLM_022) and let her drink.

184.[145] If someone is bitten by a snake or a viper. Grind the root of asphodelus (JCLP_027) and let him drink it boiled with wine (JCLP_099). 2. Grind the root of asphodelus (JCLP_027) well and put it on top onto the wound. 3. Let him drink crushed castor (JCA_0145) with wine (JCLP_144). 4. Grind the leaves of lily (JCLP_101) and put them on top of the wound. 5. Grind carob (JCLP_139) well and let him drink with wine (JCLP_144).

185. It treats and expels what is called *ermyngia* by the common people, by the doctors worms. That is pound JCLP_078 and give its juice to drink with wine (JCLP_144). 2. Let him drink wormwood (JCLP_028) and the bile of a bull (JCA_1325), just boil the wormwood (JCLP_028) first with water (JCLM_022) then mix it and let him drink. 3. Grind bitter lupine (JCLP_118) and mix their flour with honey (JCA_0024) and let him eat. 4. Boil summer savory (JCLP_065) with water (JCLM_022) and let him drink. 5. Boil JCLP_098 with water (JCLM_022) and let him drink. 6. Grind the seed of cabbage (JCLP_098) and let him drink it with water (JCLM_022). 7. Boil the root of a mulberry tree (JCLP_208) with water (JCLM_022) and let him drink in the morning. 8. Boil the root of elm tree (JCLP_172) with honey (JCA_0024) and let him drink just add also wine (JCLP_099). 9. Soak the root of polypodium (JCLP_165) in syrup (JCO_0157) and eat it. 10. Boil hyssop (JCLP_216) with water (JCLM_022) and wine (JCLP_099) and let him drink it in the morning. 11. Grind the seed of a radish (JCLP_176) and let him drink them with water (JCLM_022). 12. Let him eat garlic (JCLP_197).

186. If a person is suffering from the so-called sciatica. Let him drink crushed gum ammoniacum (JCLP_015). 2. Put gum ammoniacum (JCLP_015) into vinegar (JCLP_142) in the evening and let it boil. And once it is soft spread it into a cloth (JCLP_151) and put it on top of the location where it hurts. 3. Boil hazelwort (JCLP_025) with water (JCLM_022) and let him drink. 4. Boil hazelwort (JCLP_025) and honey (JCA_0024) and wine (JCLP_099) and let him drink it with water (JCLM_022). 5. Boil hyssop (JCLP_216) with honey (JCA_0024) and water (JCLM_022). Let him drink it in the morning. 6. Boil the root of asparagus (JCLP_026) with wine (JCLP_099) and let him drink. 7. Boil the seed of capers (JCLP_076) with wine (JCLP_099) and let him drink. 8. Boil the bark of the root of capers (JCLP_076) with wine (JCLP_099) and let him drink. 9. Boil centaury (JCLP_084) with wine (JCLP_099) and let it be given with a clyster. 10. Boil rue (JCLP_157) with olive oil (JCLP_053) ... and let him drink.

187.[146] If his face gets yellow, which is called icterus by the doctors, by the locals chrysiasmos, do this treatment. Boil maidenhair (JCLP_004) with wine (JCLP_099) and let him drink. 2. Let him drink crushed aloe (JCLP_012) with water (JCLM_022). 3. Boil the root of asphodelus (JCLP_027) with wine (JCLP_099) and let him drink and he shall eat its shoots instead of vegetables. 4. Boil the tip of wormwood (JCLP_028) with water (JCLM_022) or with wine (JCLP_099) and let him drink. 5. Boil a centipede (JCA_2591) with water (JCLM_022) and let him drink. 6. Grind the seed of atriplex (JCLP_233) and let him drink it either with water (JCLM_022) or with yoghurt (JCA_2760). 7. Boil the root of monk’s rhubarb (JCLP_111) with wine (JCLP_099) and let him drink. 8. Boil chamomile (JCLP_227) and let him drink the decoction. 9. Boil the root of chelidonium (JCLP_231) with wine (JCLP_144) and let him drink. 10. Boil the root of cyclamen (JCLP_104) with honey (JCA_0024) and water (JCLM_022) and let him drink.

188. On headache. Grind the leaves of agnus castus (JCLP_115) and its seed and mix it with rose oil (JCLP_181) and apply to the head. 2. Pound JCLP_078 and mix its juice with rose oil (JCLP_181) and apply to the head. 3. Mix crushed aloe (JCLP_012) with rose oil (JCLP_181) and vinegar (JCLP_142) and apply to the forehead and the temples. 4. Grind bitter almonds (JCLP_017) well and mix them with vinegar (JCLP_142) and rose oil (JCLP_181) and apply to the forehead and the temples. 5. Grind bitter almonds (JCLP_017) and mix it with vinegar (JCLP_142) and rose oil (JCLP_181) and apply to the forehead and the temples. 6. Pound fresh leaves of vine (JCLP_016) and what is called by the common people tendrils, and by the doctors *helikes* of vine (JCLP_016). Pound these and mix also the leaves. And put it onto the part of the head where the pain is. 7. Pound fresh leaves of cabbage (JCLP_098) and put their juice into the nose, just warm. 8. Pound green mint (JCLP_063) and put it onto the forehead. 9. There is a plant called fleabane (JCLP_095). Pound its leaves and mix them with table oil (JCLP_232) and apply to the head. 10. Apply also its leaves. Grind fleawort (JCLP_235) and mix it with rose oil (JCLP_181) and vinegar (JCLP_142) and apply to the head. There is a plant called JCLP_038. By the doctors, it is called JCLP_204. Pound this and mix the juice with table oil (JCLP_232) and apply to the head. 12. Grind nigella (JCLP_126) and mix it with table oil (JCLP_232) and vinegar (JCLP_142) and apply to the head. 13. Grind mustard (JCLP_192) and macerate with honey (JCA_0024) and put it onto the forehead. 14. Boil domesticated rue (JCLP_157) with vinegar (JCLP_142) and rose oil (JCLP_181) and apply to the head and the forehead and the temples.

189. [147] If his stomach runs. Do this treatment so that it stops. Take the root and the shoots of bramble (JCLP_032) and grind these and let him drink their juice. 2. Grind the root of an oak (JCLP_171) and boil it with wine (JCLP_099) and let him drink. 3. Boil the root and the leaves of JCLP_150 with wine (JCLP_099) and let him drink. 4. Boil the leaves of myrtle (JCLP_134) with wine (JCLP_099) and treat with a clyster. 5. Boil the seed of mastic (JCLP_209) with wine (JCLP_099) and treat with a clyster. 6. Boil the seed of mastic (JCLP_209) and its tender leaves with water (JCLM_022) and let him drink. 7. Boil gourd (JCLP_092) with wine (JCLP_099) and let him drink with wine (JCLP_099). 8. Boil the tender shoots of cypress (JCLP_106) with wine (JCLP_099) and let him drink. 9. Boil the leaves of JCLP_236 with wine (JCLP_099) and let him drink. 10. There is a plant called plantain (JCLP_023), grind its seed and let him drink it with water (JCLM_036).

190.[148] For if someone is bitten by a dog. Do this treatment. That is pound melissa (JCLP_129) and let him drink with wine (JCLP_144). 2. Pound the leaves of JCLP_167 and mix them with ashes (JCX_0126) and put them on the wound. 3. Grind garlic (JCLP_197) and put it on top. 4. Grind garlic (JCLP_197) and let him drink it with water (JCLM_022). And let him also eat garlic (JCLP_197). 5. Pound rue (JCLP_157) and mix it with table oil (JCLP_232) and put it on the wound. 6. Pound tender leaves of a wild fig tree (JCLP_207) and macerate with honey (JCA_0024) and put it on top of the wound. 7. Pound polygonum (JCLP_164) and let him drink the juice with wine (JCLP_144).

191. For a head cold. Do this treatment. Grind white dry dung (JCA_0254) of a dog (JCA_0920) and mix it with honey (JCA_0024) and put it onto the neck. 2. Put cedar oil (JCLP_083) onto the neck. 3. Warm up honey (JCA_0024) and water (JCLM_022) and let him keep it in his mouth. 4. Boil bran (JCLP_160) with water (JCLM_022) and syrup (JCO_0157) then sift it and let him keep the juice in the mouth and let him keep it a long time.

192. Treat the so-called *louxikan*, called by the doctors hiccup, like this. Pound fresh mint (JCLP_063) and mix its juice with the juice of sour pomegranate (JCLP_182) and let him drink. 2. There is a plant called scolopendrium officinale (JCLP_196). Boil it with water (JCLM_022) and let him drink. 3. Pound aristolochia (JCLP_022) and let him drink with warm water (JCLM_036). 4. There is a plant called madwort (JCLP_013). Boil it with water (JCLM_022) and let him drink.

193.[149] If someone is bitten by a rabid dog. Do this treatment. That is, there is a plant called gentian (JCLP_039). Boil its root with wine (JCLP_144) and let him drink. 2. Burn river crabs (JCA_0667) over burning coals (JCX_0499) and let him drink the ashes (JCX_0126) of the crabs (JCA_1919) with wine (JCLP_144). 3. Grate the so-called stone (JCLM_019) hematite (JCLM_001) on a whetstone and collect it. Let him drink it with wine (JCLP_144). 4. Gum of balsamodendrum (JCLP_034) drunk with wine (JCLP_144).

194. The stones that are in the bladder and the kidneys. These expels the resin of vine (JCLP_016), let him drink this with wine (JCLP_144). 2. Boil the root of monk’s rhubarb (JCLP_111) with wine (JCLP_144) and let him drink. 3. Let him drink the tear of an almond tree (JCLP_017) with vinegar (JCLP_142) and with broth. 4. Grind the root of asparagus (JCLP_026) and let him drink the juice. 5. Grind the root and leaves of scolopendrium officinale (JCLP_196) and let him drink. 6. Grind the bark of the root of capers (JCLP_076) and let him drink with wine (JCLP_144). 7. Grind liquorice (JCLP_043) and let him drink the juice with wine (JCLP_144). 8. Grind the root of quince (JCLP_203) and let him drink the juice with wine (JCLP_144). 9. Grind the root of periwinkle (JCLP_225) and let him drink the juice with wine (JCLP_144).

195. Therapy for lichen and leprosy. There is a plant called JCLP_033. Pound its leaves and put them there. 2. Put the tear of a black of an olive tree (JCLP_053) in vinegar (JCLP_142) and let it soak one night and one day and then apply. 3. Burn the so-called mussel (JCA_2034) and mix the ashes (JCX_0126) with vinegar (JCLP_142) and apply. 4. Pound the leaves of damson (JCLP_047) and mix them with vinegar (JCLP_142) and apply. 5. Let the tear of damson (JCLP_047) soak in acrid vinegar (JCLP_142) and then apply. 6. Grind the root of JCLP_051 and mix it with vinegar (JCLP_142) and apply. 7. Grind JCU_0278 and the leaves of rosemary (JCLP_050) and add olive oil (JCLP_053) and liquid pitch (JCLP_244) and vinegar (JCLP_142) and mix and apply. 8. Boil cedar oil (JCLP_083) with vinegar (JCLP_142) and apply. 9. Grind nigella (JCLP_126) and mix it with vinegar (JCLP_142) and apply. 10. Grind the root of wild cress (JCLP_077) and apply with vinegar (JCLP_142). 11. Crush sulphur (JCLM_033) and pine resin (JCLP_177) and nitron (JCLM_023) and add vinegar (JCLP_142) and table oil (JCLP_232) and apply. This also helps for an itch of the genital parts.

196. If the breasts of a woman or a man have hard areas. Pound the leaves of agnus castus (JCLP_115) and mix them with butter (JCA_0554) and put on top with cloth (JCLP_151). 2. Boil the root of asphodelus (JCLP_027) and the leaves and the flowers, boil and let him eat with vinegar (JCLP_142). 3. Pound the leaves of vine (JCLP_016) and mix them with butter (JCA_0554) and put them on top. 4. Grind the root of marsh mallow (JCLP_011) well and put it on top. 5. Pound fresh mint (JCLP_063) and put it on top.

197. It inhibits the breasts of women and the testicles of children so that they do not get big. Pound the leaves of hyoscyamus (JCLP_217) and put them on top. 2. Pound the leaves of mandrake (JCLP_122) and put them on top. 3. Make litharge (JCLM_018) and lead white (JCLM_042) fine and mix with vinegar (JCLP_142) and apply. 4. Boil the leaves of beetroot (JCLP_189) with water (JCLM_022). And then pound them and put them on top. 5. Grind cumin (JCLP_105) finely and mix the flour with fine cereal (JCLP_194) and honey (JCA_0024) and macerate and apply as a cataplasm. 6. Grind rosemary (JCLP_050) and mix it with rose oil (JCLP_181) and put it on top. 7. Grind linseed (JCLP_116) and make it fine and fenugreek (JCLP_212) and mix it. Macerate with rose oil (JCLP_181) and put it on top. 8. Pound the leaves of mallow (JCLP_132) and monk’s rhubarb (JCLP_111) and mix them with rose oil (JCLP_181) and apply as a cataplasm. 9. Mix of bean (JCLP_218) flour with honey (JCA_0024) and put it on top onto the breasts.

198. [198] If someone is bitten by a bee or a wasp. Do this treatment. Pound the leaves of wild mallow (JCLP_132) and put them onto the bite. 2. Pound the leaves of domesticated mallow (JCLP_132) and put it there on the location. 3. Pound the tender leaves of laurel (JCLP_049) and put them on top. 4. Pound the leaves of melissa (JCLP_129) and put them on the wound. 5. Pound fresh rue (JCLP_157) and put it on the wound.

199.[199] If his nose smells. Do this treatment. Pound the leaves of ivy (JCLP_087) and warm up the juice and drip it into the nose. 2. Pound the leaves of JCLP_167 and warm up the juice and drip it into the nose. 3. Pound fresh oregano (JCLP_141) and drip the juice into the nose. 4. Grind nigella (JCLP_126) and mix it with table oil (JCLP_232) and apply onto the outside of the nose and drip it into the inside. 5. Myrrh (JCLP_133), and mix it with milk (JCX_0085) of a woman (JCX_0118) and drip it into the nose.

200.[152] If they beat each other and develop wounds. Do this treatment. That is pound the shoots of a plane tree (JCLP_161) and macerate with lard (JCA_0077) of a pig (JCA_0266) and apply to the wound. 2. Grind the socalled Lemnian earth (JCLM_017) and crush and macerate with water (JCLM_022) and apply. 3. Crush lump of earth (JCLM_009) and macerate with water (JCLM_022) and put it onto the wound. 4. Pound fresh mint (JCLP_063) and macerate with lard (JCA_0077) of a pig (JCA_0266) and apply. 5. Pound the tender leaves of mastic (JCLP_209) and put them on top. 6. Grind the tender leaves of bramble (JCLP_032) and put them on the wound.

201. On infertility. Divide a JCU_1629 in half and take it with your left hand when there is neither sun nor moon and bind it into a linseed (JCLP_116) cloth (JCLP_151) and carry it and you will be astonished. 2. ...... the seed of hyoscyamus (JCLP_217) with milk (JCX_0638) of a horse (JCA_1908), put it into the skin of a deer (JCA_1761) and have it bound onto the arm and you will be astonished. 3. Put the plant ironwort (JCLP_191) into the skin of a deer (JCA_0793) and hand it over and let her carry it.

202. If he vomits a lot even though he does not want to vomit. Do this treatment. Pound fresh mint (JCLP_063) and let him drink the juice with vinegar (JCLP_142) and wine (JCLP_099). 2. Pound a sour pomegranate (JCLP_182) and let him drink the juice. 3. Pound the tendrils of vine (JCLP_016) and let him drink the juice. 4. Roast lentils (JCLP_219) and let him eat it with honey (JCA_0024). 5. Let him eat roasted vetch (JCLP_146) with honey (JCA_0024).

203.[153] For a cloud of the eyes. Do this treatment. Pound JCLP_051 and apply its juice to the eyes externally. 2. Crush cinnamon (JCLP_086) well and mix it with milk (JCX_0085) of a woman (JCX_0118) and apply to the outside of the eye. 3. Pound clover (JCLP_215) and mix its juice with honey (JCA_0024) and apply to the outside of the eye. 4. Crush gum ammoniacum (JCLP_015) and macerate with milk (JCX_0085) of a woman (JCX_0118) and apply to the outside of the eyes. 5. Crush myrrh (JCLP_133) and mix it with new honey (JCA_0024) and apply to the eyes. 6. Pound the leaves of fennel (JCLP_123) and put the juice into a bronze/copper (JCLM_040) vessel and let it be three days then take it out and apply to the eyes every hour and in the morning. 7. Pound the leaves of bramble (JCLP_032) and apply the juice to the eyes. 8. Macerate gum ammoniacum (JCLP_015) with milk (JCX_0085) of a woman (JCX_0118) and apply to the outside of the eyes.

204. For pain of the eyes. Pound the leaves of a wild olive tree (JCLP_053) and apply the juice to the eyes. 2. Pound JCLP_078 and put it on top of the eyes. 3. Pound the leaves of purslane (JCLP_041) and mix them with bran (JCLP_160). Put it into the eyes. 4. Pound wormwood (JCLP_028) and make it smooth with honey (JCA_0024) and put it on top of the eyes. 5. Crush gum ammoniacum (JCLP_015) and unify with milk (JCX_0638) of a woman (JCA_1036) and apply to the eyes. 6. Pound the leaves of fennel (JCLP_123) and put the juice into a bronze/copper (JCLM_040) vessel and leave it for three days. Then take it out and apply to the eyes every hour and in the morning. 7. Pound the tender leaves of bramble (JCLP_032) and put them on top of the eyes. 8. Pound cyclamen (JCLP_104) and apply the juice with honey (JCA_0024) to the eyes. 9. Pound rosemary (JCLP_050) and macerate with honey (JCA_0024) and milk (JCX_0085) of a woman (JCX_0118) and apply to the eyes. 10. Pound mandrake (JCLP_122) and make the juice soft with bran (JCLP_160) or with barley flour (JCLP_100) and put it on the eyes. 11. Grate fresh gourd (JCLP_092) and pound the pieces and mix the juice with bran (JCLP_160). And macerate and put it on top onto the eyes. 12. Pound celery seed (JCLP_186) and mix its juice with bran (JCLP_160) and put it on the eyes. 13. Grind myrrh (JCLP_133) and mix it with honey (JCA_0024) and apply to the eyes. 14. Pound the leaves of hyoscyamus (JCLP_217) and mix them with bran (JCLP_160) and put them on the eyes. 15. Pound fresh roses (JCLP_181) and mix their juice with milk (JCX_0085) of a woman (JCX_0118) and apply. 16. Pound rue (JCLP_157) and mix its juice with bran (JCLP_160) and put it on the eyes. 17. Pound JCLP_078 and mix the juice with honey (JCA_0024) and apply to the eyes. 18. Pound mint (JCLP_063) and mix the juice with milk (JCX_0085) of a woman (JCX_0118) and apply. 19. Pound the tender leaves of bramble (JCLP_032) and mix the juice with with honey (JCA_0024) and milk (JCX_0085) of a woman (JCX_0118) and apply. 20. Grind fleawort (JCLP_235) and mix it with honey (JCA_0024) and milk (JCX_0085) of a woman (JCX_0118) and apply to the eyes. 21. Pound the tender leaves of an olive tree (JCLP_053) and mix them with honey (JCA_0024) and milk (JCX_0085) of a woman (JCA_1034) and apply to the eyes. 22. Pound the fresh leaves of reed (JCLP_075) and apply the juice to the eyes. Put the ground leaves on top.

205.[154] For tooth ache. Do this treatment. Boil black JCU_0278 with vinegar (JCLP_142) and let him keep the vinegar (JCLP_142) where the pain is. 2. Boil the root of asparagus (JCLP_026) with vinegar (JCLP_142) and keep it in the mouth. 3. Grind the root of asphodelus (JCLP_027) and drip the juice into the nostril of the part that hurts. 4. Boil stavesacre (JCLP_003) with vinegar (JCLP_142) and honey (JCA_0172) and let him keep it in the mouth. 5. Boil the tender shoots of a plane tree (JCLP_161) with vinegar (JCLP_142) and keep it in the mouth. 6. Boil the leaves of bramble (JCLP_032) with vinegar (JCLP_142) and keep it in the mouth.

206.[155] If the teeth are wobbly. Do this treatment. Let him bind the root of wild cress (JCLP_077) on his neck. 2. Boil hyssop (JCLP_216) with vinegar (JCLP_142) and let him keep it in his mouth. 3. Boil the bark of a mulberry tree (JCLP_208) with vinegar (JCLP_142) and keep it in the mouth. 4. Let him chew the root of henna (JCLP_149). 5. There is a tree called manna ash (JCLP_127), pound its leaves and let him keep them in the mouth. 6. Let the fruit of capers (JCLP_076) boil with vinegar (JCLP_142) and let him keep it in the mouth. 7. Let the bark of the root of capers (JCLP_076) boil with vinegar (JCLP_142) and let him keep it in the mouth. 8. Boil the leaves of pomegranate (JCLP_182) with vinegar (JCLP_142) and let him keep it in the mouth. 9. Boil the leaves of a wild olive tree (JCLP_053) with vinegar (JCLP_142) and keep it in the mouth.

207.[156] If a person burns a part of the body. Pound plantain (JCLP_023) and put it on top of the burn. 2. Grind JCLP_078 put it on top. 3. Boil the leaves of a mulberry tree (JCLP_208) with olive oil (JCLP_053). Then chop the leaves into small pieces and put them on top.

208.[157] For people with pain in the feet the root of JCLP_036 is useful. Grind it and put it where the pain is. 2. Grind the root of lily (JCLP_101) and put it where the pain is. 3. Grind the root of turnip (JCLP_044) and put it on top where the pain is. 4. Boil the seed of turnip (JCLP_044) with water (JCLM_022) and water (JCLM_022) rinse the feet with it. 5. Grind the root of a radish (JCLP_176) and apply it to the place. 6. Boil the seed of a radish (JCLP_176) with water (JCLM_022) and rinse the feet with this same water (JCLM_022). 7. Pound the leaves of cabbage (JCLP_098) and mix them with the flour of cereal (JCLP_194) and plaster over. 8. Grind the root of cyclamen (JCLP_104) and put it where the pain is. 9. Grind the root of the plant that is called bryony (JCLP_037) and put it on top of the pain.

209.[158] If the joints of the feet or hands get stiff and like chalk stone. Do this treatment. Put gum ammoniacum (JCLP_015) into vinegar (JCLP_142) and let it do two days and nights and let it soak, then macerate with liquid pitch (JCLP_244), and put it on top of the pain, where the stiffening is. 2. Rinse the feet with sea (JCX_0496) water (JCLM_022) frequently. 3. Crush salt (JCLM_002) and the so-called nitron (JCLM_023) and mix it with table oil (JCLP_232) and apply frequently the pain of the joints.

210.[159] Make it for someone to stop bleeding. Grind maidenhair (JCLP_004) and let him drink its juice with wine (JCLP_144). 2. Pound fresh mint (JCLP_063) and mix the juice with vinegar (JCLP_142) and wine (JCLP_099) and let him drink. 3. Let him drink the tear of almonds (JCLP_017) with wine (JCLP_144). 4. Pound the tender leaves of bramble (JCLP_032) and put them where the blood flows. 5. Pound fresh rue (JCLP_157) and macerate with table oil (JCLP_232) and put it where the blood flows. 6. Crush mint (JCLP_063) and put it into his nose. 7. Fresh leaves of JCLP_167 ground and applied to the nose, stops the flow of the blood. 8. Put sulphur (JCLM_012) without fire (JCLM_005) onto the nose.

211.[160] If the stomach has an imbalance that is weakness and burns a lot from the inside, pound the leaves of polygonum (JCLP_164) and put them on the stomach. 2. Pontic sumach (JCLP_175) drunk with water (JCLM_036).

212.[160] If the belly runs, this stops it. Make JCLP_135 also fine, let him drink it with wine (JCLP_144). 2. Pound the leaves of vine (JCLP_016) and the tendrils and let him drink the juice. 3. Grind the root of gentian (JCLP_039) and let him drink it with wine (JCLP_144).

213.[160] If the stomach has heat inside. Pound the leaves of purslane (JCLP_041) and mix them with bran (JCLP_160) and put them on the stomach. 2. Boil wormwood (JCLP_028) with sesame oil (JCLP_190) and let him drink with wine (JCLP_144). 3. Eat unwashed lettuce (JCLP_119). 4. Grind the leaves of laurel (JCLP_049) and let him drink the juice. 5. Pound liquorice (JCLP_043) and let him drink the juice warm. 6. Grind the seed of parsley (JCLP_120) and let him drink them with wine (JCLP_099). 7. Let him eat domesticated or wild sowthistle (JCLP_062). 8. Boil this with water (JCLM_036) and let him drink. 9. Boil chamomile (JCLP_227) with water (JCLM_022) and let him drink. 10. Grind the root of aristolochia (JCLP_022) and put it on the stomach. 11. Boil asparagus (JCLP_026) and let him drink the juice.

214. Therapy for those who are suffering from the spleen and are swollen. Grind the seed of agnus castus (JCLP_115) and let him drink them with wine (JCLP_099). 2. Grind the root and leaves of scolopendrium officinale (JCLP_196) and put them on the spleen. 3. Boil the root and also the leaves of scolopendrium officinale (JCLP_196) with water (JCLM_022) and let him drink. 4. Pound asparagus (JCLP_026) and let him drink the juice. 5. Boil JCLP_002 with water (JCLM_022) and let him drink the decoction. 6. Soak one ounce of gum ammoniacum (JCLP_015) in wine (JCLP_099) for three days and nights and let him drink this wine (JCLP_099). 7. Boil wormwood (JCLP_028) and figs (JCLP_207) and water (JCLM_022) and boil their juice and macerate darnel (JCLP_006) flour and make a cataplasm and put it onto the spleen. 8. Grind the bark of the root of capers (JCLP_076) with wine (JCLP_099) and let him drink. 9. Pound tender leaves of ivy (JCLP_087) and put them on the spleen. 10. Boil the tender leaves of ivy (JCLP_087) with wine (JCLP_144) and let him drink. 11. Pound wild cress (JCLP_077) and put it on the spleen for three hours. Boil the leaves of willow (JCLP_072) and the fruit and the bark of the root with water (JCLM_022) and let him drink.

215.[161] If a person has colic. Do this treatment. Boil JCLP_002 with water (JCLM_022) and let him drink. 2. Let him drink the tip of dry dill (JCLP_073) and its seed with water (JCLM_022). 3. Boil rue (JCLP_157) with water (JCLM_022) and let him drink. 4. Pound marjoram (JCLP_184) and boil the juice with wine (JCLP_099) and let him drink. 5. Boil cumin (JCLP_105) with olive oil (JCLP_053) and administer this olive oil with a clyster. 6. Grind broad cumin (JCLP_162) and let him drink with water (JCLM_036). 7. Crush castor (JCA_0145) and let him drink it with wine (JCLP_099). 8. Grind the seed of JCLP_137 and let him drink it with wine (JCLP_099). 9. Rue (JCLP_157) and cumin (JCLP_105) and aniseed (JCLP_020); let him drink it with water (JCLM_022) and honey (JCA_0024).

216.[162] If someone is bitten by a scorpion. Crush the fruit of asphodelus (JCLP_027) and let him drink with wine (JCLP_144). 2. Pound marjoram (JCLP_184) and mix it with vinegar (JCLP_142) and ground salt (JCLM_002) and macerate and put it on the wound. 3. Pound the root of henna (JCLP_149) and let him drink it with wine (JCLP_144). Pound melissa (JCLP_129) and let him drink it with wine (JCLP_144).

217.[163] If it happens in a wound that worms are developing. Do this treatment in order to expel them. Pound the leaves of elm tree (JCLP_172) and put them on top. 2. Pound fresh wormwood (JCLP_028) and put it on top onto the wound. 3. Anoint with cedar oil (JCLP_083) and they will come out. 4. Do the same also if there are worms in the ear of a person.

218.[164] If someone is bitten by a sea JCA_2750. Do this treatment. Boil sage (JCLP_054) with wine (JCLP_144) and let him drink. 2. Pound the leaves of sage (JCLP_054) and put it onto the wound. 3. Grind the seed of asphodelus (JCLP_027) and let him drink it with with wine (JCLP_144). 5. Pound the leaves of asphodelus (JCLP_027) and put them on the wound.

219.[165] For those that are suffering from the spleen and from swelling. Boil marjoram (JCLP_184) with water (JCLM_022), then also add wine (JCLP_099) and let him drink. 2. Let him drink dry dung (JCA_0254) of cattle (JCA_1607) with wine (JCLP_144). 3. Boil the root of henna (JCLP_149) with water (JCLM_022). Then add wine (JCLP_099) and let him drink. 4. Grind the bark of capers (JCLP_076) and let him drink it with wine (JCLP_144). 5. Grind the root of hazelwort (JCLP_025) and let him drink it with wine (JCLP_144). 6. Grind the root of yellow flag (JCLP_009) and let him drink it with wine (JCLP_144). 7. Grind the root of a fig tree (JCLP_207) and mix it with barley (JCLP_100) flour and put it on top of the spleen just first boil the barley flour (JCLP_100) with water (JCLM_022). 8. Take the stone (JCLM_019) which is found inside the head of a frog and bind it onto the head of the swollen person. And it will be of great use.

220.[165] Therapy for inflammation. Pound pennyroyal (JCLP_040) with bran (JCLP_160) and put it onto the spleen. Sift bitter lupine (JCLP_118), and macerate their flour with bran (JCLP_160) and water (JCLM_022) and put it on top. 3. Pound fresh leaves of laurel (JCLP_049) and mix them with crumbs of bread (JCLP_024) and put it on top. 4. Pound the leaves of cabbage (JCLP_098) and mix them with bran (JCLP_160) and macerate and put them on top. 5. Pound the leaves of fresh of a gourd (JCLP_092) and put them on top. 6. Pound the leaves of henna (JCLP_149) and put them on top. 7. Pound the tender leaves of a plane tree (JCLP_161) and boil them with wine (JCLP_144) and put them on top. 8. Pound the shavings of dried of a gourd (JCLP_092) and put them on top. 9. Boil the leaves of beetroot (JCLP_189) and pound them and put them on top of the inflammation. 10. Grind linseed (JCLP_116) and fenugreek (JCLP_212) seed and mix, add also water (JCLM_022) and let it boil and then add lard (JCA_0077) of a goose (JCA_0246), of a pig (JCA_0266), of a bird (JCA_2318), of a fox (JCA_0624), of a bear (JCA_0990), marrow (JCA_0307) of a deer (JCA_0649), lard (JCA_0077) of a bull (JCA_0466), lard (JCA_0077) of a male calf (JCA_0515). Dissolve it all and macerate and put it on top of the inflammation. Dissolve also lard (JCA_0077) of a lion (JCA_0839).

221.[166] For loss of voice, cleansing. Let him eat raw and cooked garlic (JCLP_197). 2. Make all heal (JCLP_224) into an amount equal to the kernels of a chickpea (JCLP_055) and give him in the evening five and again five and let him swallow them and on top let him drink warm wine (JCLP_099) and he shall not eat anything.

222. It gets rid of lice. Mix the tear of ivy (JCLP_087) with vinegar (JCLP_142) and apply to the head. 2. Boil rhododendron (JCLP_180) with water (JCLM_022) and wash the head with it while it is hot. 3. Pound stavesacre (JCLP_003) and mix it with table oil (JCLP_232) and apply it to the head.

223.[167] For the so-called *khoiradas*. Pound plantain (JCLP_023) and put it on top. 2. Pound maidenhair (JCLP_004) and put it on top. 3. Mix the flour of lupine (JCLP_118) with vinegar (JCLP_142) and put them on top onto the wound. 4. Pound monk’s rhubarb (JCLP_111) and mix it with vinegar (JCLP_142) and put it on top. 5. Grind linseed (JCLP_116) and put it on top. 6. Grind fleawort (JCLP_235) and put it on top with vinegar (JCLP_142). 7. Pound the so-called JCLP_038 and mix the juice with bran (JCLP_160) and put it on top. 8. Pound the root of cyclamen (JCLP_104) and put it on top. 9. Grind the root of JCLP_051 and put it on top. 10. Pound the root of JCLP_051 and let him drink the juice. 11. Let him drink also the juice of the root of it.

224. Therapy for psora. Boil bitter lupine (JCLP_118) with water (JCLM_036) and water (JCLM_022) and rinse the psora warm with it. 2. Boil cedar oil (JCLP_083) with vinegar (JCLP_142) and apply in the bath. 3. Fry the heart of rhododendron (JCLP_180) and of myrtle (JCLP_134) with table oil (JCLP_232) and apply. 4. Pound the leaves of ivy (JCLP_087) and boil them with water (JCLM_022) and let him drink. 5. Boil rue (JCLP_157) with water (JCLM_036) and rinse in the bath. 6. Pound stavesacre (JCLP_003) also with olive oil (JCLP_053) and apply.

225. It heals ear ache. Drip warm almond oil (JCLP_017) into the ear. 2. Drip oil (JCLP_053) of nuts (JCLP_079) into the ear. 3. Drip warm laurel oil (JCLP_049) into the ear. 4. Drip JCLP_030 into the ear. 5. Grind the seed of JCLP_051 and remove the juice and mix it with table oil (JCLP_232) and apply. 6. Pound mint (JCLP_063) and mix the juice with honey (JCA_0024) and drip it into the ear. 7. Pound the shavings of a fresh gourd (JCLP_092) and collect the juice and unify with rose oil (JCLP_181) and drip it into the ear. 8. Pound onion (JCLP_102) and drip the juice into the ear. 9. Boil fresh rue (JCLP_157) with olive oil (JCLP_053) and warm it up and drip it into the ear. 10. Drip radish oil (JCLP_176) into the ear. 11. Grind cumin (JCLP_105) and also add olive oil (JCLP_053) and boil. And warm it up and drip it into the ear. 12. Pound the root of asphodelus (JCLP_027) and mix the juice with crushed of myrrh (JCLP_133) and rosemary (JCLP_050) and drip into the ear.

226.[168] If you want that someone is not thirsty. Do this treatment. Keep an olive (JCLP_053) JCO_2045 on your tongue. 2. Slurp an egg (JCA_0630) of a bird (JCA_0346) in the morning raw on empty stomach. 3. Boil the seed of lettuce (JCLP_119) and liquorice (JCLP_043) with water (JCLM_022) and let it cool down and give it to him and let him drink. 4. Boil dates (JCLP_222) and carob (JCLP_139) and liquorice (JCLP_043) and water (JCLM_022) and cool it down and let him drink.

227. Alphoi is called if the person develops something like a black lentil (JCLP_219) on the face. Do this treatment. Crush white JCU_0278 and mix it with vinegar (JCLP_142) and apply. 2. Crush black JCU_0278 and mix it with olive oil (JCLP_053) and vinegar (JCLP_142) and apply either in the sun or near a fire. 3. Apply crushed sulphur (JCLM_033) and vinegar (JCLP_142). 4. Mix crushed litharge (JCLM_018) and sulphur (JCLM_033) and crushed stavesacre (JCLP_003) with vinegar (JCLP_142) and apply.

228. If they develop something like a white lentil (JCLP_219) on the face, do this treatment. Crush dry myrrh (JCLP_133) and sulphur (JCLM_033) and mix it with vinegar (JCLP_142) and apply. 2. Mix the heart of ink-gall (JCLP_085) and liquid pitch (JCLP_244) and apply.

229. Alopecia is if the hair of the head falls out. Do this treatment. Burn the leaves of reed (JCLP_075) and mix them with liquid pitch (JCLP_244) and lard (JCA_0077) of a sheep (JCA_0709) and shave the entire head and apply. 2. Mix crushed stavesacre (JCLP_003) and mastic (JCLP_125) and mercury (JCLM_037) and vinegar (JCLP_142) together and apply to the head. 3. Dissolve lard (JCA_0077) of a fox (JCA_0624) and of a goat (JCA_0478) add resin (JCLP_158) and vinegar (JCLP_142) and apply.

230. Therapy for inflammation of the parotis. Mix lard (JCA_0077) of a male calf (JCA_0515) of cattle (JCA_0769) and lime (JCLM_007) and apply. 2. Grind fenugreek (JCLP_212) seed and cress seed (JCLP_077) and add also lime (JCLM_007) and apply.

231. If someone urinates blood. Do this treatment. Boil carob (JCLP_139) and also crush yellow flag (JCLP_009) and mix it with the juice of carob (JCLP_139) and let him drink.

232. For cough. Boil hyssop (JCLP_216) and pennyroyal (JCLP_040) and dried figs (JCLP_071) with water (JCLM_022) and let him drink. 2. Mix the crushed seed of rue (JCLP_157) and cumin (JCLP_105) and pepper (JCLP_154) with honey (JCA_0024) and let him drink. 3. Grind storax (JCLP_206) the same amount as a vetch (JCLP_146) and let him drink it with water (JCLM_022) for three days. 4. Let him drink crushed JCLP_140 and cumin (JCLP_105) and honey (JCA_0024). 5. Let him drink the seed of mustard (JCLP_192), ground a lot, with warm water (JCLM_022). 6. Grind the seed of cress (JCLP_077) smoothly and let him drink it with water (JCLM_022). 7. Pound the leaves of rue (JCLP_157) and mix the juice with butter (JCA_0330) and let him drink it and apply to the chest. 8. Pound JCLP_135 and mix its juice with honey (JCA_0024) and let him drink. 9. Boil pennyroyal (JCLP_040) with water (JCLM_022) and let him drink it with honey (JCA_0024). 10. Let him chew sesame (JCLP_190) frequently. 11. Crush pine resin (JCLP_177) and let him drink it with wine (JCLP_144). 12. Boil hyssop (JCLP_216) and pennyroyal (JCLP_040) and fenugreek (JCLP_212) with honey (JCA_0024) and wine (JCLP_099) and let him drink. 13. Boil dill (JCLP_073) and cumin (JCLP_105) and rue (JCLP_157) with wine (JCLP_099) and let him drink. 14. Boil pennyroyal (JCLP_040) and rue (JCLP_157) and butter (JCA_0330) with honey (JCA_0024) and water (JCLM_022) and let him drink in the bath. 15. Boil the leaves of celery (JCLP_186) with water (JCLM_022) and let him drink. 16. Apply butter (JCA_0330) to the chest. 17. Boil the leaves of rue (JCLP_157) and cumin (JCLP_105) and a little wine (JCLP_099) and apply to the chest. 18. Make crushed henna (JCLP_149) very fine and let him drink it with good wine (JCLP_144) in the bath.

233.[169,170] If his mouth smells. Crush 15 kernels of pepper (JCLP_154) and burn carob (JCLP_139) and crush one *hexagion* of the peel of pomegranate (JCLP_182) and one ink-gall (JCLP_085) without a hole and mix it with honey (JCA_0172) and apply to the area that is rotten. 2. Apply good (JCX_1217). 3. Mix fine yellow orpiment (JCLM_006) with honey (JCA_0172) and apply hemp (JCLP_205) and put hemp (JCLP_205) with your finger into the mouth where the rot is. 4. Burn the bark of willow (JCLP_072) and sheet of paper (JCLP_230) and their ashes (JCX_0126), add also fine pepper (JCLP_154) and make it smooth with honey (JCA_0172) and put it on the rot. 5. Unify mercury (JCLM_035) and fine pepper (JCLP_154) and honey (JCA_0024) and apply to the area. 6. Mix the flour of lupine (JCLP_118) and the bile of a bear (JCA_0435) and apply it to the area.

234.[171] For the bubonic plague that is *aporufas*. Macerate live lime (JCLM_007) and honey (JCA_0024) and JCX_2546 and put it on top.

235.[172] For cramps. Boil the fine seed of a radish (JCLP_176) and ink-gall (JCLP_085) with wine (JCLP_099) and let him drink. 2. Fumigate the peel of pomegranate (JCLP_182) and dry resin (JCLP_158) from below. 3. Boil oregano (JCLP_141) with wine (JCLP_099) and let him drink.

236. If someone swallows leeches. Let him drink boiled vinegar (JCLP_142). 2. Put JCU_0677 on top of coals (JCLM_015) and fumigate into the mouth. And let him swallow the smoke. 3. Leeches and snakes are expelled by drinking unripe olive oil (JCLP_053). 4. Mix butter (JCA_0330) and vinegar (JCLP_142). Then heat up iron (JCLM_027) well and put it inside and let it extinguish. Then let him drink the butter (JCA_0330) as well as the vinegar (JCLP_142).

237. To bring down the milk (JCX_0085) of a woman (JCX_0362). Grind nigella (JCLP_126) and let her drink it with wine (JCLP_144) in the bath. 2. Grind the seed of fenugreek (JCLP_212) and put it on top of the breasts. 3. Let her eat onion (JCLP_102). 4. Let her eat lupine (JCLP_118) that are called lupines (JCLP_064). 5. Boil the root of fennel (JCLP_123) with wine (JCLP_099) and let her drink. 6. Boil the branches of stinging nettle (JCLP_211) with water (JCLM_022) and let her drink. 7. Pound mint (JCLP_063) and apply it warm to the breasts.

238.[173] If the knees appear to be cold like ice. Mix fine lime (JCLM_007) and liquid pitch (JCLP_244) and macerate well and spread it on the cloth (JCLP_151) and put it on top. 2. Grind the kernels of laurel (JCLP_049) and sift and macerate with sea (JCX_1100) water (JCLM_022) and put them on a cloth (JCLP_151) and put it where the pain in the knees is just first sift the kernels of the laurel (JCLP_049).

239. For problems urinating. Grind the root of lettuce (JCLP_119) and let him drink it in the morning with water (JCLM_022). 2. Grind the seed of cress (JCLP_077) and let him drink it with wine (JCLP_144). 3. Grind JCU_0677 and put it into the hole of the penis and he will immediately want to urinate. 4. Boil the root of celery (JCLP_186) and of a radish (JCLP_176) and leek (JCLP_169) with wine (JCLP_144) and let him drink it. 5. Boil the root of fennel (JCLP_123) and of celery (JCLP_186) with good old wine (JCLP_099) and let him drink it. 6. Let him eat crabs (JCA_1918) and let him also drink their juice. 7. Hazelwort (JCLP_025) pennyroyal (JCLP_040) and pepper (JCLP_154); let him drink with wine (JCLP_144) in the bath but let them boil first. 8. Crush the seed of celery (JCLP_186) and seven cloves of garlic (JCLP_197) together, boil with wine (JCLP_099) and let him drink. 9. Pound JCLP_035 and let him drink its juice. 10. Boil chamomile (JCLP_227) with water (JCLM_022) and let him drink. 11. Let him drink laurel oil (JCLP_049) with wine (JCLP_144) in the bath. 12. Boil the root of asparagus (JCLP_026) and willow (JCLP_072) with white wine (JCLP_099) and let him drink. 13. Boil plantain (JCLP_152) and maidenhair (JCLP_004) with water (JCLM_022) and let him drink. 14. Chop the feet and wings and head of a JCA_2158 and weigh them so that they come equal to four kernels and let him drink.

240. On dysentery. The seed of fenugreek (JCLP_212) roasted in the sun and lentils (JCLP_219) and ink-gall (JCLP_085) and cumin (JCLP_105). Grind it all and let him drink with warm wine (JCLP_144) in the morning on empty stomach. 2. Let him drink the juice of JCLP_035 with wine (JCLP_144) and water (JCLM_022) warm in the morning.

241. It brings down the menstruation of women and the urine (JCA_2332) and expels children from the womb and is useful for the spleen. Boil the plant called germander (JCLP_226) with wine (JCLP_144) and let her drink. 2. Fumigate all heal (JCLP_224) from below. 3. Crush all heal (JCLP_224) and put it outside on the hidden area of the woman. 2. Crush indigo (JCLP_117) and nigella (JCLP_126) and macerate with honey (JCA_0172) and make a cloth (JCLP_151) like a knot, put it inside and sew it together and give it to another woman and let her put it into her womb with her hand. Bind a thread to the knot and if you want to remove it pull the thread and you will take it out. This is called pessary by the doctors. 3. Make fresh all heal (JCLP_224) in a pessary.

242.[174] For external and internal hemorrhoids. Boil the skin of a snake (JCA_2345) in table oil (JCLP_232) and put it on top of coals (JCX_0499) that are set on fire and fumigate, so that the smoke rises up to the affected area. 2. Boil the bark of the root of JCLP_150 with wine (JCLP_099) and let him drink inside the bath. And let him be fasting for four hours. 3. Sprinkle the flour of lupine (JCLP_118) on top once you have sieved it but first wipe them with wine (JCLP_099). 4. Grind the seed of the kernels of laurel (JCLP_049) and collect the juice and the heart of the kernels and make these fine and sprinkle it on top. 5. Burn the head of a tuna (JCA_1890) or a salted bonito (JCA_2359), the one that you have and sprinkle its ashes (JCX_0126) on top. 6. Crush dry equisetum (JCLP_069) that is called equisetum (JCLP_163) and let him drink with wine (JCLP_144). 7. Fry the root of equisetum (JCLP_163) in a pan with table oil (JCLP_232) and apply. 8. Crush lead white (JCLM_042) and mix it with milk (JCX_0085) of a woman (JCX_0118) and apply. 9. Grind the root of lily (JCLP_101) and mix it with barley flour (JCLP_100) and also add water (JCLM_022) and boil it and spread it on a cloth (JCLP_151) and put it on top. 10. Mix donkey (JCA_0619) milk (JCX_0085) and crushed JCX_0404 and apply. 11. Pound JCX_0114 and JCLM_031 and unify and apply. 12. Boil the seed of a mulberry tree (JCLP_208) and plantain (JCLP_152) with water (JCLM_036) and let him drink. First wipe the area with vinegar (JCLP_142), then take burnt leaves of reed (JCLP_075) and sprinkle them on top. Crushed dry soft dung (JCA_0254) of a dog (JCA_0920), rinse the area first with wine (JCLP_144) then sprinkle it on top. Burn yolk (JCX_0114) of an egg (JCA_0105) and sprinkle it on top.

243.[175] For a person of whom the anus prolapses. Take crushed JCX_0404 and lead white (JCLM_042) and add wine (JCLP_099) and macerate and then apply. 2. Melilot (JCLP_128) and dry roses (JCLP_181) and crushed lead white (JCLM_042). Add also wine (JCLP_099) and boil it and then apply to the area. 3. Boil a mushroom (JCO_1450) with water (JCLM_036) and pound it and mix it with butter (JCA_0330) and macerate and apply to the area with your finger. 4. First apply wine (JCLP_099) to the area then take crushed fine sieved millet (JCLP_082) bran (JCLP_160) and sprinkle it on top like this and then put his colon back inside that has been anointed with bran (JCLP_160) of the millet (JCLP_082).

244. If someone has flat worms. Do this treatment. Grind cress seed (JCLP_077) and crushed pepper (JCLP_154) and let him drink with water (JCLM_022) in the morning on empty stomach. 2. Grind nigella (JCLP_126) and let him drink it with unmixed wine (JCLP_099) or with fish (JCA_0526) sauce (JCA_0489). 3. Grind all heal (JCLP_224) and let him drink it with wine (JCLP_099). 4. Grind all heal (JCLP_224) and put it onto the navel of the person. 5. Grind the seed or the leaves of JCLP_096 and let him drink them with water (JCLM_022). 6. Let him eat garlic (JCLP_197) with old table oil (JCLP_232). 7. Pound fresh mint (JCLP_063) and let him drink the juice in the morning. 8. Mix crushed cumin (JCLP_105) with the bile of a bull (JCA_0466) and put it onto the belly button. 9. Macerate the flour of lupine (JCLP_118) with honey (JCA_0024) and put it on top. 10. Grind coriander (JCLP_091) finely and unify with table oil (JCLP_232) and put it onto the belly button. 11. Grind hemp seed (JCLP_238) and let him drink it with water (JCLM_022). 12. Boil tender stems of cabbage (JCLP_098) that is the tips with clean water (JCLM_022) then take one *hexagion* of the water (JCLM_022) and coarse salt (JCLM_002) one *hexagion* and he will expel the seed of the worms. 13. Grind the flour of lupine (JCLP_118) and wormwood (JCLP_028) and add laurel oil (JCLP_049) and macerate all together put it onto the navel.

245.[176] For *ekbata* and *aporyfas*. Beetroot (JCLP_189) is useful and mix the juice with cereal (JCLP_194) flour and macerate them together and put it on top. 2. Macerate oak mistletoe (JCLP_067), crushed glass (JCLM_034) and JCX_2547 and table oil (JCLP_232) and put it on top. 3. Grind the green leaves of reed (JCLP_075) and mix the juice with very finely grated lead (JCLM_024) and macerate and put it on top. 4. Mix black crushed JCU_0278 with propolis (JCA_0710) and macerate and put it on top. 5. Crush dung (JCA_0254) of a wild pigeon (JCA_1393) and unify and macerate with honey (JCA_0024) and put it on top. 6. Crush dung (JCA_0254) of a goose (JCA_0543) and unify it and macerate with honey (JCA_0024) and put it on top. 7. Pound the root of monk’s rhubarb (JCLP_111) and macerate with lard (JCA_0077) of a pig (JCA_0266) and put it on top.

246. If someone urinates at night involuntarily. Do this treatment. That is boil JCLP_167 with wine (JCLP_099) and let him drink. 2. Crushed myrrh (JCLP_133) and the juice of JCLP_167 with wine (JCLP_099), let him drink it. 3. Let him drink the burnt hooves of a black pig (JCA_0952) with wine (JCLP_144). 4. Burn the bladder of a goat (JCA_0965) and let him drink it with vinegar (JCLP_142) and wine (JCLP_099). 5. Let him eat the tongue of a goose (JCA_0543). 6. Grind the kernels of laurel (JCLP_049) and the seed of rue (JCLP_157) and mix it and unify with rose oil (JCLP_181) and apply it to the crotch of the person. 7. Let him drink the testicles of a hare (JCA_0453) with good wine (JCLP_099). 8. Let him eat for three days the seed of wild rue (JCLP_157) that have been roasted in the sun. 9. Burn the throat of a crow (JCA_2385) and let him drink the ashes (JCX_0126) with wine (JCLP_144).

247.[177] If someone is bitten by a viper. Pound rue (JCLP_157) and let him drink the juice. 2. Crush what is called by the doctors black cardamom (JCLP_018) and let him drink it with the juice of rue (JCLP_157). 3. Mix crushed mustard (JCLP_192) and cress seed (JCLP_077) with vinegar (JCLP_142) and apply.

248.[179] For migraine. The entrails (JCX_0794) of the earth (JCX_0773) that is the large worms with which people catch the fishes (JCA_0594): roast these on the stove and mix in fifteen crushed kernels of pepper (JCLP_154) with vinegar (JCLP_142) and apply to the head. 2. Add ground dung of a wild pigeon (JCA_1392) to vinegar (JCLP_142) and apply. 3. Boil the seed of agnus castus (JCLP_115) with table oil (JCLP_232) and grind and apply to the forehead and the temples. 4. Macerate live lime (JCLM_007) with honey (JCA_0024) and apply. 5. Put the heart of rue (JCLP_157) into the ear and bind the head tightly and cover it well. 6. Grind bitter almonds (JCLP_017) and crushed mastic (JCLP_125) and mix it with vinegar (JCLP_142) and apply to the forehead. 7. Pound the tender shoots of ivy (JCLP_087) and mix the juice with rose oil (JCLP_181) and vinegar (JCLP_142) and apply. 8. Put dry leaves of rhododendron (JCLP_180) on burning coals (JCLM_015) and fumigate into the nose.

249.[180] If someone is suffering from sciatica. Boil chickpeas (JCLP_055) with water (JCLM_022) and let him eat and let him drink the broth. 2. Boil the bark of the so-called wheat (JCLP_059) with water (JCLM_022) and let him drink. 3. Grind mustard (JCLP_192) and pepper (JCLP_154) and mix it with honey (JCA_0024) and apply. 4. Bind the root of cyclamen (JCLP_104) or laurel (JCLP_049) to the place. 5. Crush aloe (JCLP_012) and pine resin (JCLP_177) and pepper (JCLP_154) and put it on top on the area but first anoint the area with honey (JCA_0024). 6. Boil the leaves of cabbage (JCLP_098) and mix the ashes (JCX_0126) with lard (JCA_0077) of a pig (JCA_0266) and put it on top. 7. Boil table oil (JCLP_232) and fatty resinous wood (JCLP_046) and apply to the area. 8. Anoint the area where the pain is with the so-called JCU_1574. 9. Let him eat boiled garlic (JCLP_197) with table oil (JCLP_232), and fish (JCA_0526) sauce (JCA_0489). 10. Pound pennyroyal (JCLP_040) and pepper (JCLP_154) and macerate with water (JCLM_022) and put it on top. 11. Mix butter (JCA_0330) with dill oil (JCLP_073) and apply to the area. 12. Burn tendril (JCLP_090) and mix the ashes (JCX_0126) with liquid pitch (JCLP_244) and put it on top. 13. Pound the so-called JCLP_038 and mix its juice with bran (JCLP_160) and put it on top. 14. Pound the root of cyclamen (JCLP_104) and put it on top. 15. Hang the root of cyclamen (JCLP_104) around his neck. Pound the root of JCLP_051 and put it on top. Pound the root of JCLP_051 and let him drink the juice.

250.[181] For those suffering from jaundice that is if their face gets yellow. Grind yellow orpiment (JCLM_006) and mix it with yolk (JCX_0114) of an egg (JCA_0105) and let him drink inside the bath. 2. Grind centaury (JCLP_084) and let him drink it with water (JCLM_022) in the bath. 3. Grind nigella (JCLP_126) and ginger (JCLP_060) and add vinegar (JCLP_142) and macerate and drip this vinegar (JCLP_142) into his nostrils. 4. Boil the root of cyclamen (JCLP_104) with wine (JCLP_099) and let him drink it warm. 5. Put the kernels of carob (JCLP_139) into wine (JCLP_099) and leave them for one day and one night, then let him drink the wine (JCLP_099). 6. Crush hare (JCA_0453) dung (JCA_0254) and let him drink with wine (JCLP_144). 7. Grind celery seed (JCLP_186) and let him drink with wine (JCLP_144) in the bath. 8. Let him drink boiled pennyroyal (JCLP_040) with wine (JCLP_099). 9. Boil fennel (JCLP_123) and dill (JCLP_073) and celery (JCLP_186) with water (JCLM_022) and let him drink. 10. Let him drink male calf (JCA_1215) and aquilaria agallocha (JCLP_138) and frankincense (JCLP_114) and castor (JCA_0145) and water (JCLM_036) of the holy Epiphany ceremony (JCO_1874) and gum ladanum (JCLP_110).

251.[181] For leprosy. Grind the root of asphodelus (JCLP_027) and remove the juice. Grind also cress seed (JCLP_077) and the flour of darnel (JCLP_006) and unify these and macerate and add vinegar (JCLP_142) and apply to the leprous lesions. 2. Grind the root of asphodelus (JCLP_027) and collect the juice. Also crush sulphur (JCLM_033) and unify and apply to the leprous lesions.

252.[182] For psora. Grind stavesacre (JCLP_003) and the seed of cress (JCLP_077) and add vinegar (JCLP_142) and macerate and apply it to the psora. 2. Grind the root of lily (JCLP_101) and mix it with honey (JCA_0024) and apply. 3. Grind sulphur (JCLM_033) and dung (JCA_0254) of a goat (JCA_1405) and add vinegar (JCLP_142) and apply it to the psora. 4. Boil the leaves of rhododendron (JCLP_180) with table oil (JCLP_232) then remove the leaves and add wax (JCA_0450) and crushed sulphur (JCLM_033) and apply to the psora in the sun. 5. Take rose oil (JCLP_181) and ground litharge (JCLM_018) and lead white (JCLM_042) and liquid pitch (JCLP_244) and vinegar (JCLP_142) and mix these and apply in the sun.

253.[183] On lichen. Apply liquid pitch (JCLP_244) to the lichen. 2. Soak the tear of damson (JCLP_047) one day and one night in vinegar (JCLP_142) and apply. 3. Pound the leaves of agnus castus (JCLP_115) and put them on top or mix with vinegar (JCLP_142). 4. Crush the leaves of capers (JCLP_076) and mix them with vinegar (JCLP_142) and apply. 5. Grind frankincense (JCLP_114) and mix it with vinegar (JCLP_142) and apply. 6. Crush sulphur (JCLM_033) and mix it with vinegar (JCLP_142) and apply. This book comes to an end.

## Data Availability

Figshare: Materia medica lists with tags for philological purposes. https://doi.org/10.17637/rh.24100080.v1 ^8^. This project contains the following underlying data: latest tags.xlsx. (The full list of tags). Data are available under the terms of the Creative Commons Attribution 4.0 International license (CC-BY 4.0).

## Footnotes

^1^ Due to constraints of space, we are unable to provide full background on ancient and medieval medical history. For further information along with bibliography, see for instance Nutton, V. (2023). Ancient Medicine (3rd ed.). Routledge. https://doi.org/10.4324/b22934. In some instances, a translation and analysis will of course be complicated or impossible, for instance if the text is incomplete or corrupt.

^2^ The ancient Greek system was based on four qualities, hot, cold, wet, and dry, and four humours, blood, hot and moist, yellow bile, hot and dry, phlegm, cold and wet, and black bile, cold and dry. Most illnesses were thought to have been caused by an imbalance in quality or humour, which was then treated with opposites. If a patient had fever, a hot imbalance, they were either given medication to cool them down or cooled down through a change in the environment, for instance by moving them to a colder space. We still use ancient terminology inadvertently, for instance by calling a person phlegmatic, or by using the phrase ‘catching a cold’.

^3^ The text was first edited in 1

^4^ In the following, JP stands for the original version of the Therapeutics, previously known as *א*, and JC stands for the commentary version of the Therapeutics, previously known as ω. This nomenclature originated among the scientists on our team.

^5^ 2

^6^ In this step, dictionaries were also consulted, for instance ^3– 6^

^7^ 7

^8^ link [last accessed 6.9.2023]

^9^ See link [last accessed 31.08.2023]

^10^ This system draws on Model 6.II in 9. For details on this system and why it was used see 2

^11^ See 10 and 1 10f.

^12^ We owe this translation, and also some of the translations of subsequent passages to 11 They are, however, not verbatim quotes and may be modified. The basis for the analysis was Wellmann’s edition.

^13^ For details on this rather complex matter see 10

^14^ See for instance Leo medicus III 28 (= 143 Ermerins): Λεύκωμά ἐστιν οὐλὴ ἐξ ἑλκώσεως τοῦ κερατοειδοῦς χιτώνος παχεῖα καὶ λευκή… ‘Whitening (leukoma) is a thick and white scar from an ulceration of the cornea…’ ^12^. Leo is a particularly good source in this respect, as his work is part of the same broader tradition as JC.

^15^ Dioscorides *De Materia Medica* III 84, 2 (= 101, 14 f. Wellmann): Σμήχει δὲ τὰ ἐν ὀφθαλμοῖς λευκώματα καὶ τραχύτητας βλεφάρων τήκει.

^16^ For a definition of the term cloud in the eye see for instance: Περὶ νεφελίου. Νεφέλιον δὲ λέγεται, ὅταν ἐξ ἐπιπολαίου ἑλκώσεως λεύκωμα γένηται λεπτὸν καὶ διεσπασμένον ἐοικὼς νεφέλῃ. Ἰώμεθα δὲ καὶ αὐτὸ ὥσπερ τὸ λεύκωμα. Leo medicus ΙΙΙ 26 (= 141 Ermerins). ‘On a small cloud ( *nefelion*). A small cloud is called when a thin and expansive whitening ( *leukoma*) arises from a superficial ulceration that is similar to a cloud. We treat it like a whitening ( *leukoma*).’

^17^ link [last accessed on the 20th of March 2023].

^18^ See for instance Paul of Aegina III 22, 31 (= I 185 Heiberg): ἀμβλυωπία δὲ ἀμυδρότης τοῦ ὁρᾶν ὑπό τινος ἀδήλου αἰτίας γινομένη. ‘Amblyopia is dimness of the sight that arises from some unknown cause.’ ^13^ Aetius Amidenus VII 49, 2 (= II 303 Olivieri), citing Galen, states that *amblyopia* can be down to multiple aetiologies ^14^.

^19^ The passage can be found in JP 100 2-4 and JC 120.

^20^ See Paul of Aegina III 22, 31 (= I 185, 18 f. Heiberg): …ἀπό τε κανθῶν κένωσις αἵματος καὶ βδελλῶν προσβολὴ κατὰ τοὺς κροτάφους τρίψεις τε τῶν κάτω μερῶν. Προϊόντος δὲ τοῦ χρόνου καὶ τοῖς πταρμικοῖς χρηστέον καὶ νήστεσιν ἐμέτοις… ‘…emptying of the blood from the inner corners of the eyes and application of leeches on the temples and massage of the lower limbs. With time one should also use agents that induce sneezing and vomiting on empty stomach…’.

^21^ See for instance 15 and 16

^22^ 17 409.

^23^ link [last accessed 31.08.2023]

^24^ 18 1110–1122. See also 19. 94.
