# Supplementary material for: Interpreting materia medica. A case study on Ioannes Archiatrus
Source: Wellcome Open Res. 2024 Jun 19;8:502. Originally published 2023 Nov 2. [Version 2] doi: 10.12688/wellcomeopenres.20061.2 (PMC11109676; doi:10.12688/wellcomeopenres.20061.2)
Supplement: Peer review history

# Reviewer report: Reviewer response for version 2

**DOI:** 10.21956/wellcomeopenres.24864.r87200

**Published:** 2024-06-25

**Authors:** Alpo Honkapohja

**Affiliations:** 1 School of Humanities, Tallinn University, Tallinn, Estonia

I am satisfied with the revisions.

Is the rationale for developing the new method (or application) clearly explained?

Yes

Is the description of the method technically sound?

Partly

Are the conclusions about the method and its performance adequately supported by the findings presented in the article?

Yes

If any results are presented, are all the source data underlying the results available to ensure full reproducibility?

Yes

Are sufficient details provided to allow replication of the method development and its use by others?

Partly

Reviewer Expertise:

History of medicine, corpus linguistics

I confirm that I have read this submission and believe that I have an appropriate level of expertise to confirm that it is of an acceptable scientific standard.

# Reviewer report: Reviewer response for version 1

**DOI:** 10.21956/wellcomeopenres.22219.r81138

**Published:** 2024-06-08

**Authors:** Alpo Honkapohja

**Affiliations:** 1 School of Humanities, Tallinn University, Tallinn, Estonia

The paper presents an interesting and also impressive approach to tagging and translating pharmaceutical terms. The target audience are scientists engaged in recreating and empirically evaluating remedies described in ancient and medieval (in this case Byzantine Greek) texts.

 The method involves tagging texts and subsequently concordancing them using a freeware corpus tool, AntConc, developed by Laurence Anthony. The methodology seems sound, and AntConc is widely used and reliable. However, I missed some technical details. While tags used by the project are described in transparent way, I could not find information on file format used, as AntConc can handle ASCII (.txt), .rtf or .xml files. In the interests of transparency and reproducibility, this information should be added.

 What is more, I think some comments in the introduction could benefit from hedging. To be more specific, this claim struck me as simply not true:

*"Some types of content in ancient or medieval medical writings can easily be translated, for instance physiological works or descriptions of surgery. While some of the underlying ideas differ widely from our modern understanding, for instance the concept of black bile, or melancholia in Greek, these matters can easily be explained in an introduction or appendix."*

 There are challenges related to translating all kinds of medical texts, including "physiological works" and "descriptions of surgery". Some physiological/anatomical terms can have multiple meanings. For example, the Germanic word *womb*(from Proto-Germanic *wamb*) can in English medical texts refer to 'the human stomach' and 'the visceral cavity of the human body containing most of the digestive organs', 'the surface of the human body over the stomach or abdomen', 'the abdomen of a statue or an image', 'a belly-shaped part of an object', or 'the human uterus, womb' (definitions from the Middle English dictionary). Deciphering the exact meaning in a physiological text is not always something that can be done "easily", especially considering that many texts had complicated circulation histories. They could be translations from Latin, from Latin via Anglo-Norman French, from Greek via Latin, from Arabic via Latin, from Greek via Arabic and Latin, etc. Since medical terminology was far from standardised, the Germanic word *womb* could compete with other words (including Latin, Greek and French borrowings) and other translation strategies used by translators of medical works. For example, the Historical Thesaurus of English lists many different words used with the meaning 'female sex organs::womb' - *womb*, *mother*, *cloister*, *matrice*, *matrix*, *bermother*, *uterus*, *venter*, *oven*, etc. (not a comprehensive list)

 Descriptions of surgical procedures in medieval surgical texts are subject to similar problems. They usually lack clear definitions of terminology. The texts may have complicated transmission histories, involving possible translation across multiple language and copying, leading to similar difficulties in deciphering exact meanings. Some surgical texts may contain descriptions and/or illustrations of surgical instruments, which may get distorted beyond recognition over repeated copying from MS to MS (similarly to illustrations of herbs in herbals).

 The authors illustrate their claim with reference to "melancholy" in the humoral theory, which is fair enough since the four humours can arguably be directly translated as *melancholy*, *choler*, *phlegm* and *sanguine*. However, there are complications. The humoral theory was never a completely unified framework - Aristotle's and Galen's versions of the theory differed considerably (I assume Hellenistic medical works would be more on the Aristotelian side of things). Phlegm is perhaps the most problematic of the four humours, as it seems the Ancients may have grouped 2-3 different bodily fluids under it. It is also not always clear whether references are to bodily fluids or symptoms. For example, melancholy begins to be used for psychological conditions associated with depression in works such Robert Burton's *Anatomy of Melancholy* (1621) and there is semantic expansion already prior to it.

 Admittedly, my experience is more in Latin and Middle English vernacular medical texts than Greek philology, but since the authors did explicitly mention "medieval medical writings", I think it needs to be pointed out. Despite this lengthy comment, I believe this issue can be easily addressed by hedging and toning down the claim in the introduction. There are difficulties related to terminology in all kinds of texts - they are not exclusive to botany and pharmacology!

 These minor complaints aside, on the whole, I think this is a very interesting approach with much potential and definitely worth indexing!

Is the rationale for developing the new method (or application) clearly explained?

Yes

Is the description of the method technically sound?

Partly

Are the conclusions about the method and its performance adequately supported by the findings presented in the article?

Yes

If any results are presented, are all the source data underlying the results available to ensure full reproducibility?

Yes

Are sufficient details provided to allow replication of the method development and its use by others?

Partly

Reviewer Expertise:

History of medicine, corpus linguistics

I confirm that I have read this submission and believe that I have an appropriate level of expertise to confirm that it is of an acceptable scientific standard, however I have significant reservations, as outlined above.

# Reviewer report: Reviewer response for version 1

**DOI:** 10.21956/wellcomeopenres.22219.r81139

**Published:** 2024-05-21

**Authors:** Suzana Inić

**Affiliations:** 1 University of Zagreb, Zagreb, Croatia

The authors have developed a very valuable method and tools that enable scientists from various fields to read and research premodern pharmaceutical primary sources. These tools also provide confidence factors for individual pharmaceutical terms that contribute to the balance of primary source translation and caution in translation. The authors conclude that their method can be modified and applied to other primary sources.

 Can a brief explanation be given if the method and applied tools require more additional efforts for its modification to other primary sources and what should be done.

 It is stated in the philological notes: Our work does not aim to provide a dictionary that can universally be applied to all ancient and medieval medical texts. While we acknowledge that Dioscorides' work was seminal in this field, we are well aware of the fact that a given plant name may have referred to an entirely different one species in another geographic location or at another point in time.

 I have a suggestion to create a dictionary with medical, botanical and pharmaceutical terms for universal application in the future. Most medieval medical texts were compiled from ancient medical texts based mainly on the works of Pliny and Dioscorides. A dictionary of pharmaceutical terms could be made based on those works with the appropriate confidence factors for plant names and other medical and pharmaceutical terms, as was done for this text.

Is the rationale for developing the new method (or application) clearly explained?

Yes

Is the description of the method technically sound?

Yes

Are the conclusions about the method and its performance adequately supported by the findings presented in the article?

Yes

If any results are presented, are all the source data underlying the results available to ensure full reproducibility?

Yes

Are sufficient details provided to allow replication of the method development and its use by others?

Yes

Reviewer Expertise:

history of pharmacy

I confirm that I have read this submission and believe that I have an appropriate level of expertise to confirm that it is of an acceptable scientific standard.

# Reviewer report: Reviewer response for version 1

**DOI:** 10.21956/wellcomeopenres.22219.r69612

**Published:** 2024-05-11

**Authors:** Maria Mavroudi

**Affiliations:** 1 History, Classics, Middle Eastern Languages and Cultures, University of California, Berkeley, Berkeley, California, USA; 2 Department of Classics, University of California/Berkeley, Berkeley, CA, USA

This is an interesting experiment trying to exploit the possibilities provided by Artificial Intelligence in order to mine ancient and medieval Greek pharmacological texts for the purposes of history of pharmacology but also modern pharmacological research and make them accessible to readers who do not always have the linguistic competence to access them in the original. It is important to emphasize the historical context of the authors mentioned in order to appreciate continuities, discontinuities, and geographical factors that affect the Greek botanical vocabulary and the overall development of Greek botany. For example, Paul of Aegina lived in the seventh century and his writings were medieval best sellers in Greek, Arabic, and other languages. John the Physician is a thirteenth-century figure. 

 I like the fact that the authors are clear that "we are mostly offering an interpretation of a specific plant or mineral name". Anyone familiar with the problems of interpreting botanical and other technical terms in ancient and medieval texts in any language understands that this is a necessary precaution. 

 Another positive outcome of this experiment is the ability to better understand the order in which subjects are discussed in medical/botanical treatises (not always apparent, in spite of the general tendency to organize them from head to toe). And the ability to draw new connections between different disorders for which the same therapeutic approach is used.

Is the rationale for developing the new method (or application) clearly explained?

Yes

Is the description of the method technically sound?

Yes

Are the conclusions about the method and its performance adequately supported by the findings presented in the article?

Yes

If any results are presented, are all the source data underlying the results available to ensure full reproducibility?

Yes

Are sufficient details provided to allow replication of the method development and its use by others?

Yes

Reviewer Expertise:

History of science

I confirm that I have read this submission and believe that I have an appropriate level of expertise to confirm that it is of an acceptable scientific standard.
